# Supplementary material for: Impacts of Climate Change on the Biogeography of Three Amnesic Shellfish Toxin Producing Diatom Species
Source: Toxins (Basel). 2022 Dec 22;15(1):9. doi: 10.3390/toxins15010009 (PMC9863508; doi:10.3390/toxins15010009)
Supplement: Supplementary file 1 [file toxins-15-00009-s001.zip › toxins-2099991-supplementary/Supplementary Material/Evaluation/P_seriata/Pseudo_nitzchia_seriata.html]

Replicated maxent model for Pseudo\_nitzchia\_seriata


# Replicated maxent model for Pseudo\_nitzchia\_seriata

  
 This page summarizes the results of 5 split-sample models for Pseudo\_nitzchia\_seriata, created Mon May 30 09:33:21 BST 2022 using Maxent version 3.3.3a. The individual models are here: [0] [1] [2] [3] [4]  
  

---

## Analysis of omission/commission

The following picture shows the test omission rate and predicted area as a function of the cumulative threshold, averaged over the replicate runs. The omission rate should be close to the predicted omission, because of the definition of the cumulative threshold.
  
  
  
 The next picture is the receiver operating characteristic (ROC) curve for the same data, again averaged over the replicate runs. Note that the specificity is defined using predicted area, rather than true commission (see the paper by Phillips, Anderson and Schapire cited on the help page for discussion of what this means). The average test AUC for the replicate runs is 0.785, and the standard deviation is 0.009.
  
  
  

---

## Pictures of the model

  

---

## Analysis of variable contributions

  
The following table gives estimates of relative contributions of the environmental variables to the Maxent model. To determine the first estimate, in each iteration of the training algorithm, the increase in regularized gain is added to the contribution of the corresponding variable, or subtracted from it if the change to the absolute value of lambda is negative. For the second estimate, for each environmental variable in turn, the values of that variable on training presence and background data are randomly permuted. The model is reevaluated on the permuted data, and the resulting drop in training AUC is shown in the table, normalized to percentages. As with the variable jackknife, variable contributions should be interpreted with caution when the predictor variables are correlated. Values shown are averages over replicate runs.  
  

|  |  |  |  |  |  |  |  |  |  |  |  |  |  |  |  |  |  |  |  |  |  |  |  |  |  |  |  |  |  |  |  |  |  |  |  |  |  |  |  |  |  |
| --- | --- | --- | --- | --- | --- | --- | --- | --- | --- | --- | --- | --- | --- | --- | --- | --- | --- | --- | --- | --- | --- | --- | --- | --- | --- | --- | --- | --- | --- | --- | --- | --- | --- | --- | --- | --- | --- | --- | --- | --- | --- |
| Variable | Percent contribution | Permutation importance || Bathy | 81.9 | 74.1 |
| TempMin | 9.5 | 9.6 |
| TempRange | 4.7 | 6.3 |
| TempMean | 0.9 | 2.1 |
| SalMax | 0.8 | 0.8 |
| TempMax | 0.6 | 1.5 |
| SalMean | 0.3 | 0.9 |
| SalRange | 0.3 | 0.7 |
| CVMin | 0.2 | 0.7 |
| CVMax | 0.2 | 1.4 |
| CVRange | 0.2 | 0.6 |
| SalMin | 0.2 | 0.5 |
| CVMean | 0 | 0.8 |

  
  
The following picture shows the results of the jackknife test of variable importance. The environmental variable with highest gain when used in isolation is Bathy, which therefore appears to have the most useful information by itself. The environmental variable that decreases the gain the most when it is omitted is Bathy, which therefore appears to have the most information that isn't present in the other variables. Values shown are averages over replicate runs.  
  
  
  
The next picture shows the same jackknife test, using test gain instead of training gain. Note that conclusions about which variables are most important can change, now that we're looking at test data.
  
  
  
Lastly, we have the same jackknife test, using AUC on test data.
  
  
  

---

  
Command line to repeat this species model: java density.MaxEnt nowarnings noprefixes -E "" -E Pseudo\_nitzchia\_seriata jackknife outputdirectory=models/Pseudo\_nitzchia\_seriata samplesfile=occurrences/Pseudo\_nitzchia\_seriata.csv environmentallayers=backgrounds/Gymnodinium\_catenatum\_background.csv randomseed nowarnings noaskoverwrite randomtestpoints=20 replicates=5 replicatetype=subsample autorun writeplotdata  
