# Supplementary figures and images for: Impacts of Climate Change on the Biogeography of Three Amnesic Shellfish Toxin Producing Diatom Species
Source: Toxins (Basel). 2022 Dec 22;15(1):9. doi: 10.3390/toxins15010009 (PMC9863508; doi:10.3390/toxins15010009)

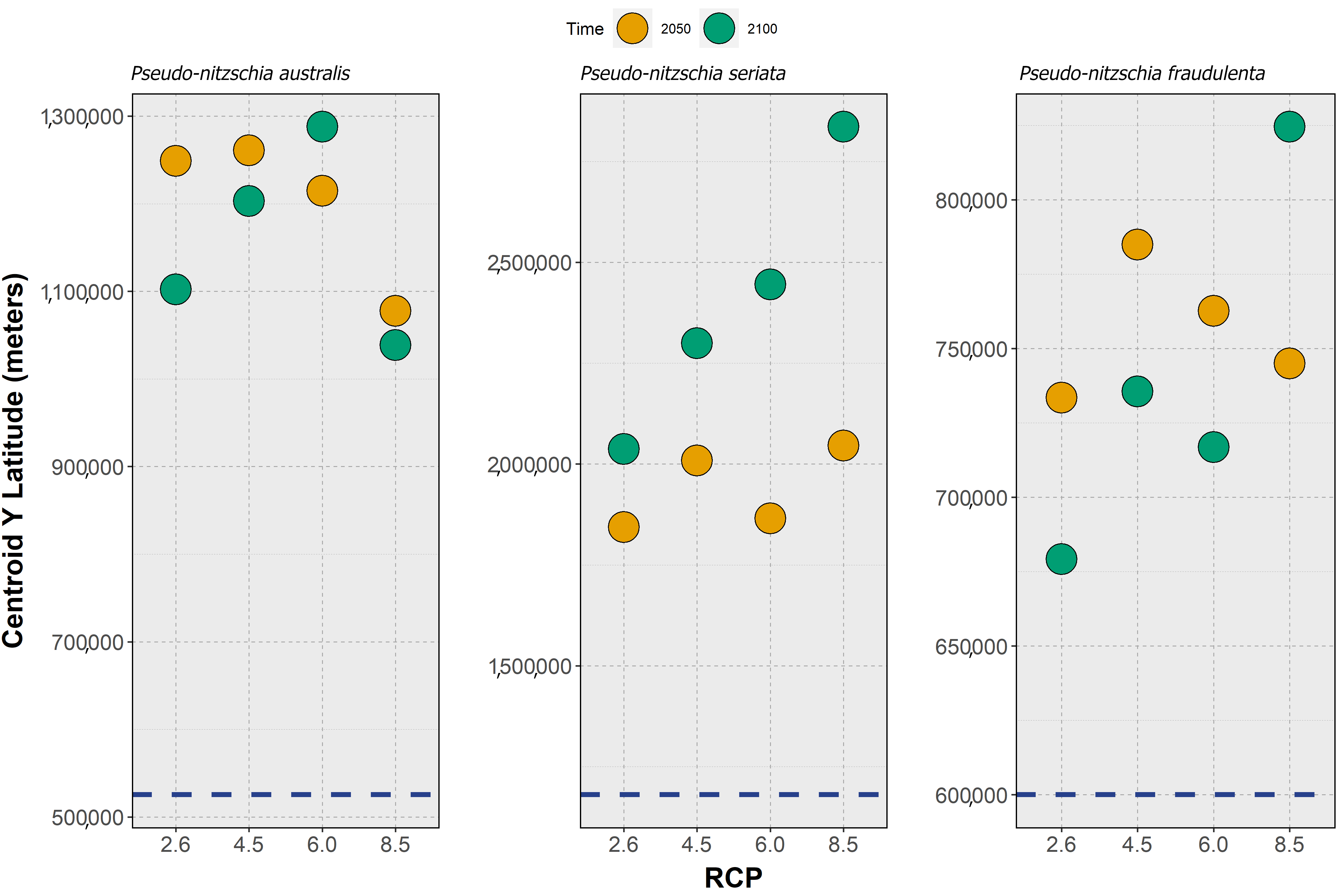

Supplement: Supplementary file 1 [file toxins-15-00009-s001.zip › toxins-2099991-supplementary/Supplementary Material/High_resolution_Figures/Figure 1 - centroids.png]

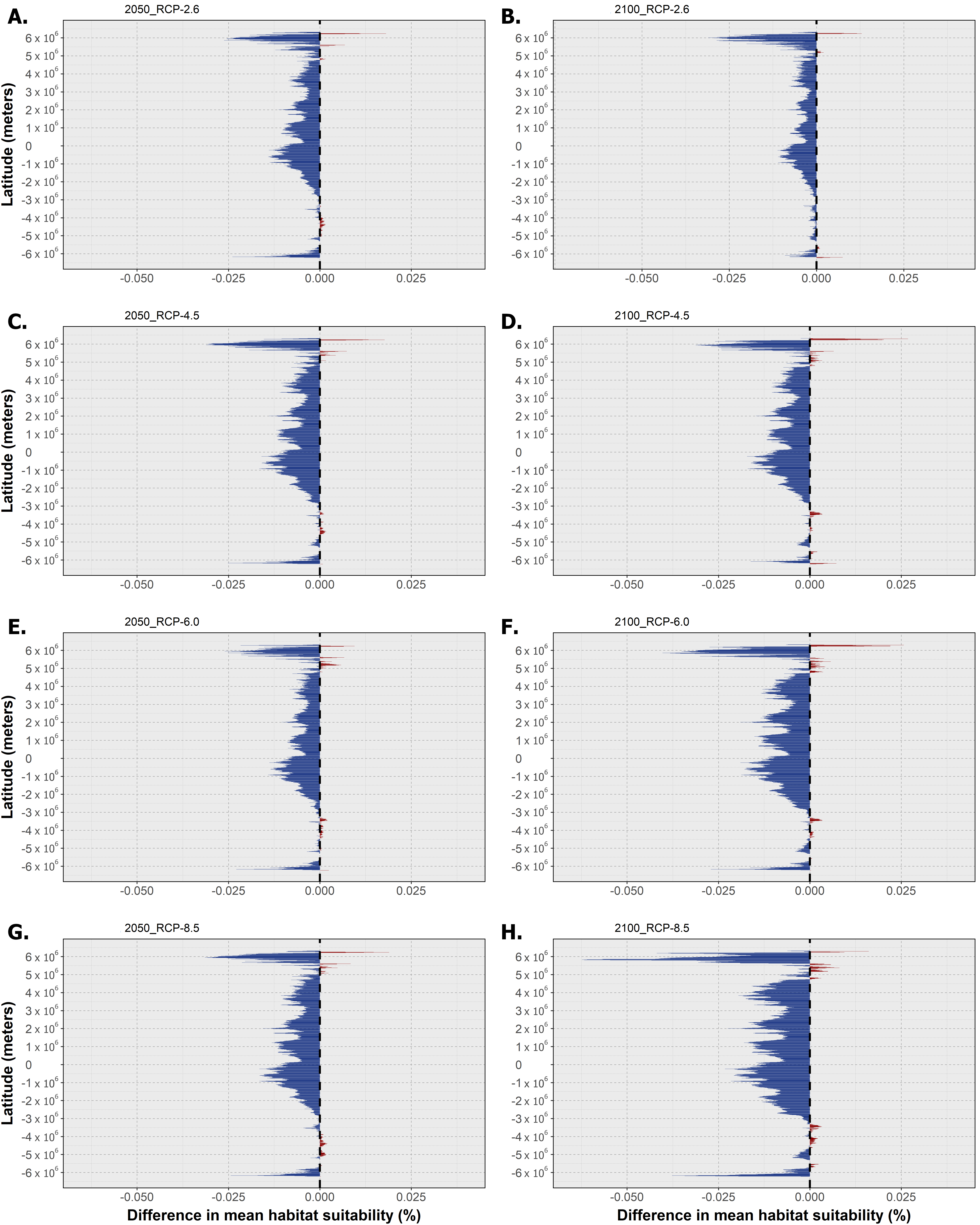

Supplement: Supplementary file 1 [file toxins-15-00009-s001.zip › toxins-2099991-supplementary/Supplementary Material/High_resolution_Figures/Figure 2 - australis.png]

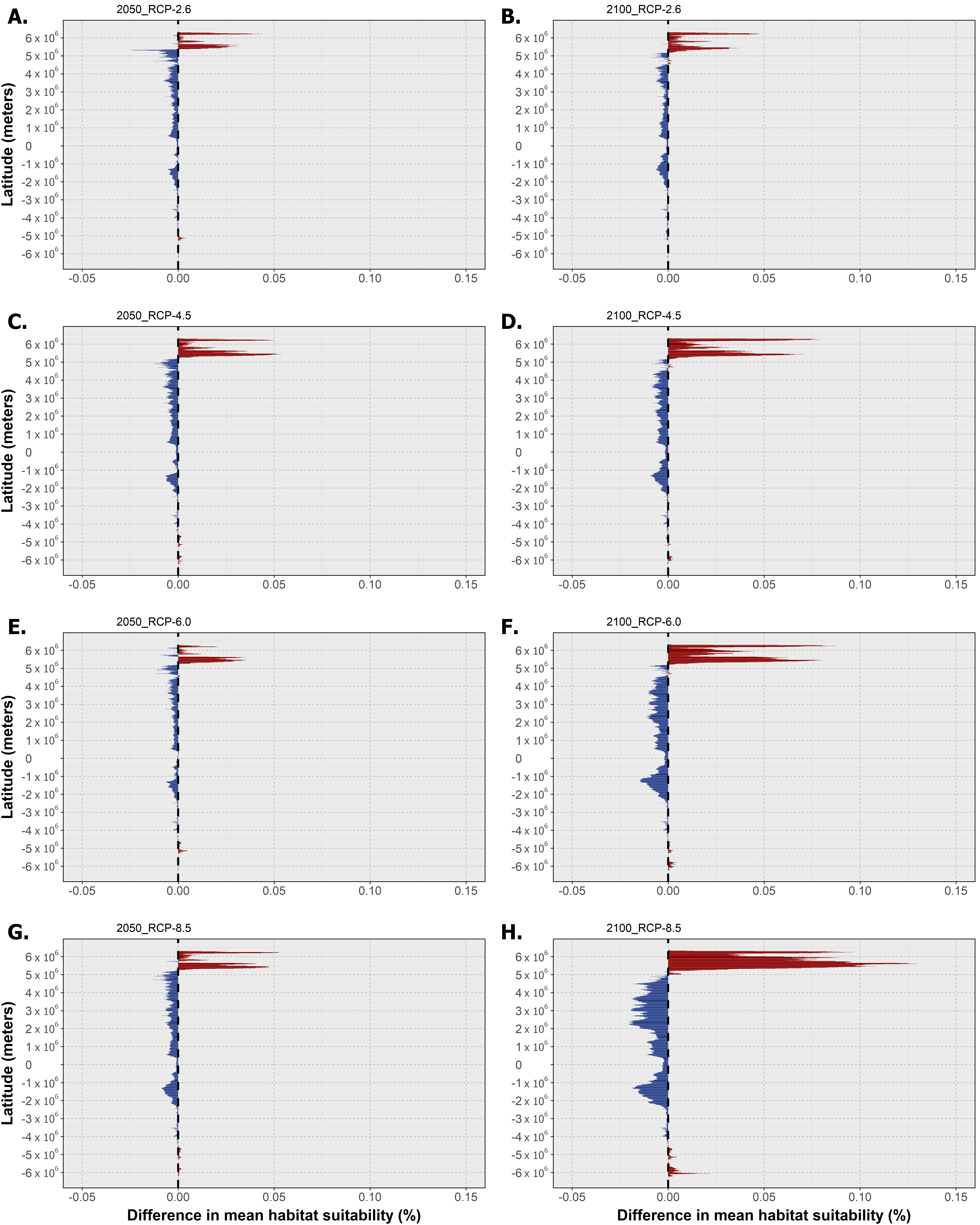

Supplement: Supplementary file 1 [file toxins-15-00009-s001.zip › toxins-2099991-supplementary/Supplementary Material/High_resolution_Figures/Figure 4 - seriata.png]

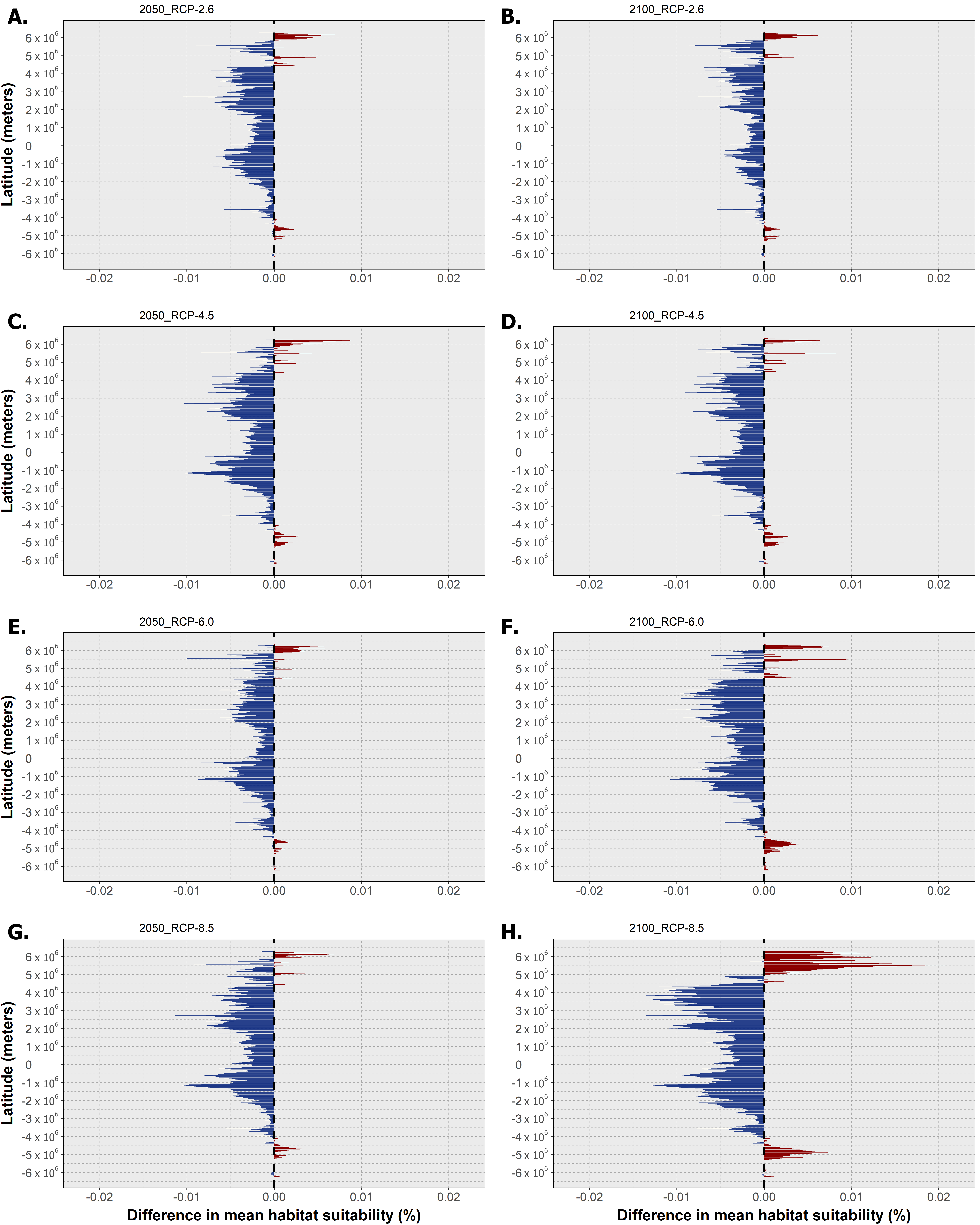

Supplement: Supplementary file 1 [file toxins-15-00009-s001.zip › toxins-2099991-supplementary/Supplementary Material/High_resolution_Figures/Figure 6 - fraudulenta.png]

# Pseudo-nitzschia\_australis\_2050\_RCP2.6\_binary

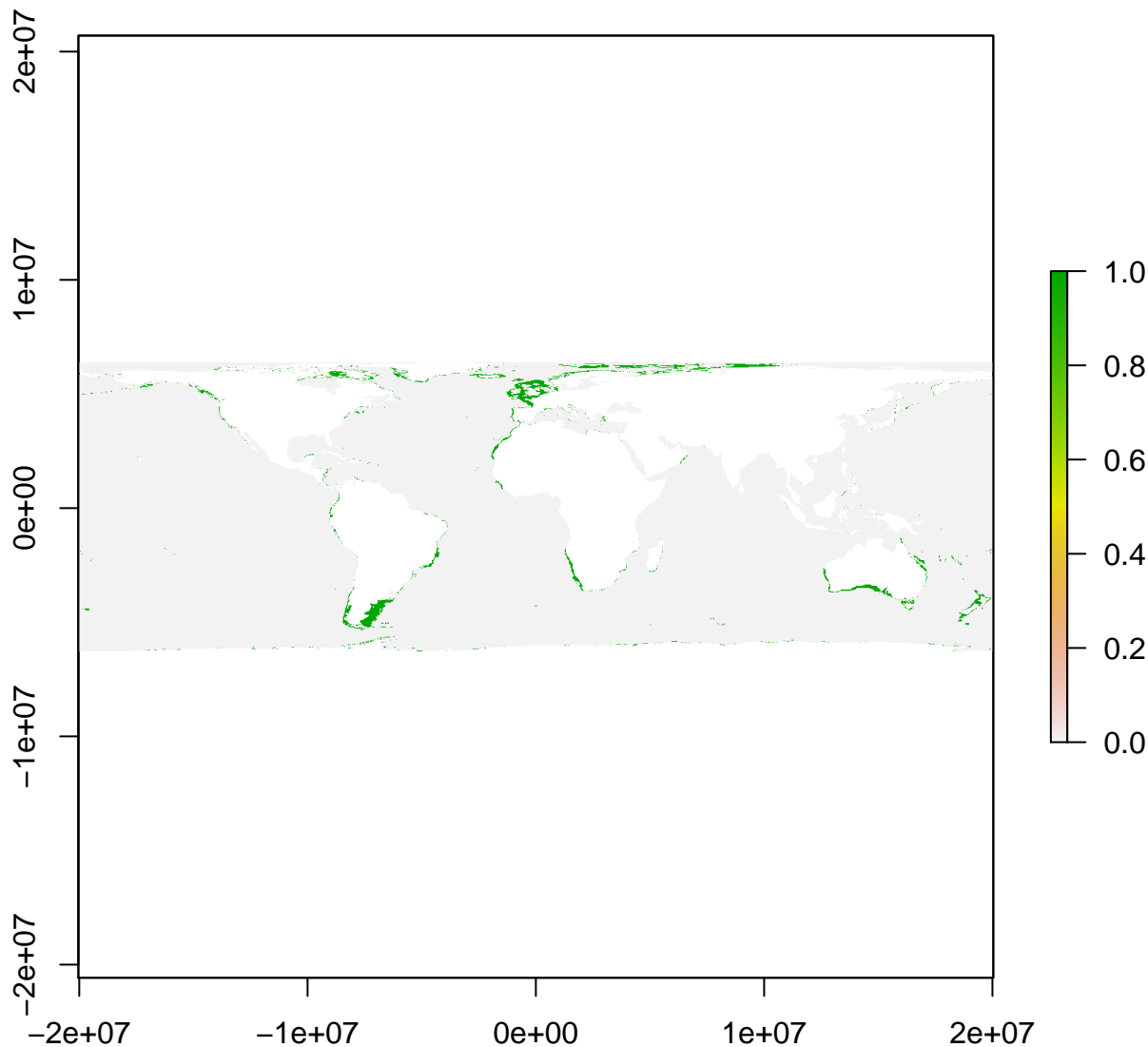

Supplement: Supplementary file 1 [file toxins-15-00009-s001.zip › toxins-2099991-supplementary/Supplementary Material/Projections/Binary/australis/2050_RCP2.6_binary.pdf]

# Pseudo-nitzschia\_australis\_2050\_RCP4.5\_binary

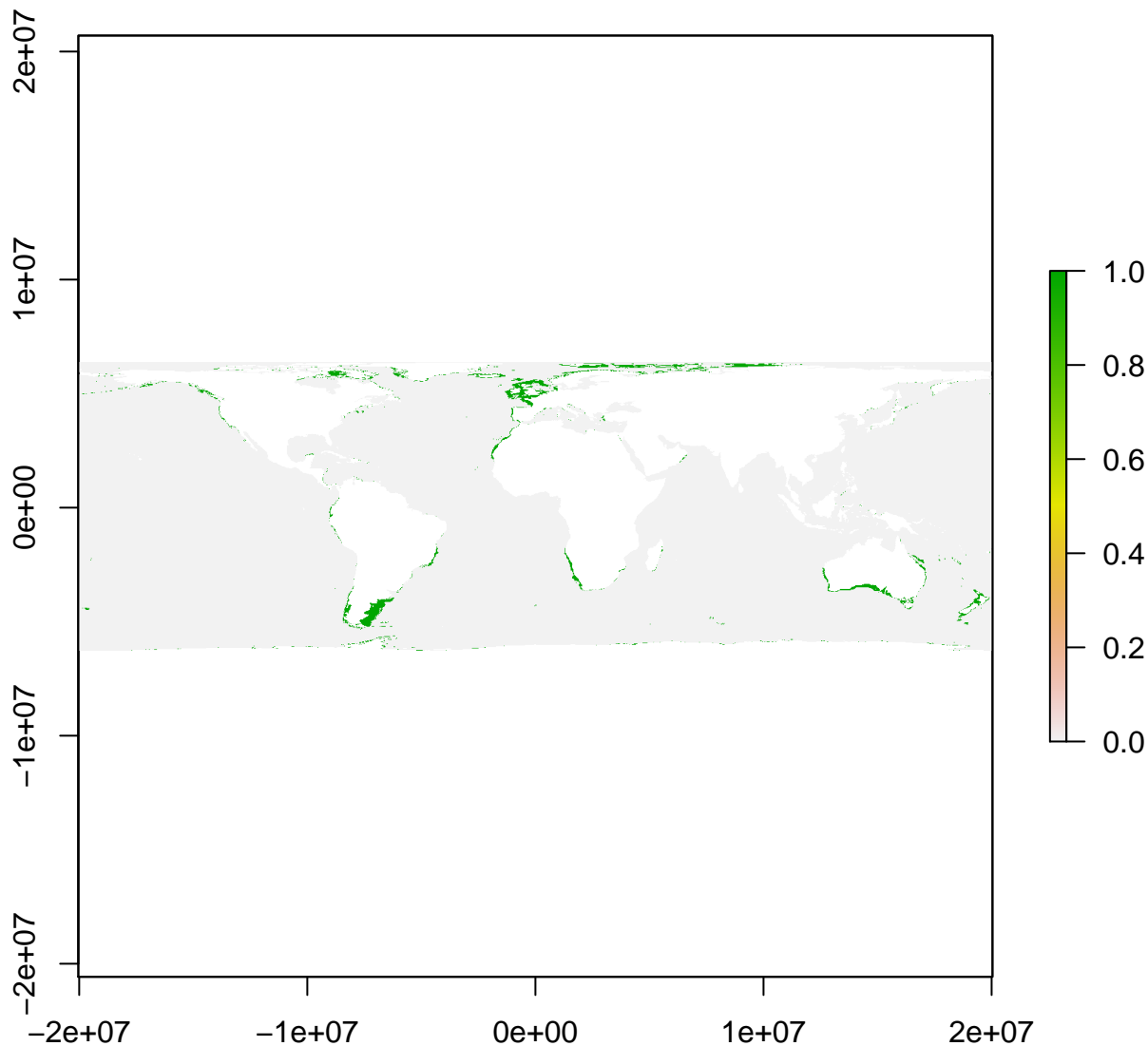

Supplement: Supplementary file 1 [file toxins-15-00009-s001.zip › toxins-2099991-supplementary/Supplementary Material/Projections/Binary/australis/2050_RCP4.5_binary.pdf]

# Pseudo-nitzschia\_australis\_2050\_RCP6.0\_binary

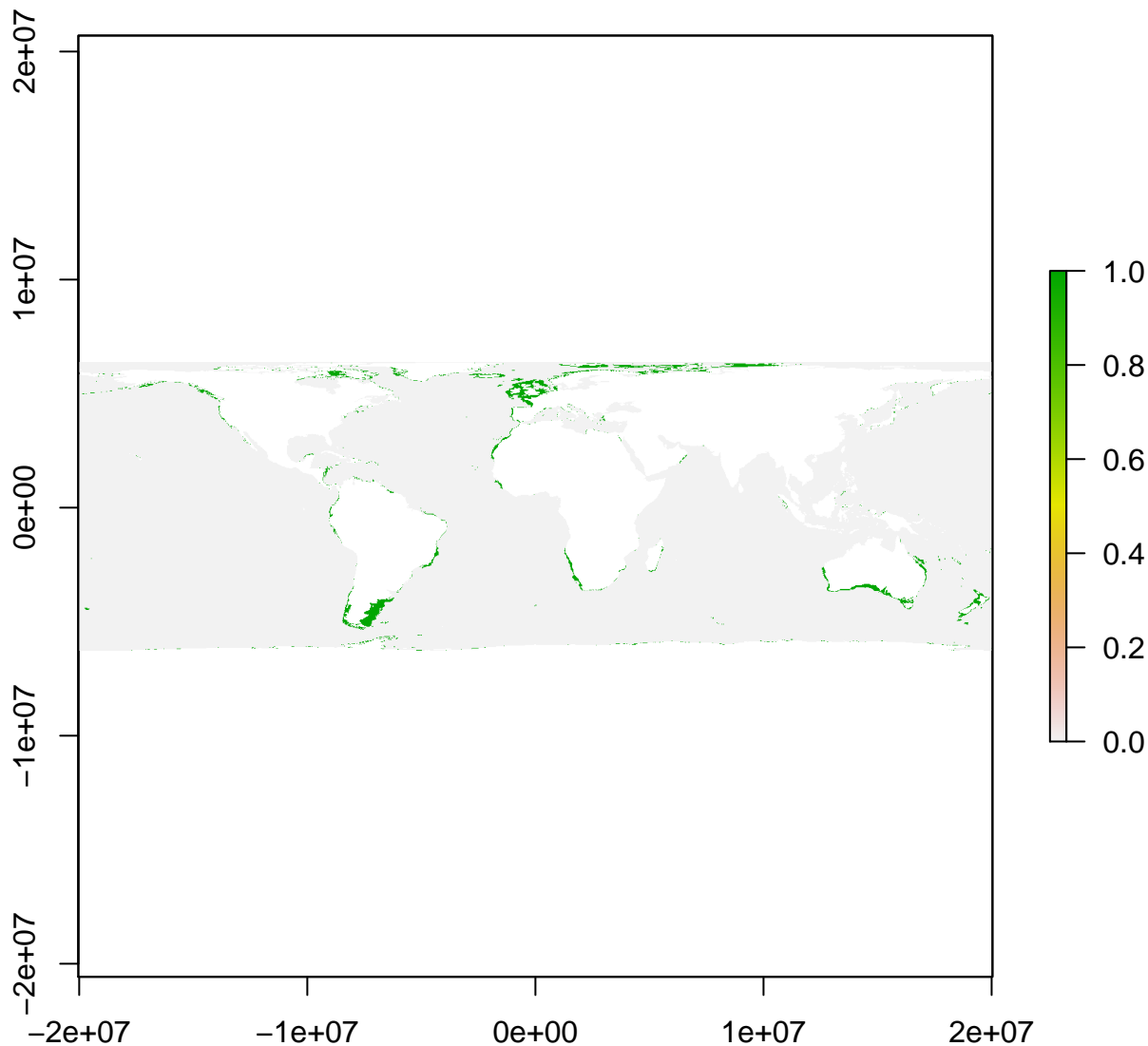

Supplement: Supplementary file 1 [file toxins-15-00009-s001.zip › toxins-2099991-supplementary/Supplementary Material/Projections/Binary/australis/2050_RCP6.0_binary.pdf]

# Pseudo-nitzschia\_australis\_2050\_RCP8.5\_binary

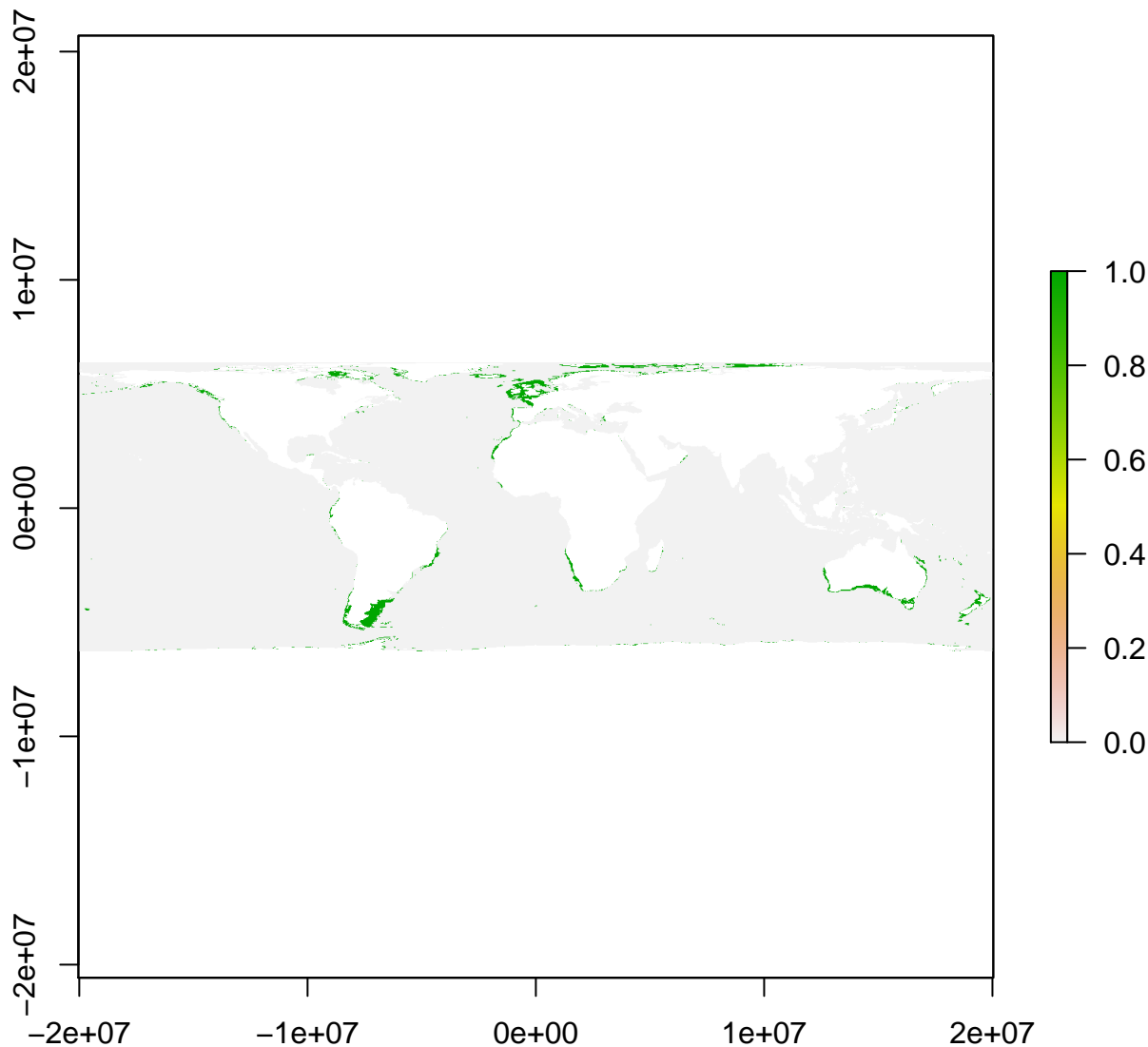

Supplement: Supplementary file 1 [file toxins-15-00009-s001.zip › toxins-2099991-supplementary/Supplementary Material/Projections/Binary/australis/2050_RCP8.5_binary.pdf]

# Pseudo-nitzschia\_australis\_2100\_RCP2.6\_binary

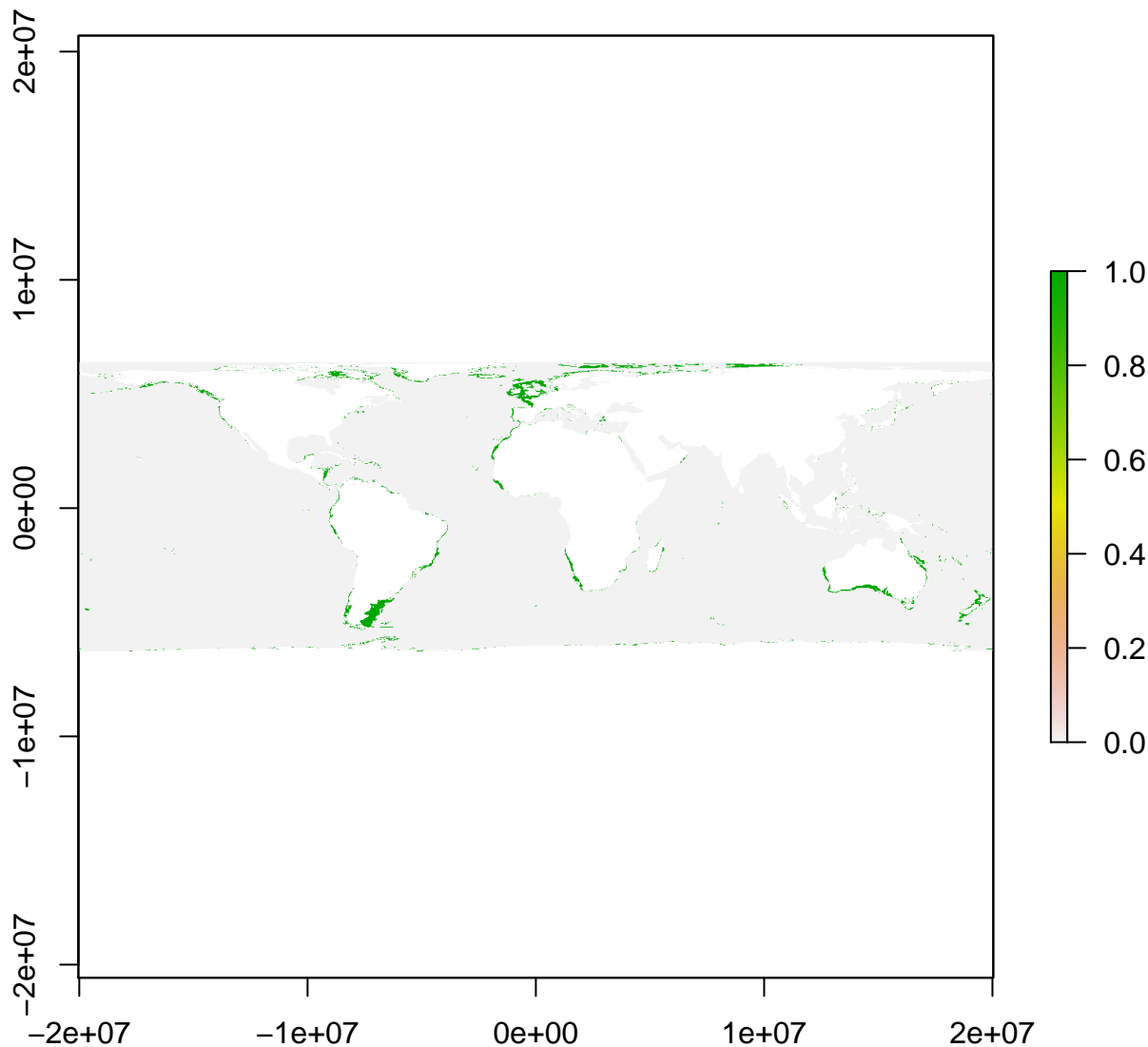

Supplement: Supplementary file 1 [file toxins-15-00009-s001.zip › toxins-2099991-supplementary/Supplementary Material/Projections/Binary/australis/2100_RCP2.6_binary.pdf]

# Pseudo-nitzschia\_australis\_2100\_RCP4.5\_binary

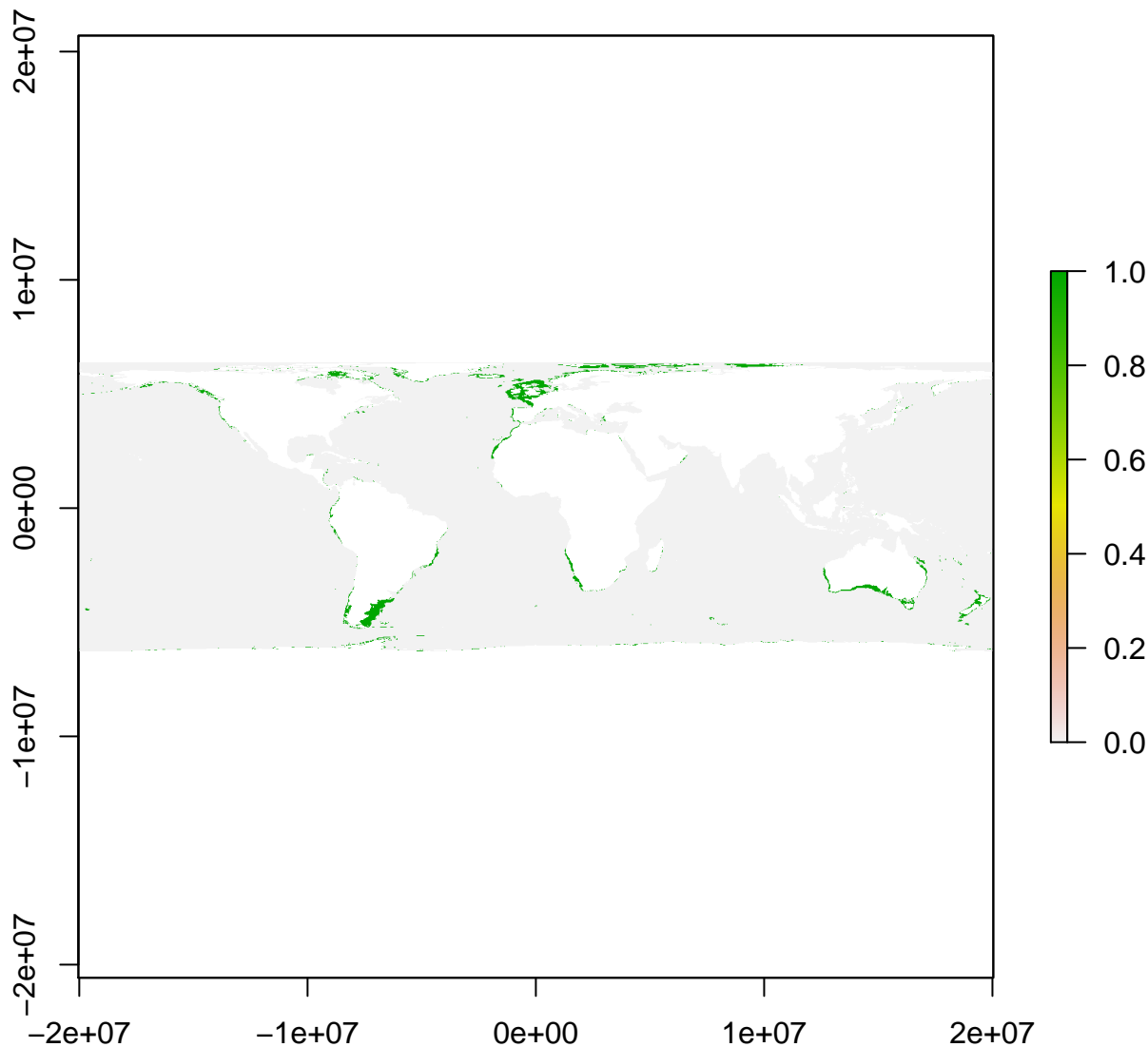

Supplement: Supplementary file 1 [file toxins-15-00009-s001.zip › toxins-2099991-supplementary/Supplementary Material/Projections/Binary/australis/2100_RCP4.5_binary.pdf]

# Pseudo-nitzschia\_australis\_2100\_RCP6.0\_binary

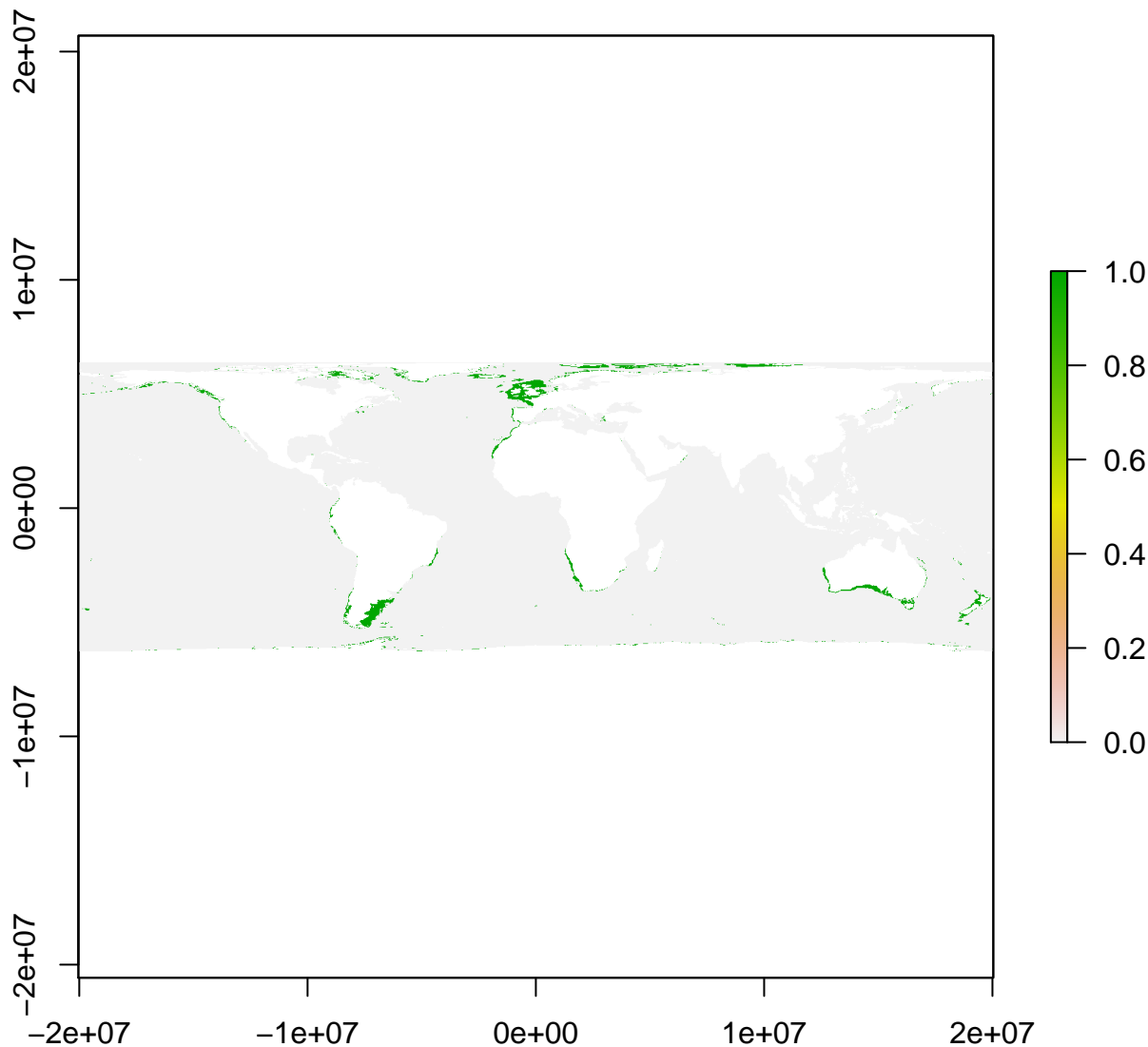

Supplement: Supplementary file 1 [file toxins-15-00009-s001.zip › toxins-2099991-supplementary/Supplementary Material/Projections/Binary/australis/2100_RCP6.0_binary.pdf]

# Pseudo-nitzschia\_australis\_2100\_RCP8.5\_binary

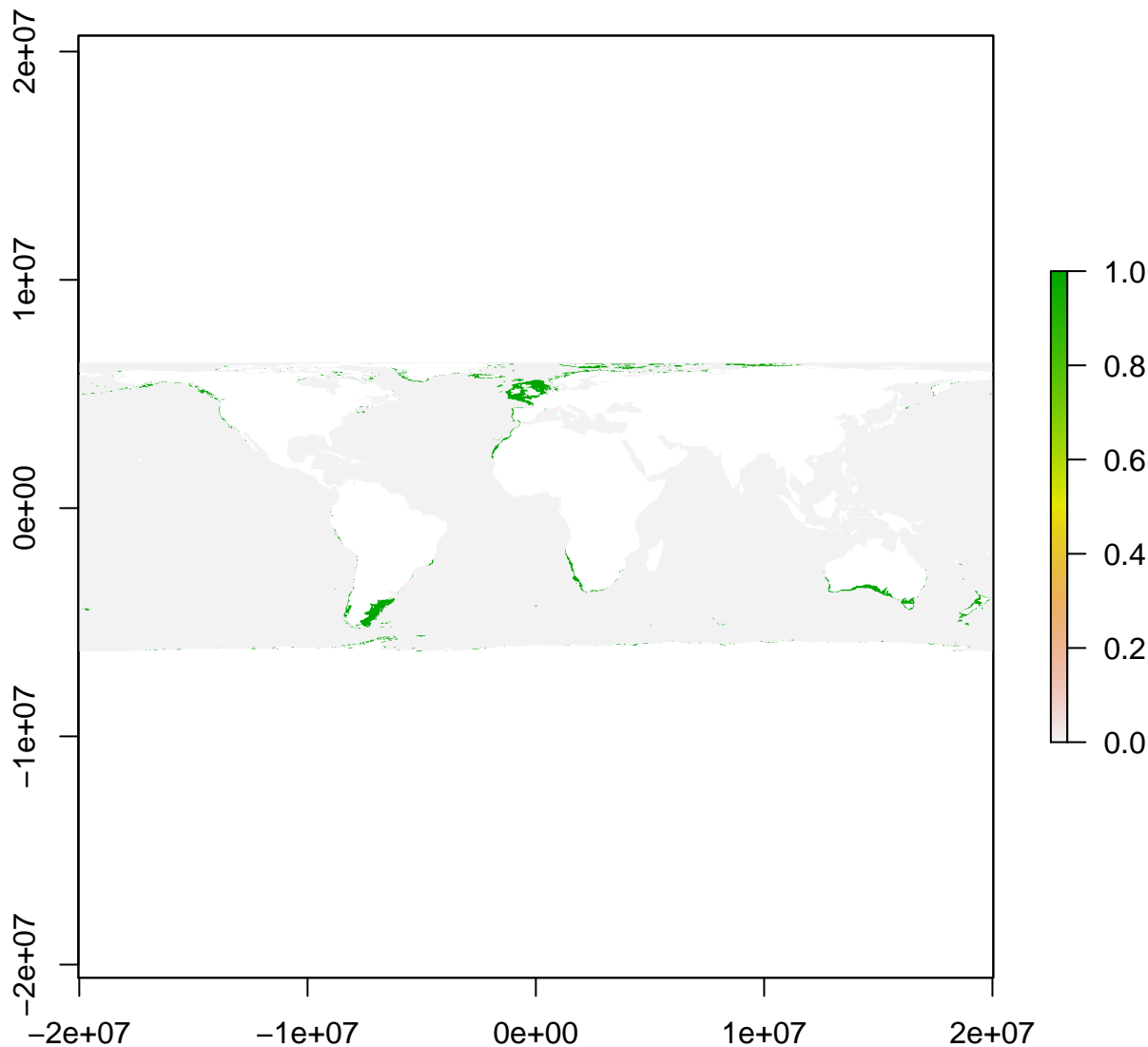

Supplement: Supplementary file 1 [file toxins-15-00009-s001.zip › toxins-2099991-supplementary/Supplementary Material/Projections/Binary/australis/2100_RCP8.5_binary.pdf]

# Pseudo-nitzschia\_australis\_present\_binary

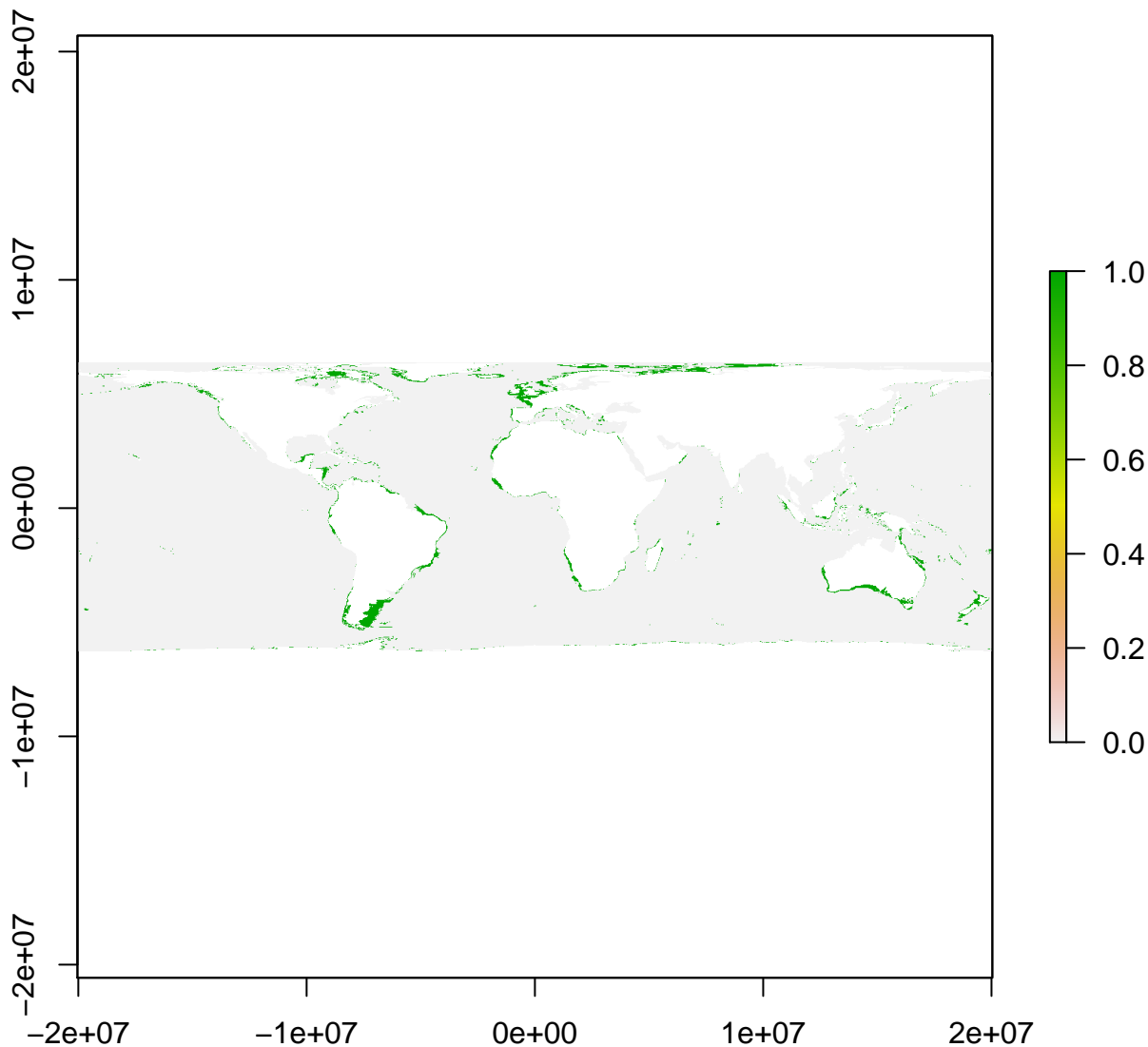

Supplement: Supplementary file 1 [file toxins-15-00009-s001.zip › toxins-2099991-supplementary/Supplementary Material/Projections/Binary/australis/present_binary.pdf]

# Pseudo\_nitzchia\_fraudulenta\_2050\_RCP2.6\_binary

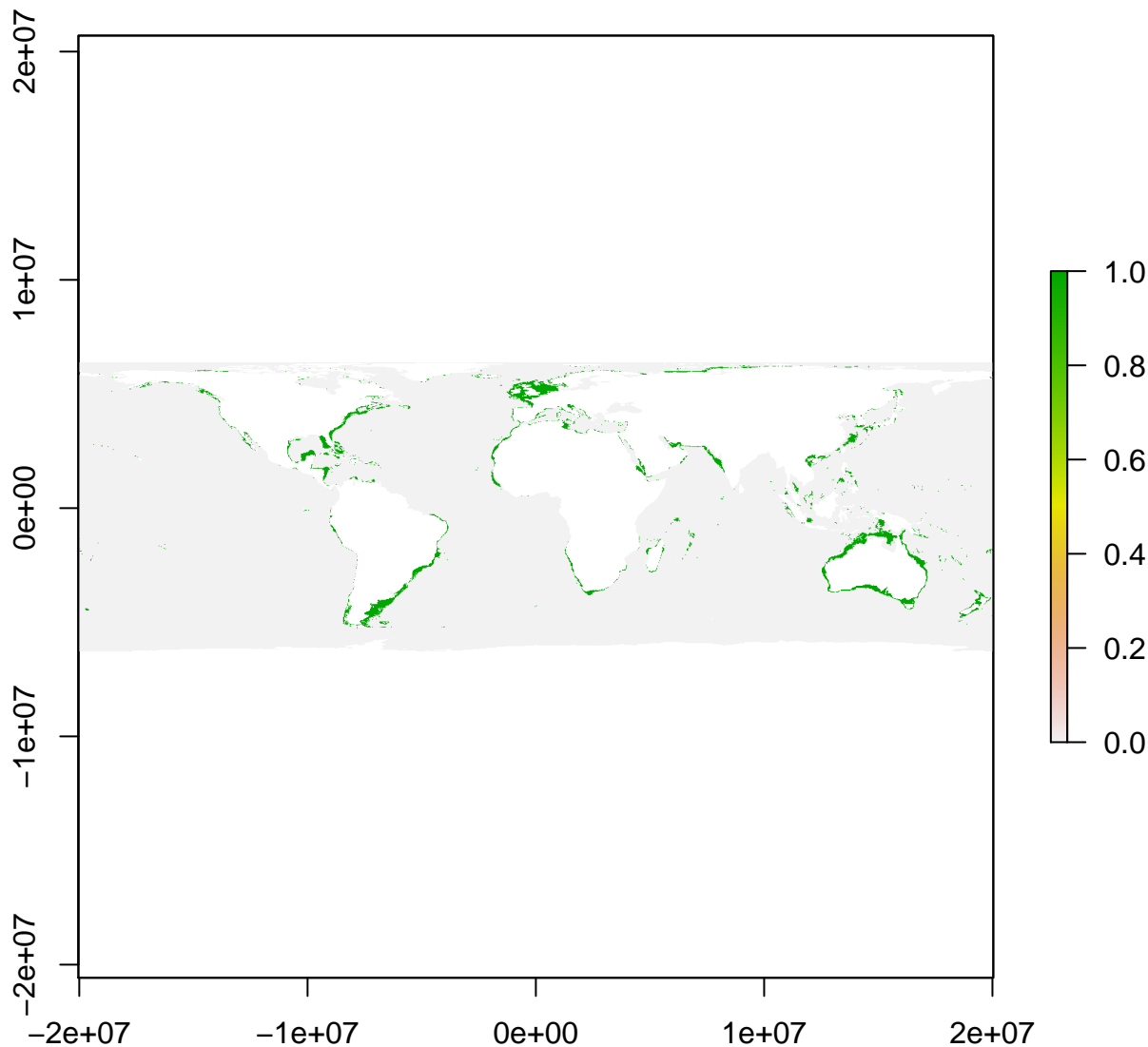

Supplement: Supplementary file 1 [file toxins-15-00009-s001.zip › toxins-2099991-supplementary/Supplementary Material/Projections/Binary/fraudulenta/2050_RCP2.6_binary.pdf]

# Pseudo\_nitzchia\_fraudulenta\_2050\_RCP4.5\_binary

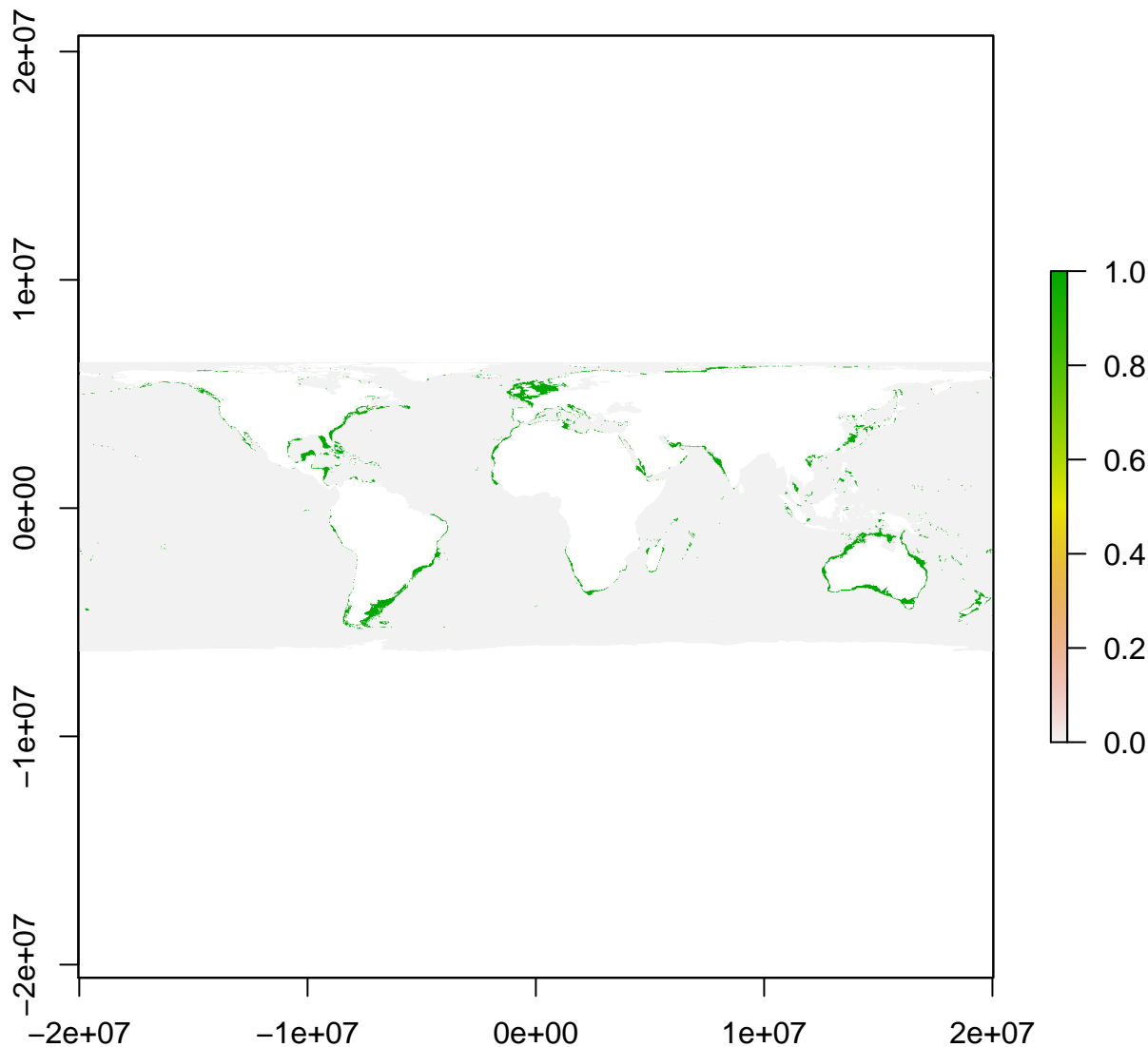

Supplement: Supplementary file 1 [file toxins-15-00009-s001.zip › toxins-2099991-supplementary/Supplementary Material/Projections/Binary/fraudulenta/2050_RCP4.5_binary.pdf]

# Pseudo\_nitzchia\_fraudulenta\_2050\_RCP6.0\_binary

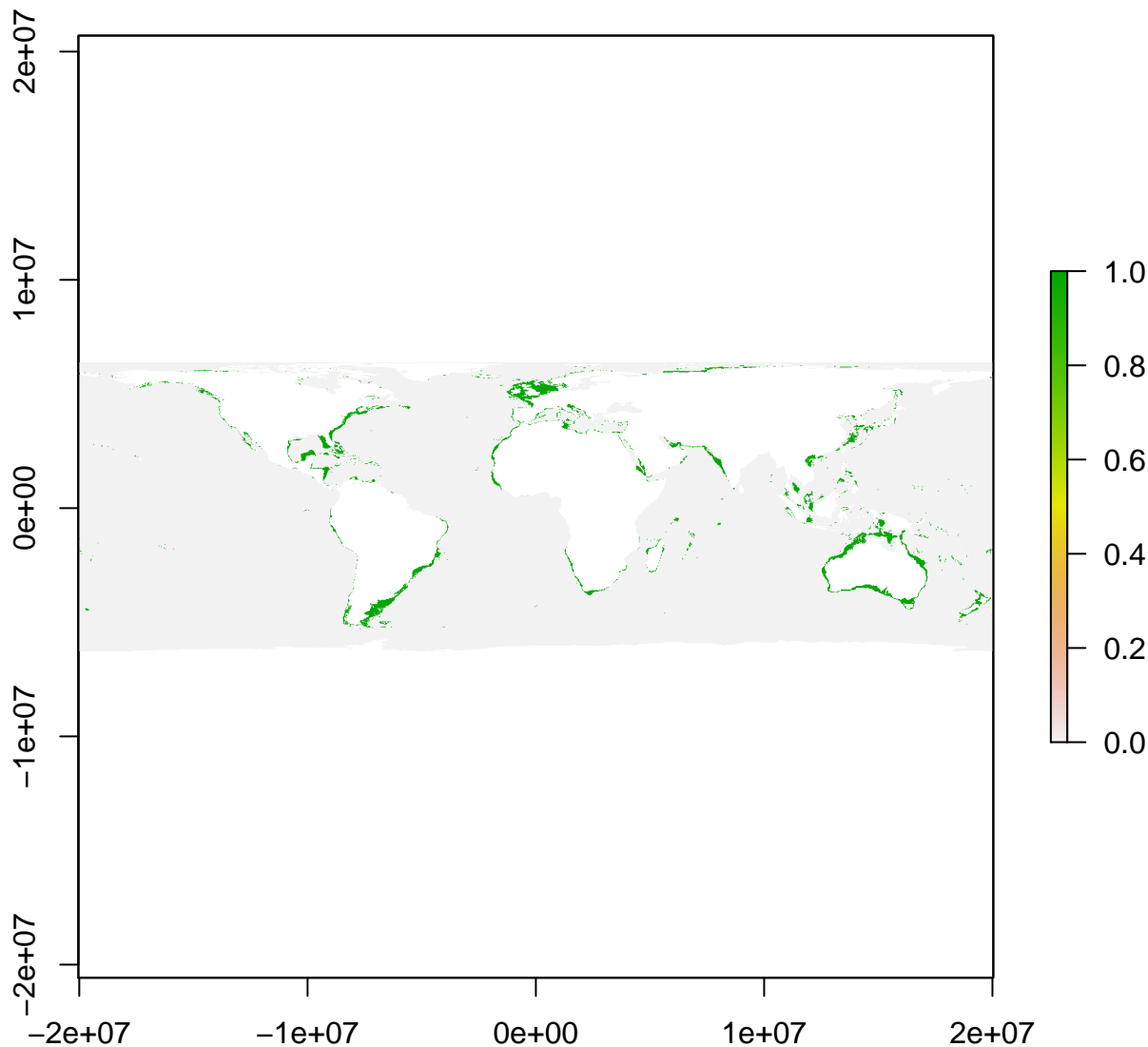

Supplement: Supplementary file 1 [file toxins-15-00009-s001.zip › toxins-2099991-supplementary/Supplementary Material/Projections/Binary/fraudulenta/2050_RCP6.0_binary.pdf]

# Pseudo\_nitzchia\_fraudulenta\_2050\_RCP8.5\_binary

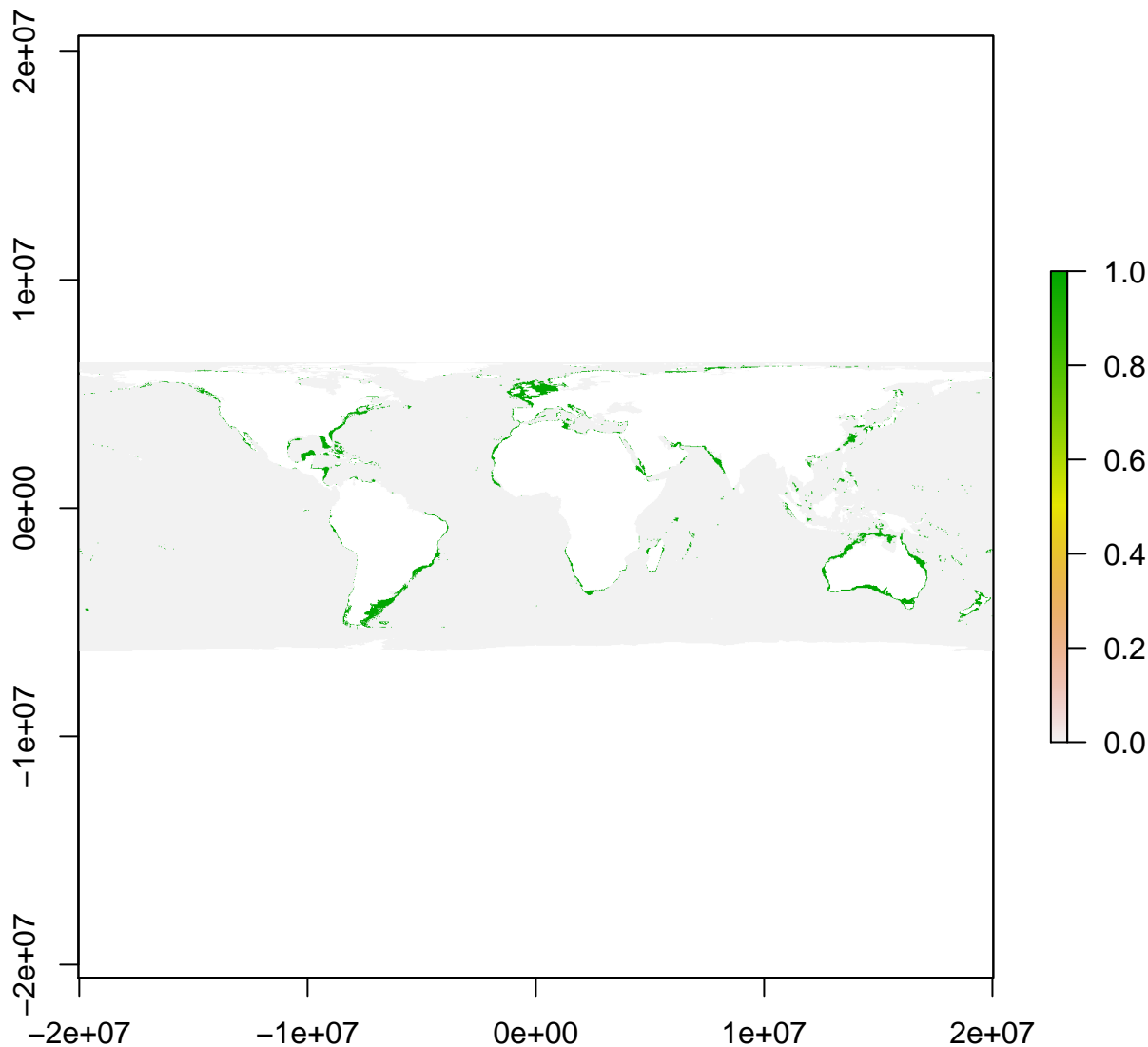

Supplement: Supplementary file 1 [file toxins-15-00009-s001.zip › toxins-2099991-supplementary/Supplementary Material/Projections/Binary/fraudulenta/2050_RCP8.5_binary.pdf]

# Pseudo\_nitzchia\_fraudulenta\_2100\_RCP2.6\_binary

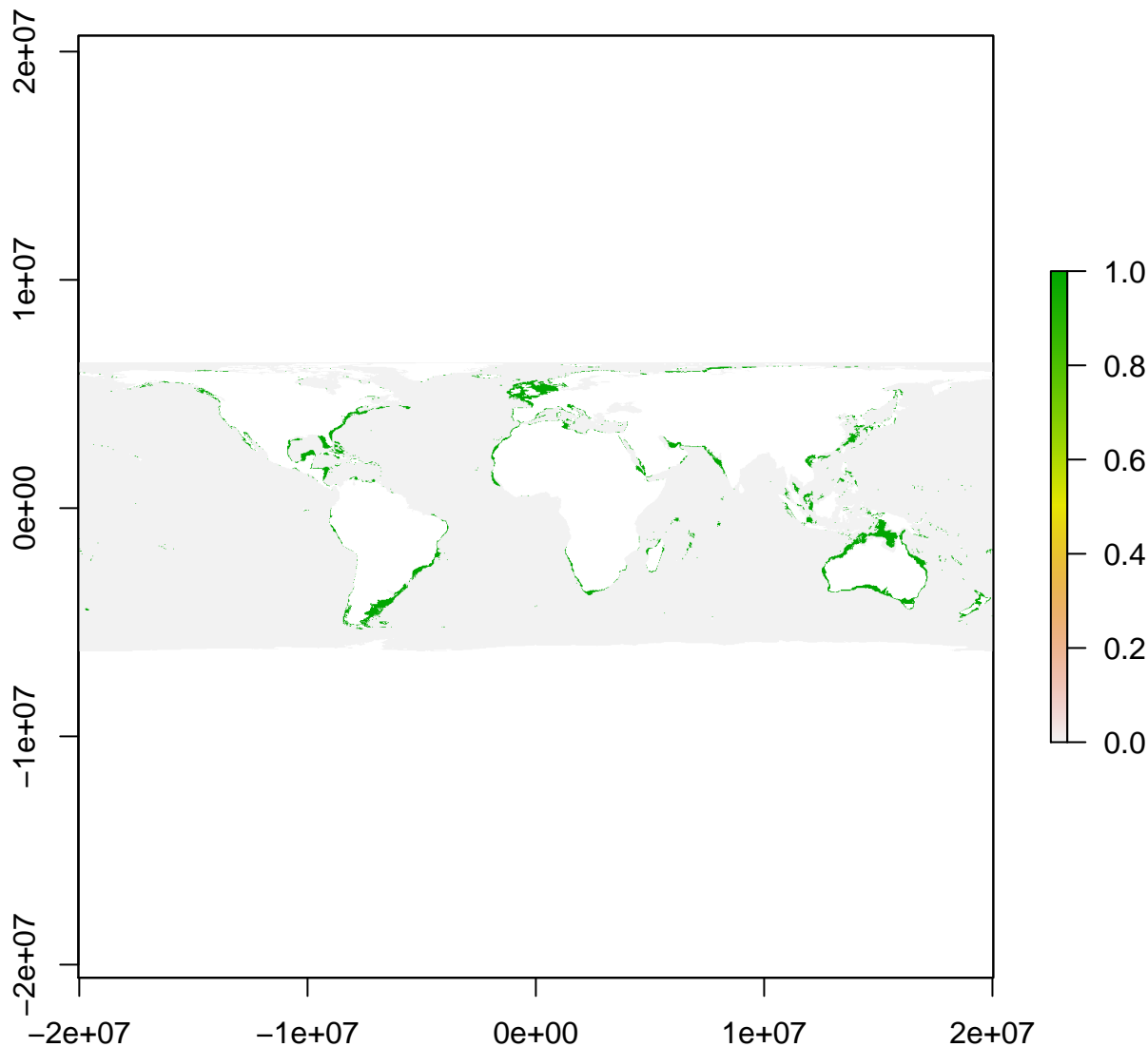

Supplement: Supplementary file 1 [file toxins-15-00009-s001.zip › toxins-2099991-supplementary/Supplementary Material/Projections/Binary/fraudulenta/2100_RCP2.6_binary.pdf]

# Pseudo\_nitzchia\_fraudulenta\_2100\_RCP4.5\_binary

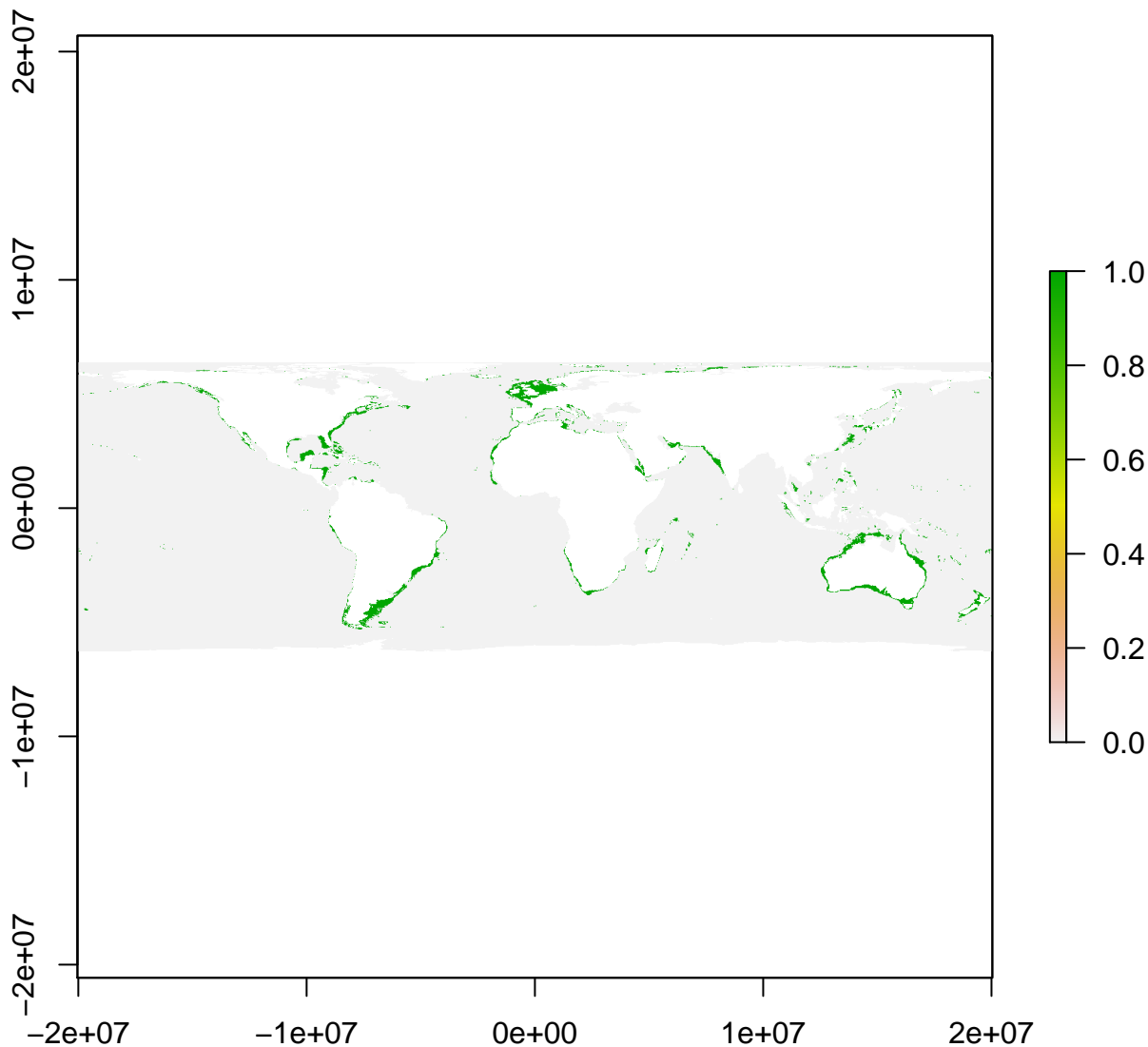

Supplement: Supplementary file 1 [file toxins-15-00009-s001.zip › toxins-2099991-supplementary/Supplementary Material/Projections/Binary/fraudulenta/2100_RCP4.5_binary.pdf]

# Pseudo\_nitzchia\_fraudulenta\_2100\_RCP6.0\_binary

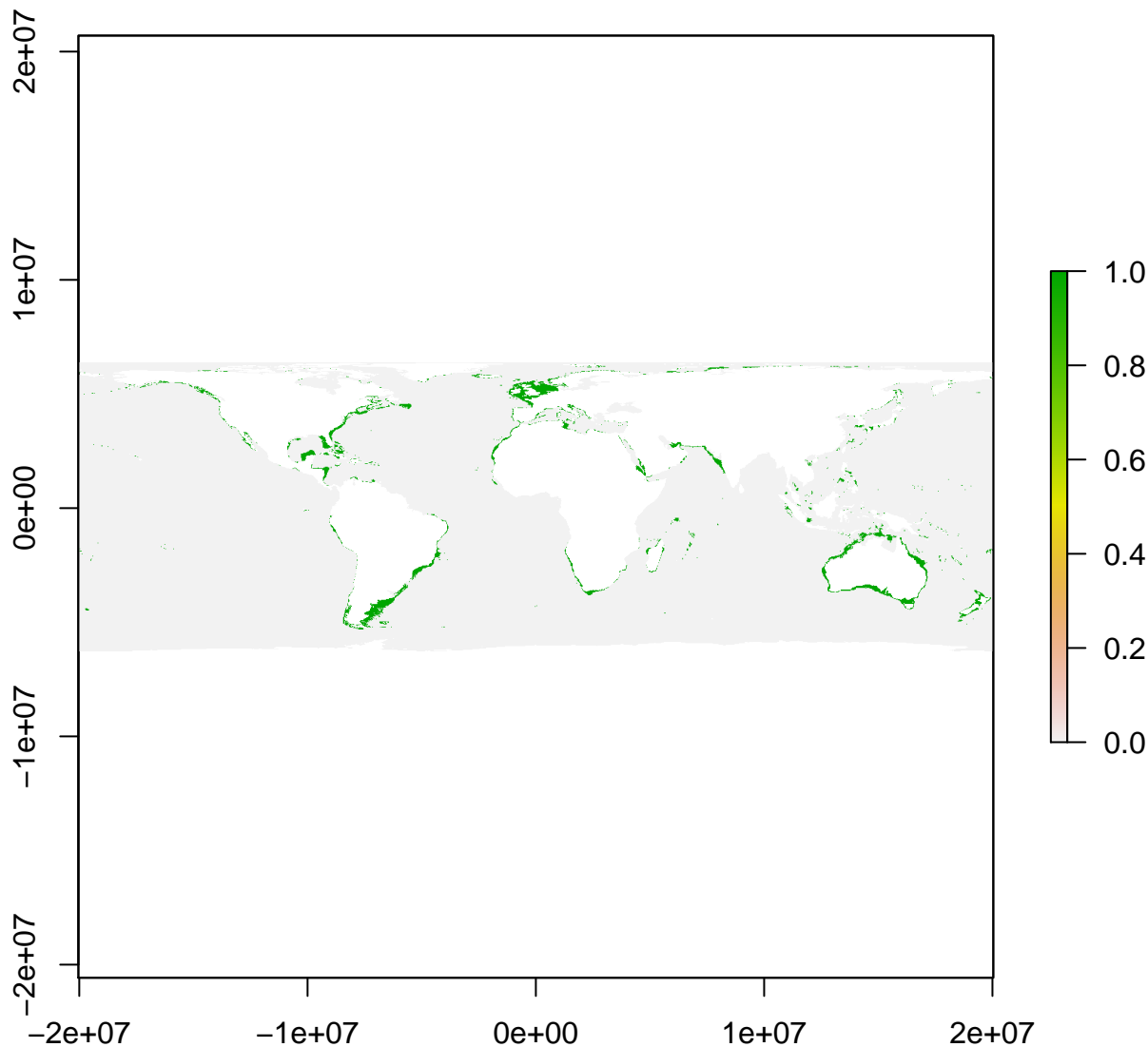

Supplement: Supplementary file 1 [file toxins-15-00009-s001.zip › toxins-2099991-supplementary/Supplementary Material/Projections/Binary/fraudulenta/2100_RCP6.0_binary.pdf]

# Pseudo\_nitzchia\_fraudulenta\_2100\_RCP8.5\_binary

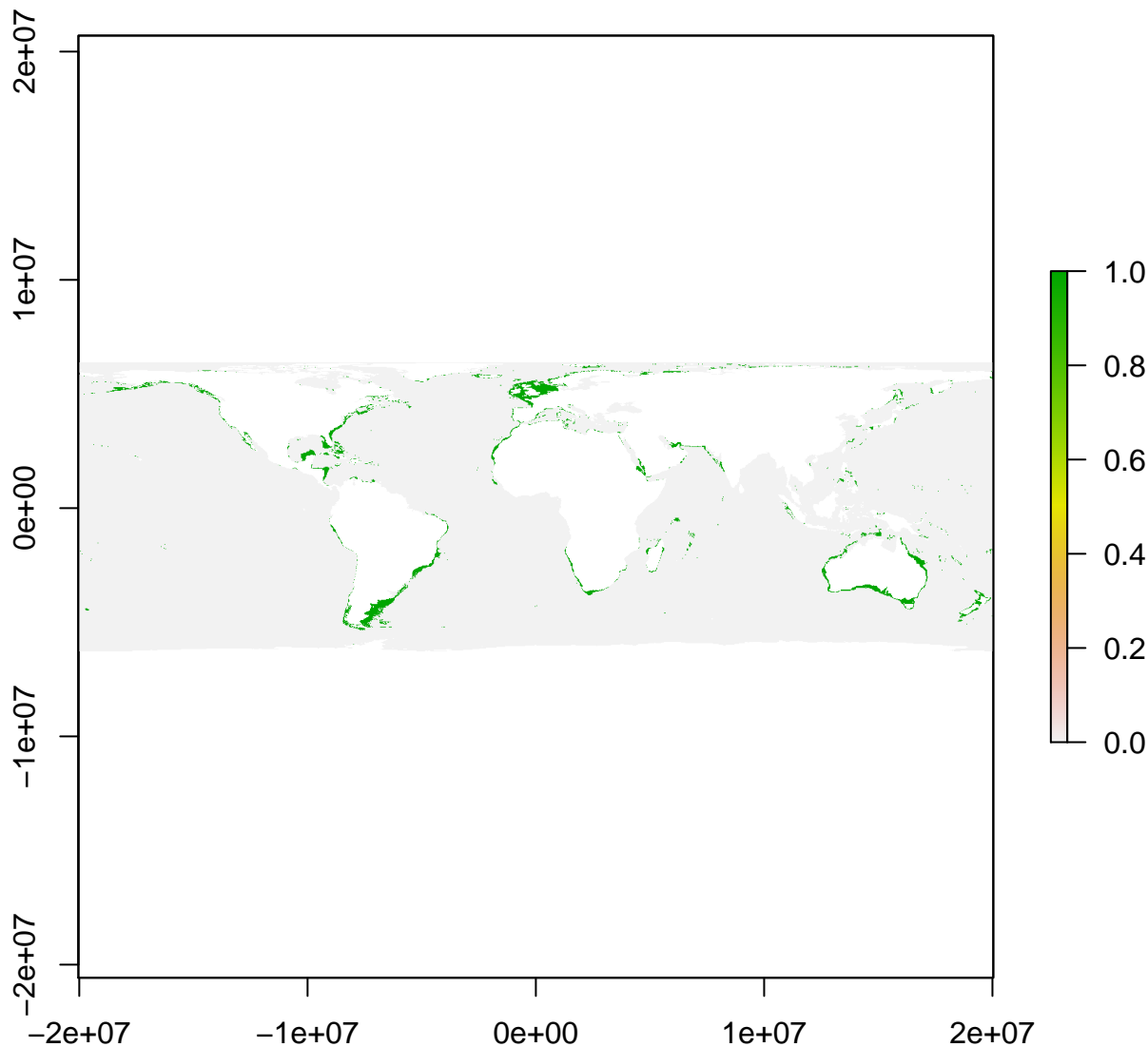

Supplement: Supplementary file 1 [file toxins-15-00009-s001.zip › toxins-2099991-supplementary/Supplementary Material/Projections/Binary/fraudulenta/2100_RCP8.5_binary.pdf]

# Pseudo\_nitzchia\_fraudulenta\_present\_binary

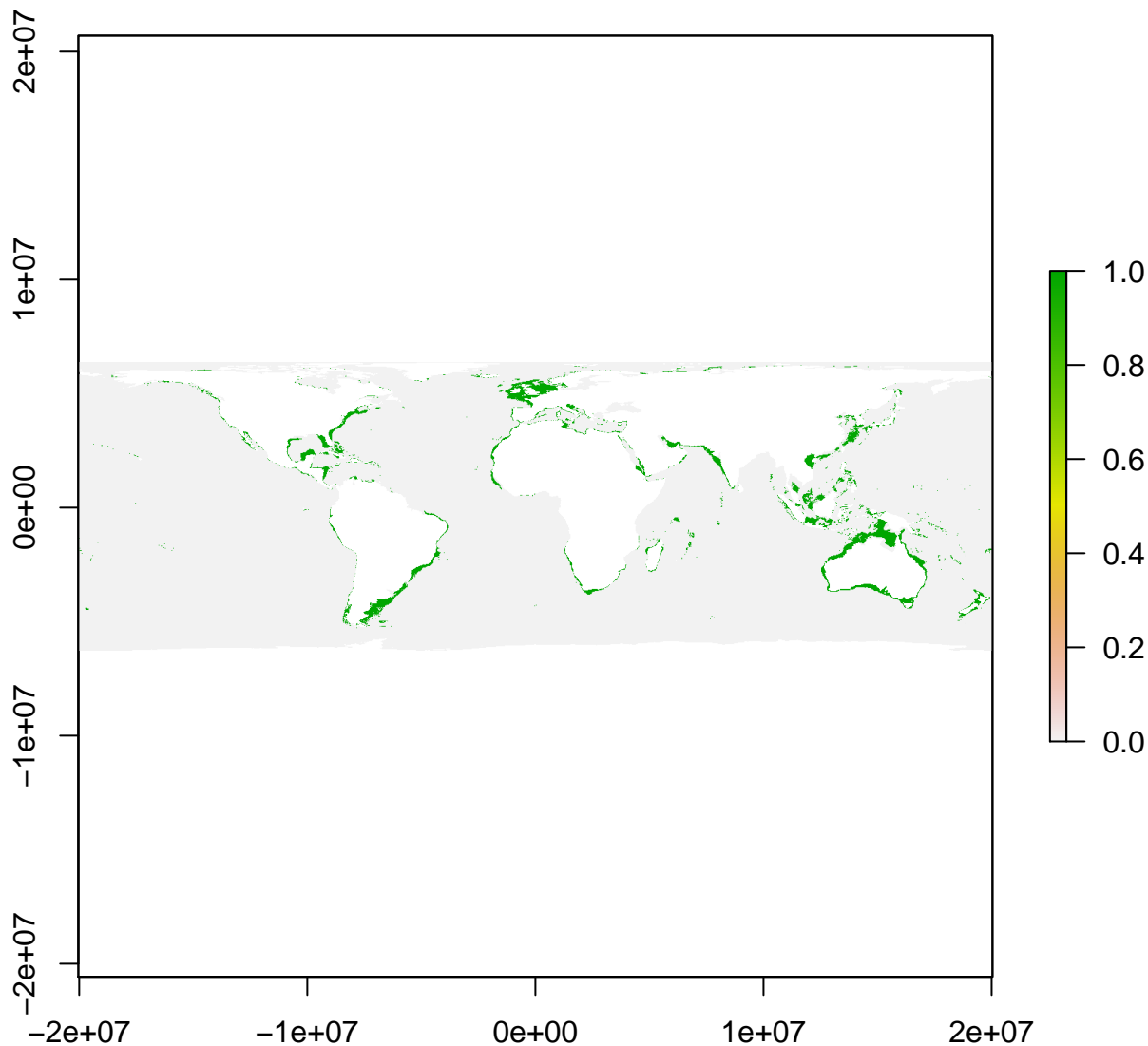

Supplement: Supplementary file 1 [file toxins-15-00009-s001.zip › toxins-2099991-supplementary/Supplementary Material/Projections/Binary/fraudulenta/present_binary.pdf]

# Pseudo\_nitzchia\_serjata\_2050\_RCP2.6\_binary

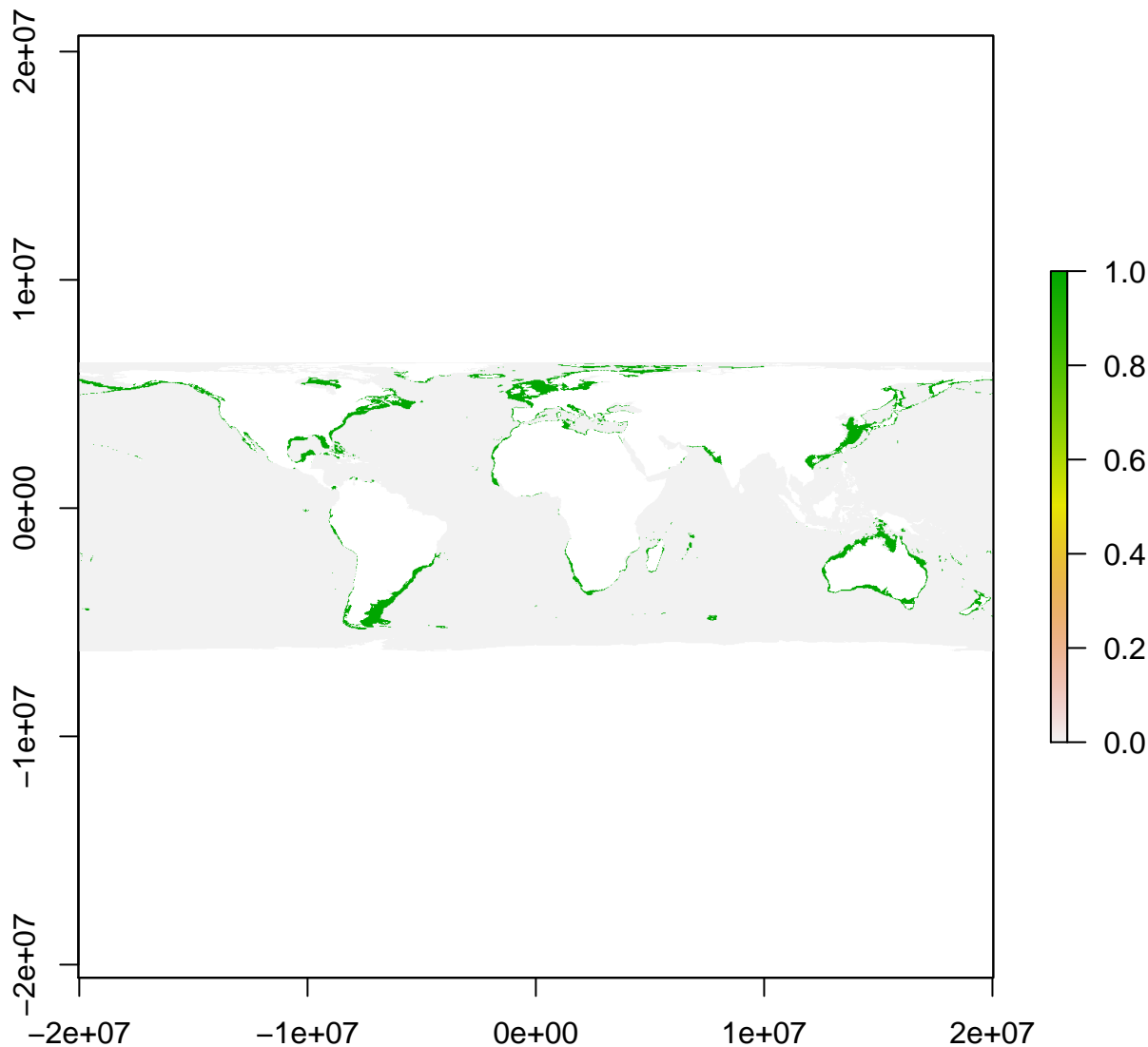

Supplement: Supplementary file 1 [file toxins-15-00009-s001.zip › toxins-2099991-supplementary/Supplementary Material/Projections/Binary/seriata/2050_RCP2.6_binary.pdf]

**Pseudo\_nitzchia\_serjata\_2050\_RCP4.5\_binary**

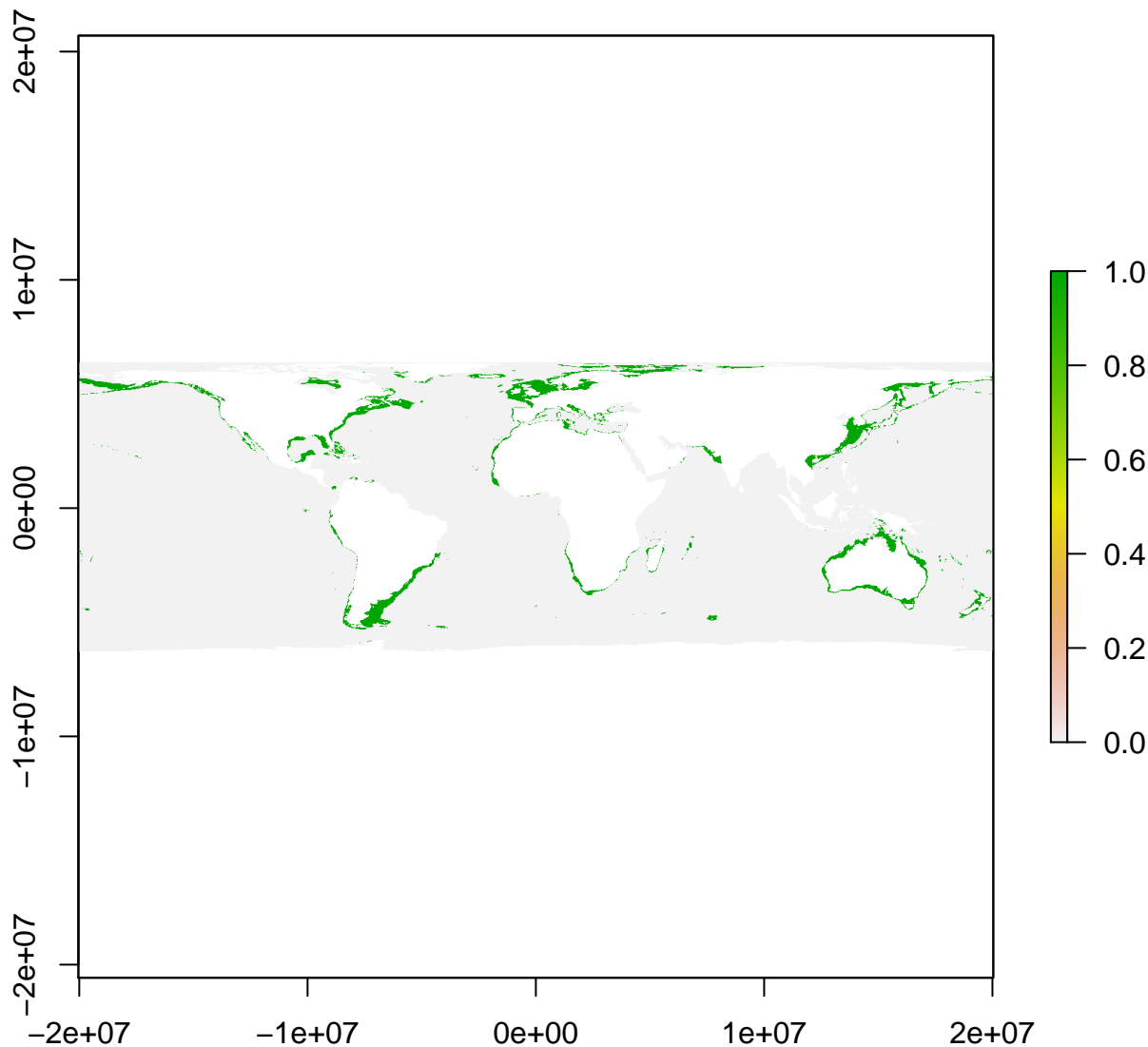

Supplement: Supplementary file 1 [file toxins-15-00009-s001.zip › toxins-2099991-supplementary/Supplementary Material/Projections/Binary/seriata/2050_RCP4.5_binary.pdf]

# Pseudo\_nitzchia\_serjata\_2050\_RCP6.0\_binary

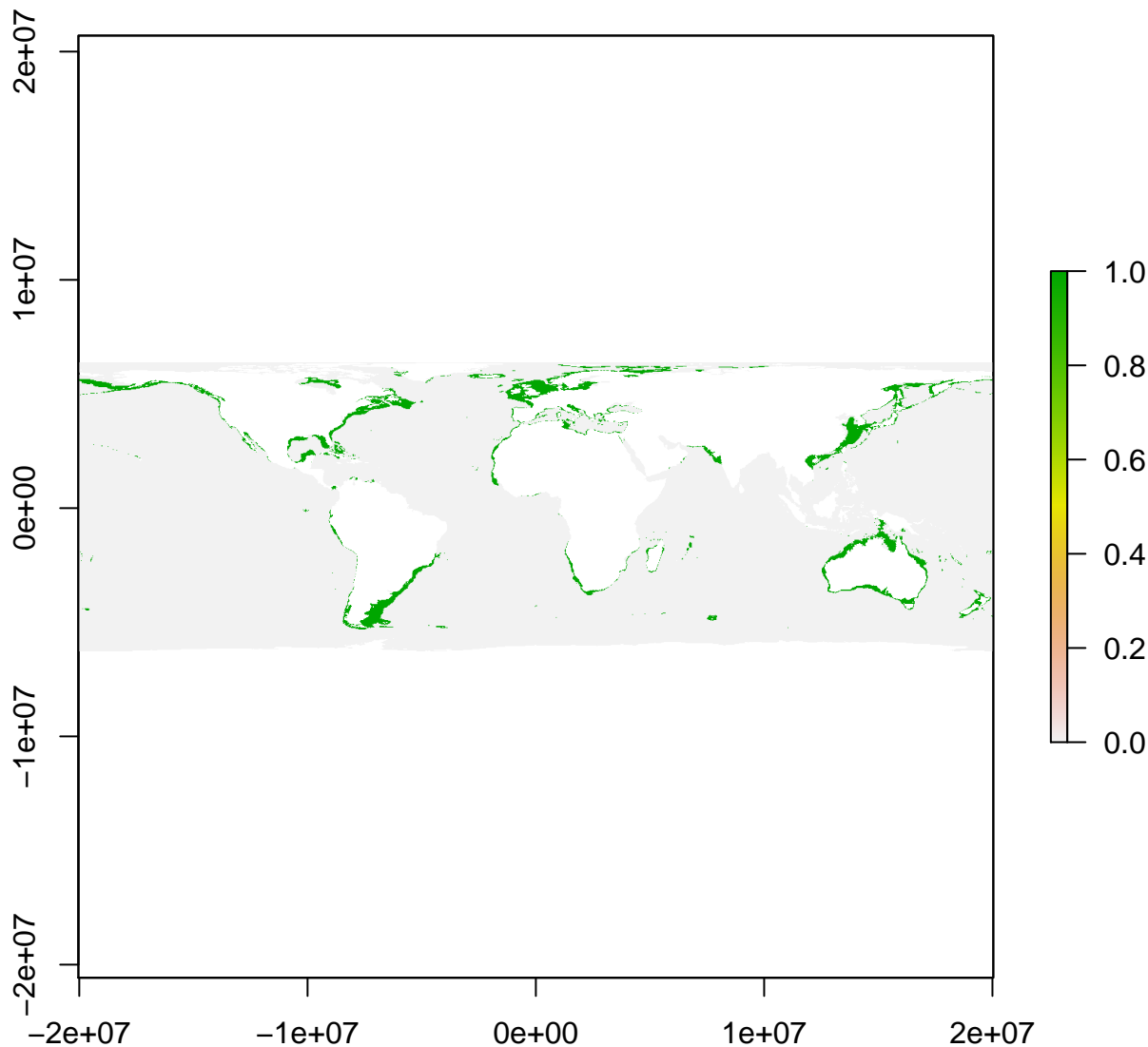

Supplement: Supplementary file 1 [file toxins-15-00009-s001.zip › toxins-2099991-supplementary/Supplementary Material/Projections/Binary/seriata/2050_RCP6.0_binary.pdf]

# Pseudo\_nitzchia\_serjata\_2050\_RCP8.5\_binary

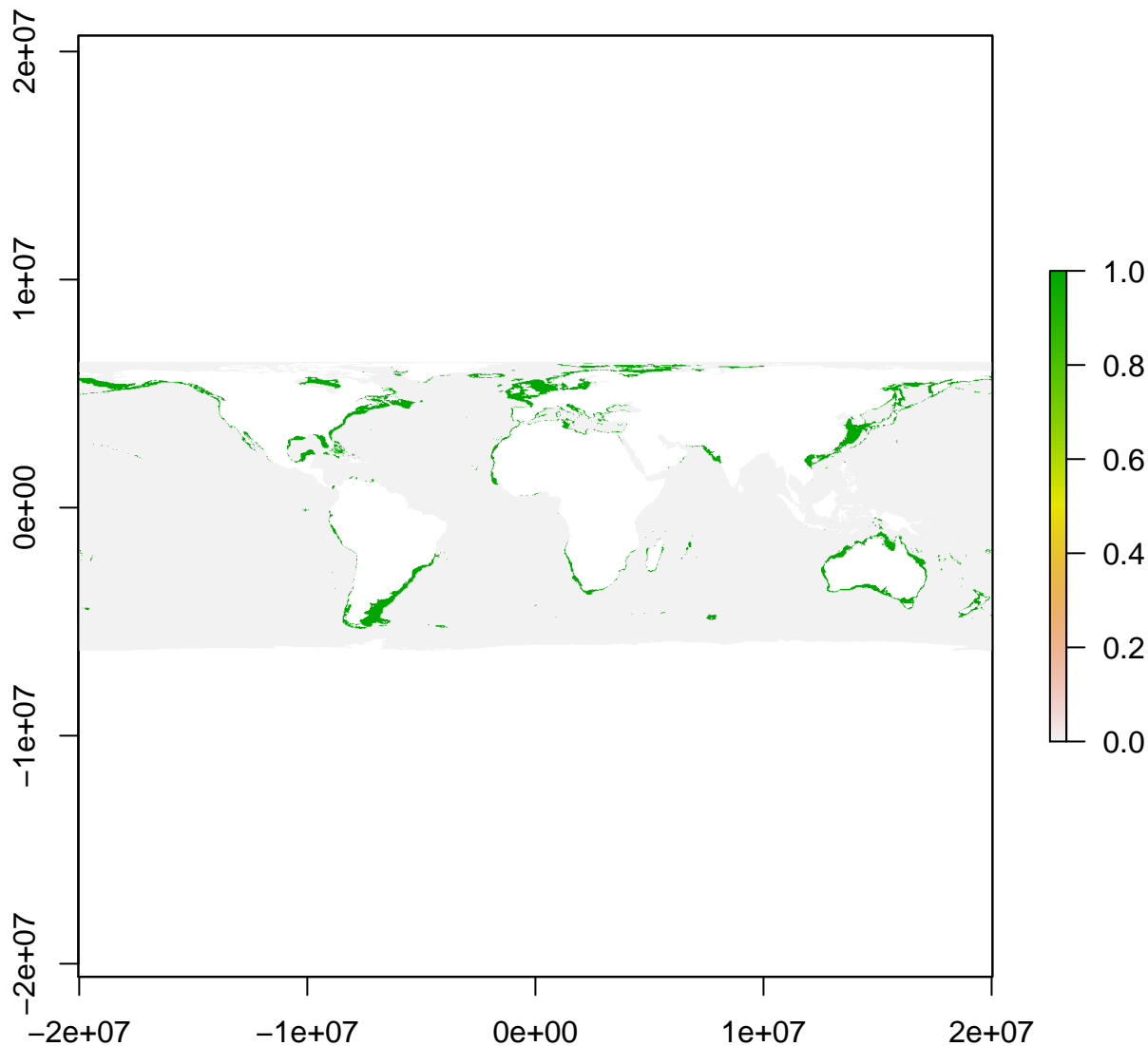

Supplement: Supplementary file 1 [file toxins-15-00009-s001.zip › toxins-2099991-supplementary/Supplementary Material/Projections/Binary/seriata/2050_RCP8.5_binary.pdf]

# Pseudo\_nitzchia\_serjata\_2100\_RCP2.6\_binary

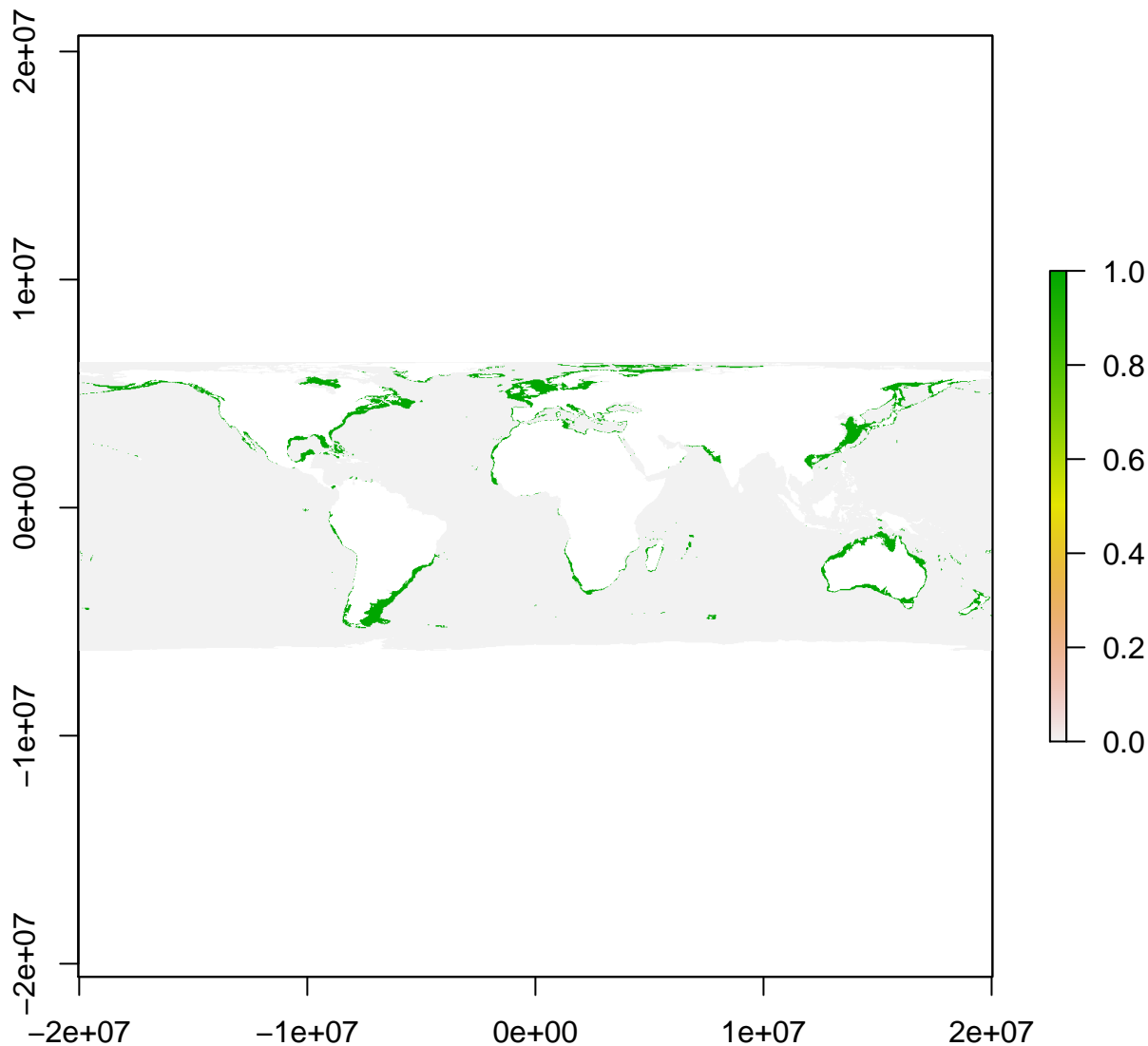

Supplement: Supplementary file 1 [file toxins-15-00009-s001.zip › toxins-2099991-supplementary/Supplementary Material/Projections/Binary/seriata/2100_RCP2.6_binary.pdf]

# Pseudo\_nitzchia\_serjata\_2100\_RCP4.5\_binary

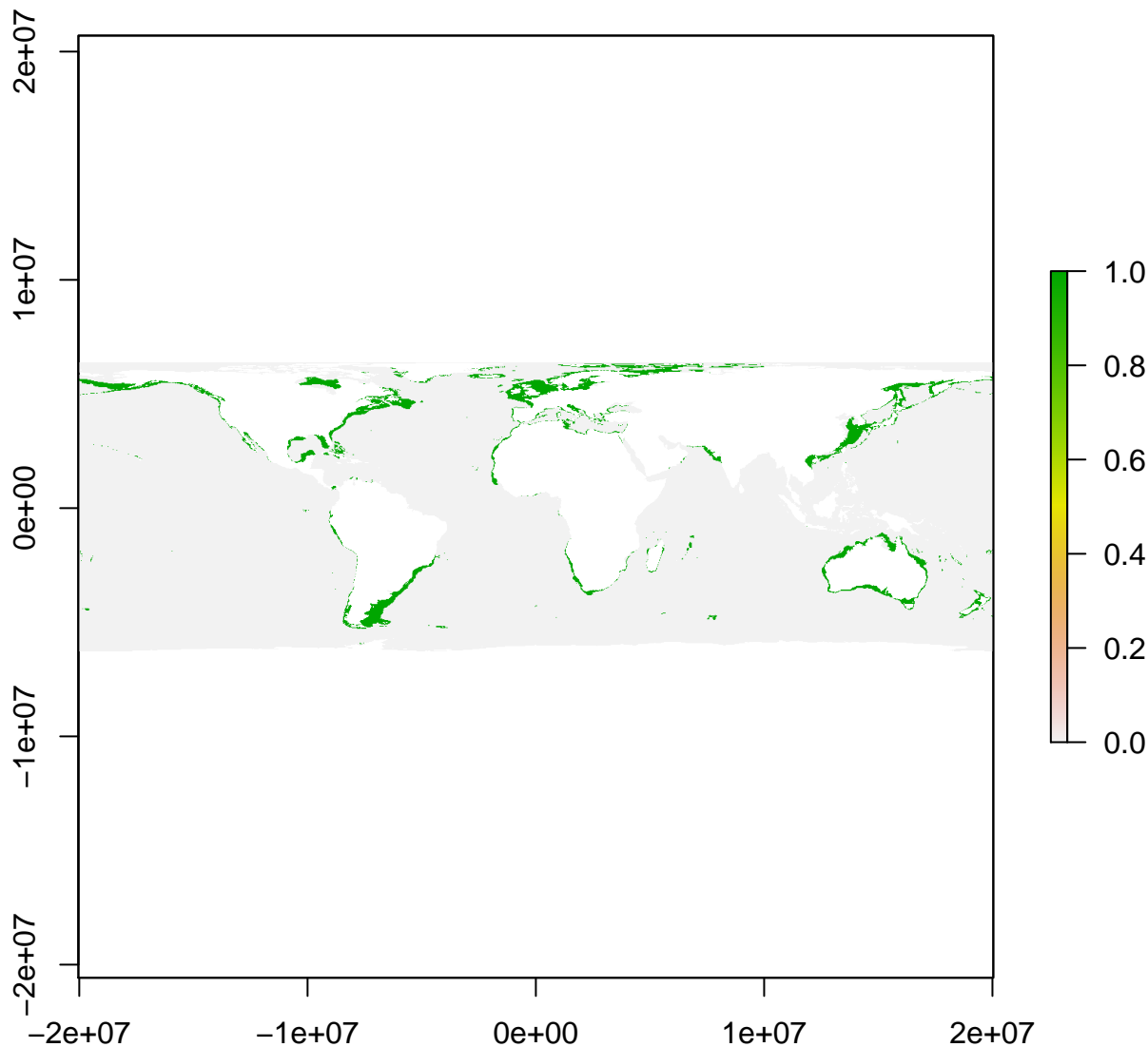

Supplement: Supplementary file 1 [file toxins-15-00009-s001.zip › toxins-2099991-supplementary/Supplementary Material/Projections/Binary/seriata/2100_RCP4.5_binary.pdf]

# Pseudo\_nitzchia\_serjata\_2100\_RCP6.0\_binary

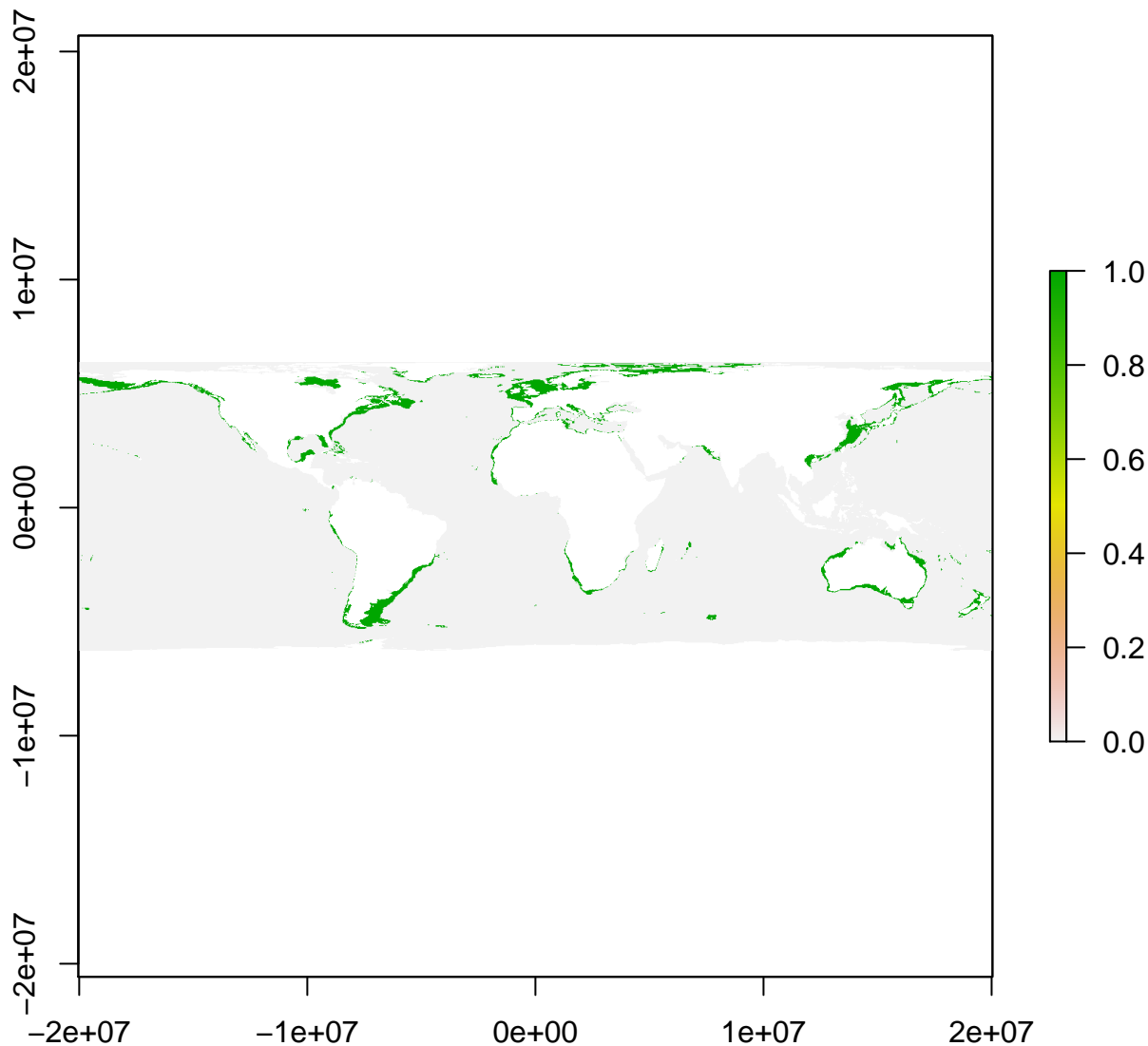

Supplement: Supplementary file 1 [file toxins-15-00009-s001.zip › toxins-2099991-supplementary/Supplementary Material/Projections/Binary/seriata/2100_RCP6.0_binary.pdf]

# Pseudo\_nitzchia\_serjata\_2100\_RCP8.5\_binary

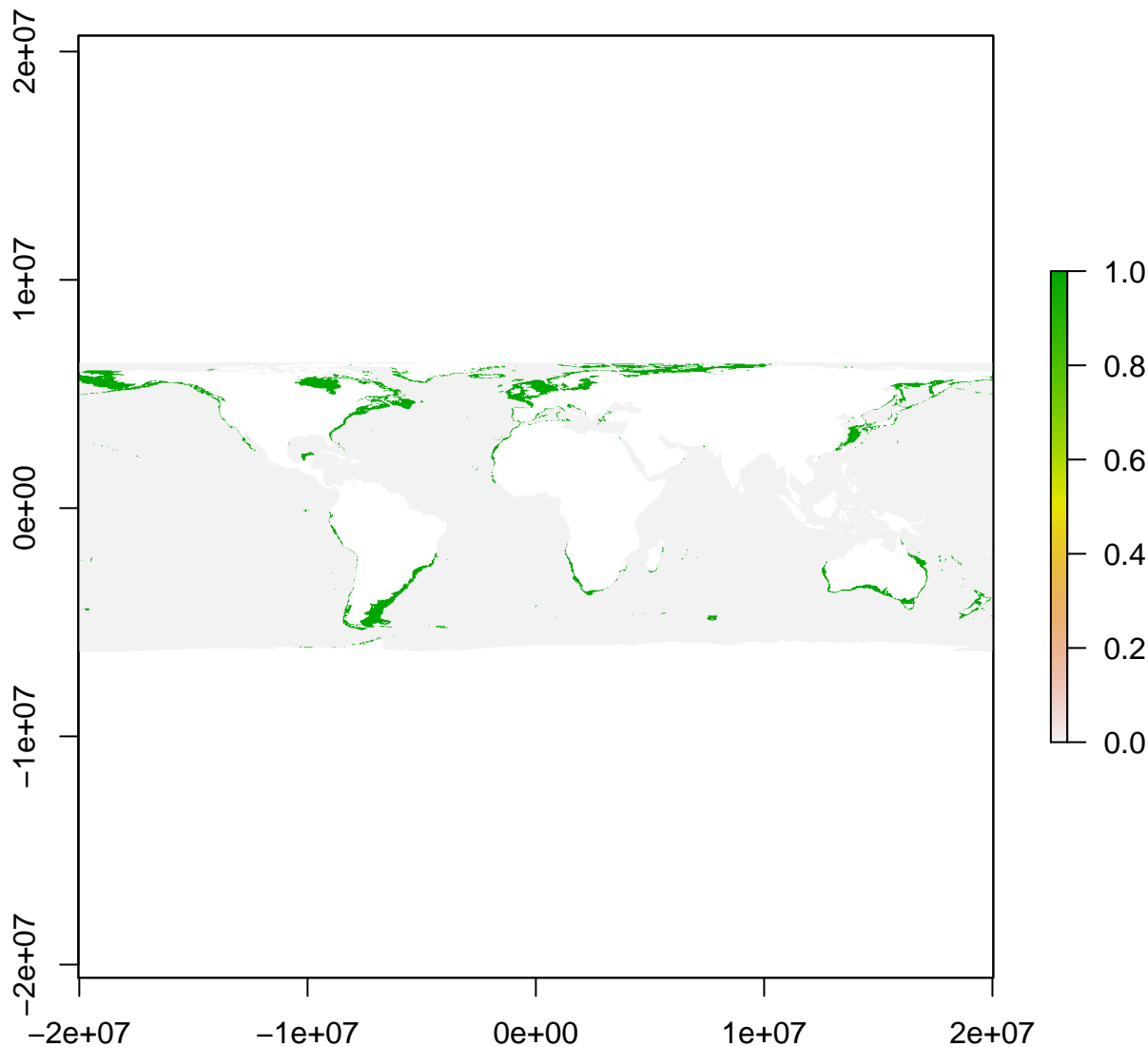

Supplement: Supplementary file 1 [file toxins-15-00009-s001.zip › toxins-2099991-supplementary/Supplementary Material/Projections/Binary/seriata/2100_RCP8.5_binary.pdf]

# Pseudo\_nitzchia\_serziata\_present\_binary

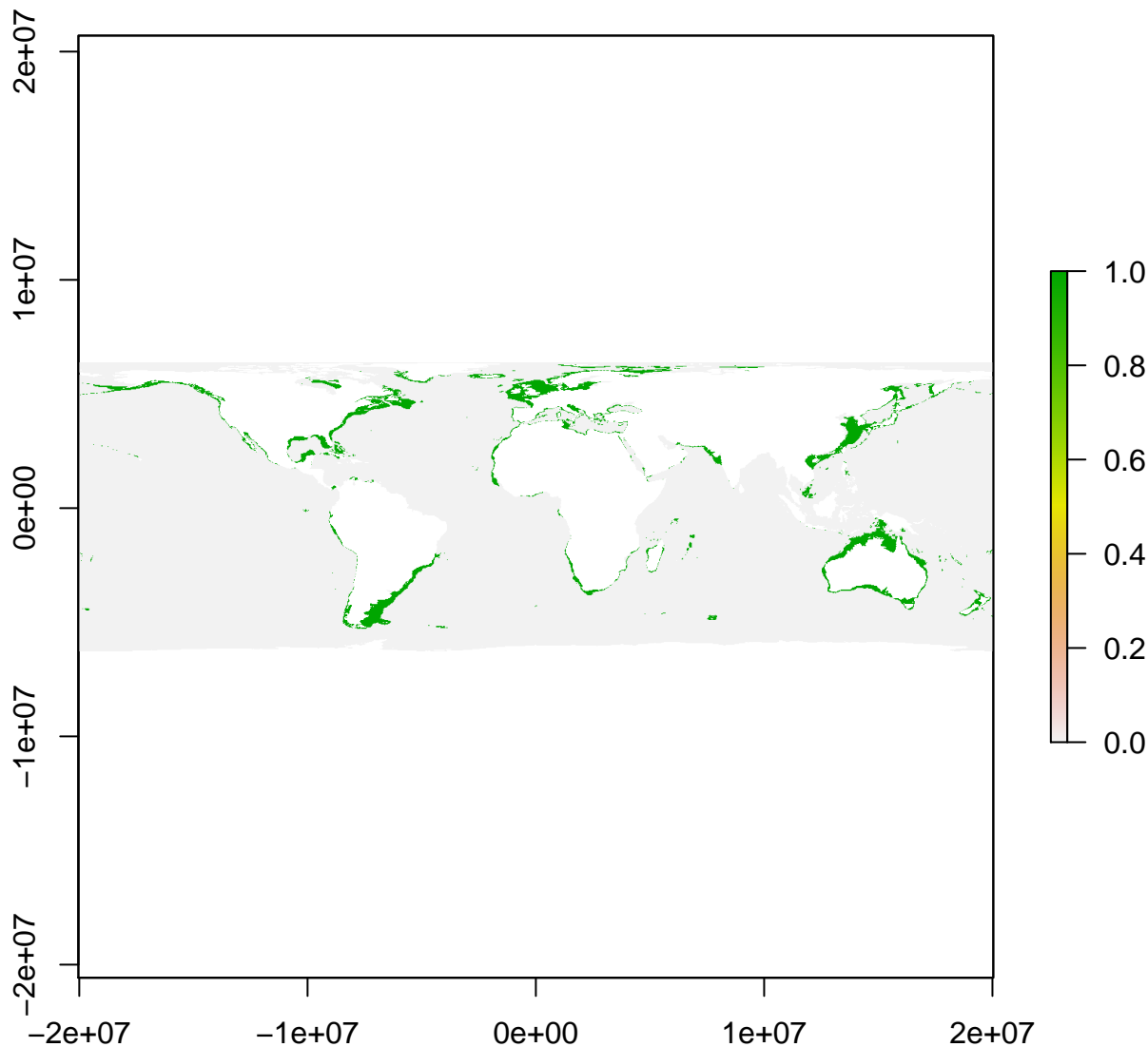

Supplement: Supplementary file 1 [file toxins-15-00009-s001.zip › toxins-2099991-supplementary/Supplementary Material/Projections/Binary/seriata/present_binary.pdf]

# Pseudo-nitzschia\_australis\_2050\_RCP2.6

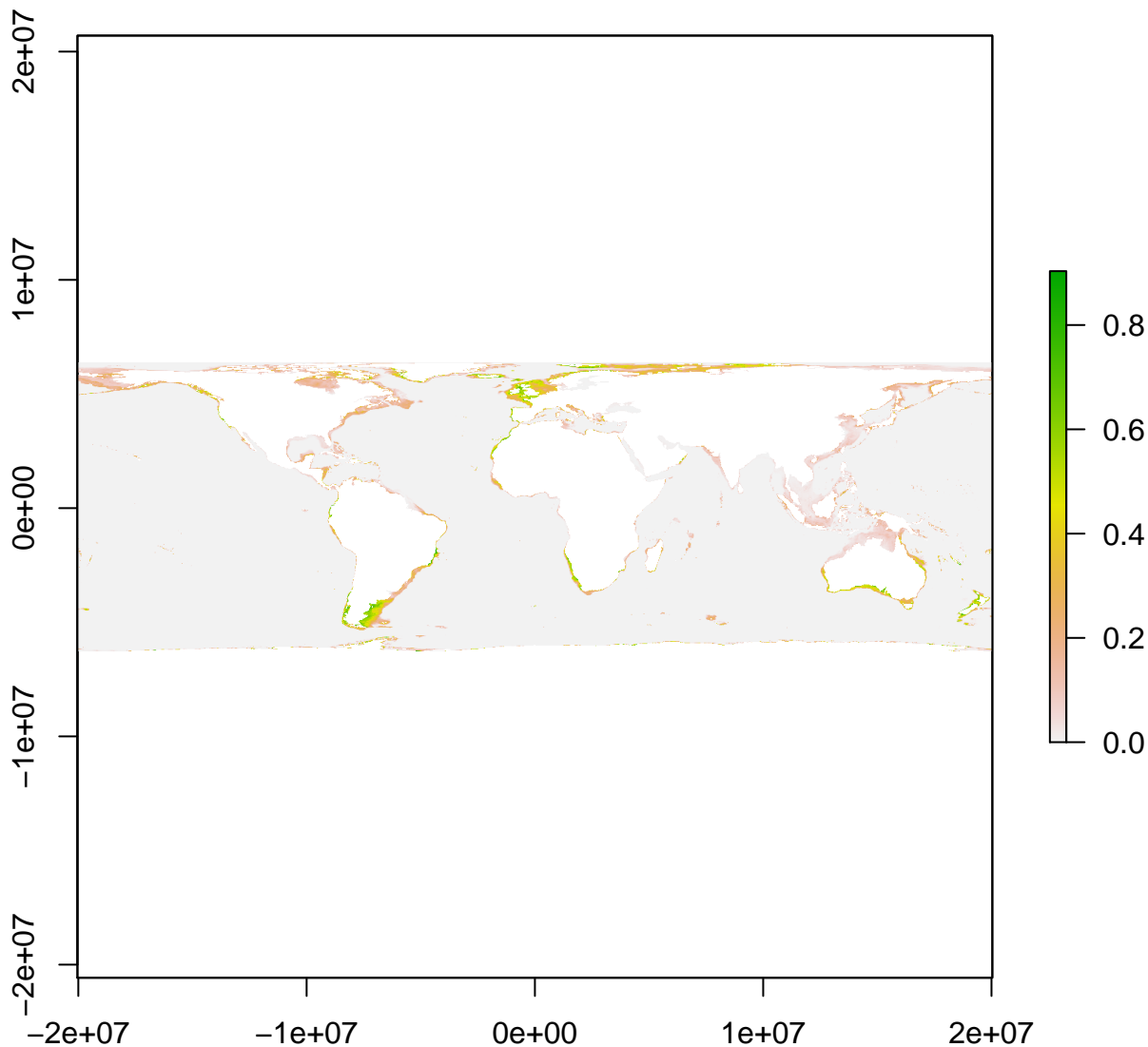

Supplement: Supplementary file 1 [file toxins-15-00009-s001.zip › toxins-2099991-supplementary/Supplementary Material/Projections/Ensemble/australis/2050_RCP2.6_ensembled.pdf]

# Pseudo-nitzschia\_australis\_2050\_RCP4.5

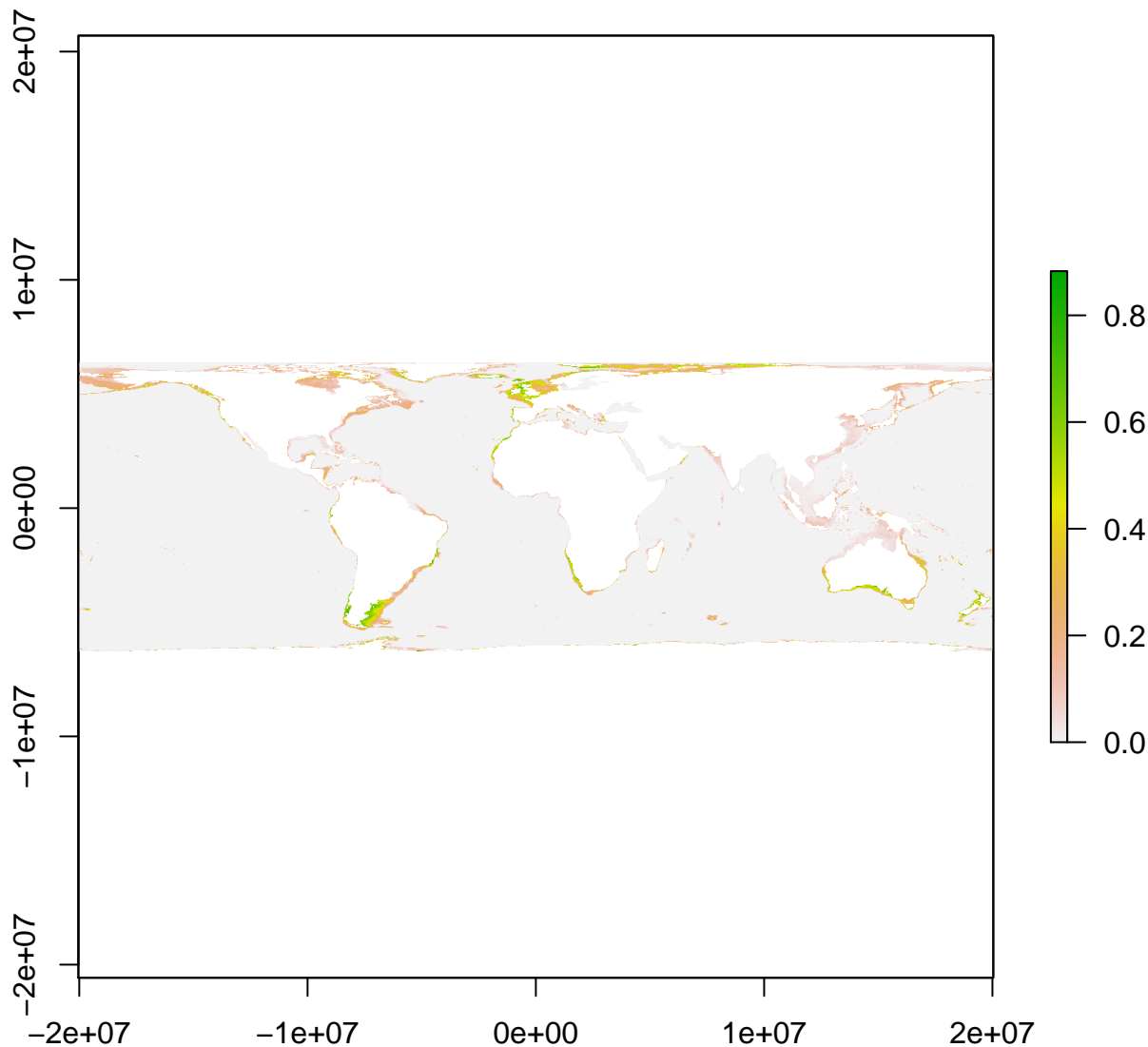

Supplement: Supplementary file 1 [file toxins-15-00009-s001.zip › toxins-2099991-supplementary/Supplementary Material/Projections/Ensemble/australis/2050_RCP4.5_ensembled.pdf]

# Pseudo-nitzschia\_australis\_2050\_RCP6.0

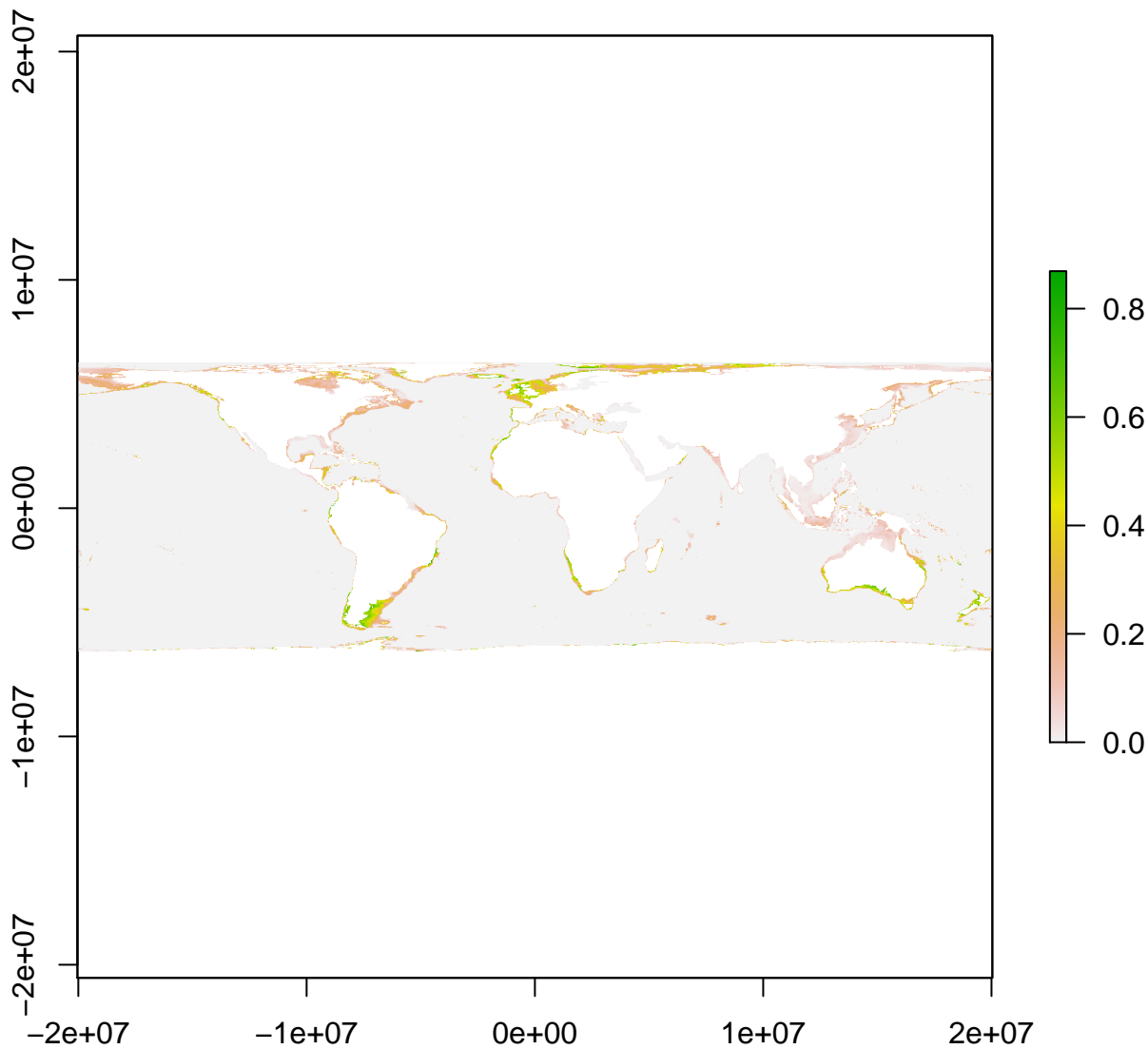

Supplement: Supplementary file 1 [file toxins-15-00009-s001.zip › toxins-2099991-supplementary/Supplementary Material/Projections/Ensemble/australis/2050_RCP6.0_ensembled.pdf]

# Pseudo-nitzschia\_australis\_2050\_RCP8.5

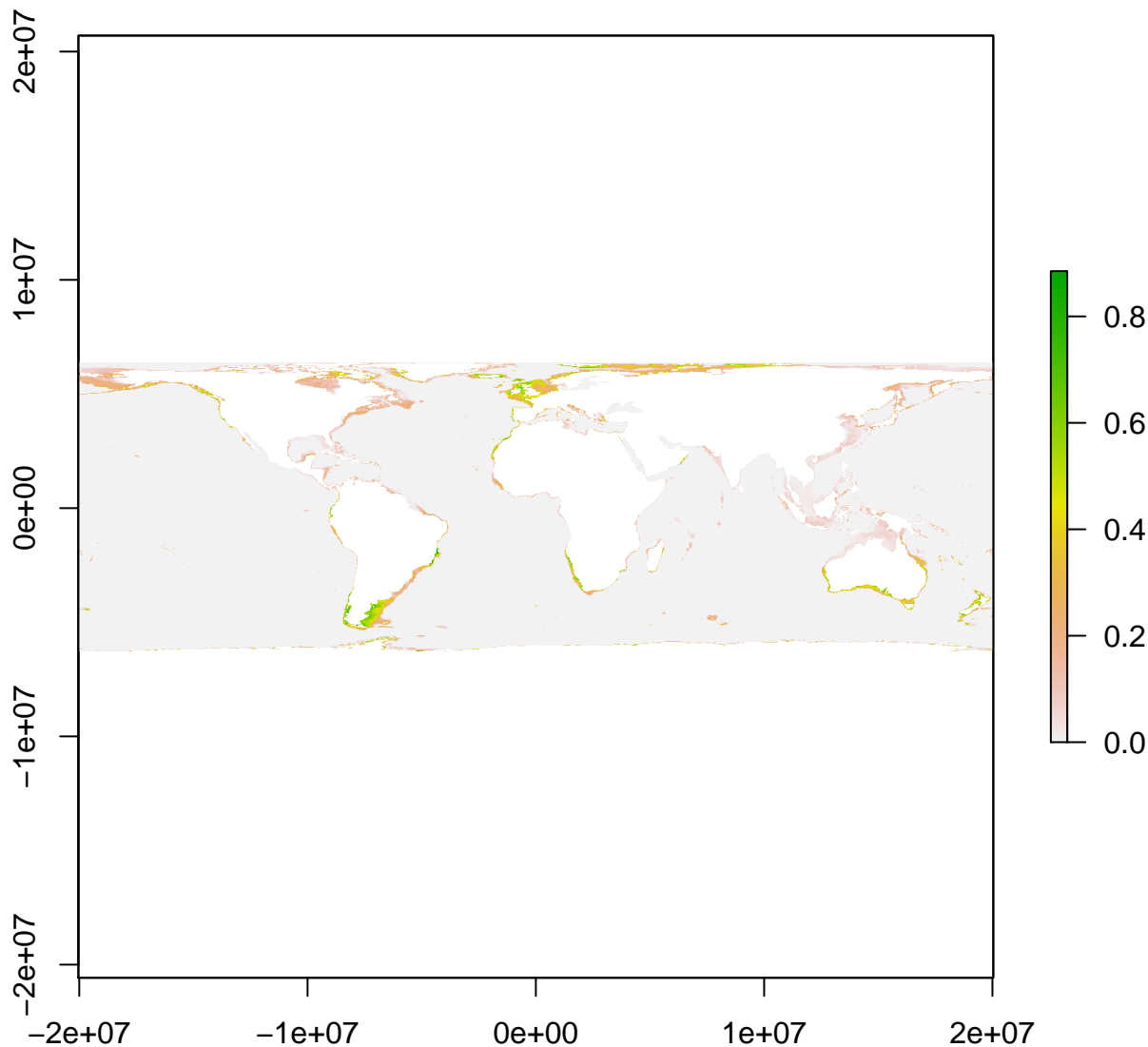

Supplement: Supplementary file 1 [file toxins-15-00009-s001.zip › toxins-2099991-supplementary/Supplementary Material/Projections/Ensemble/australis/2050_RCP8.5_ensembled.pdf]

# Pseudo-nitzschia\_australis\_2100\_RCP2.6

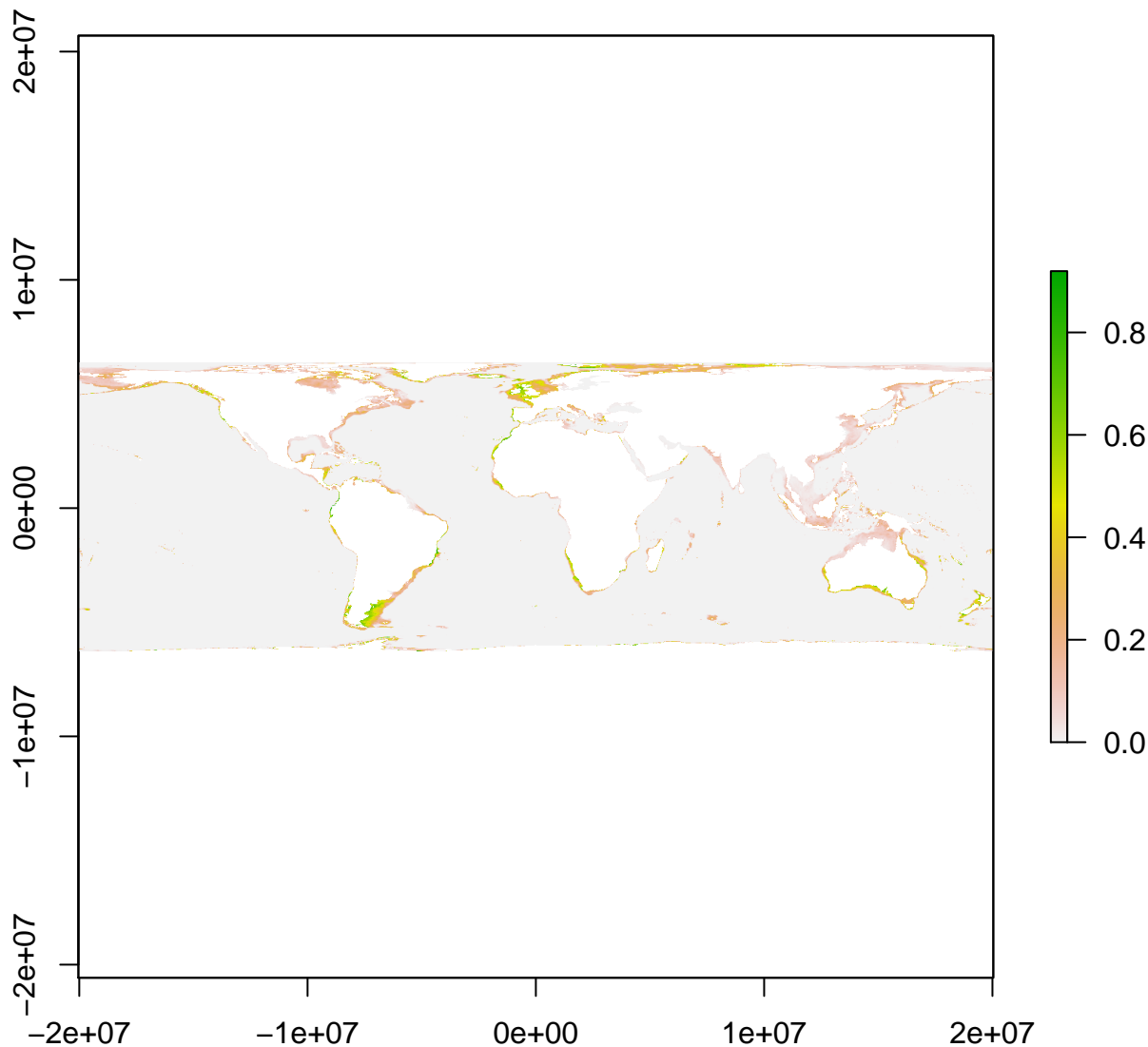

Supplement: Supplementary file 1 [file toxins-15-00009-s001.zip › toxins-2099991-supplementary/Supplementary Material/Projections/Ensemble/australis/2100_RCP2.6_ensembled.pdf]

# Pseudo-nitzschia\_australis\_2100\_RCP4.5

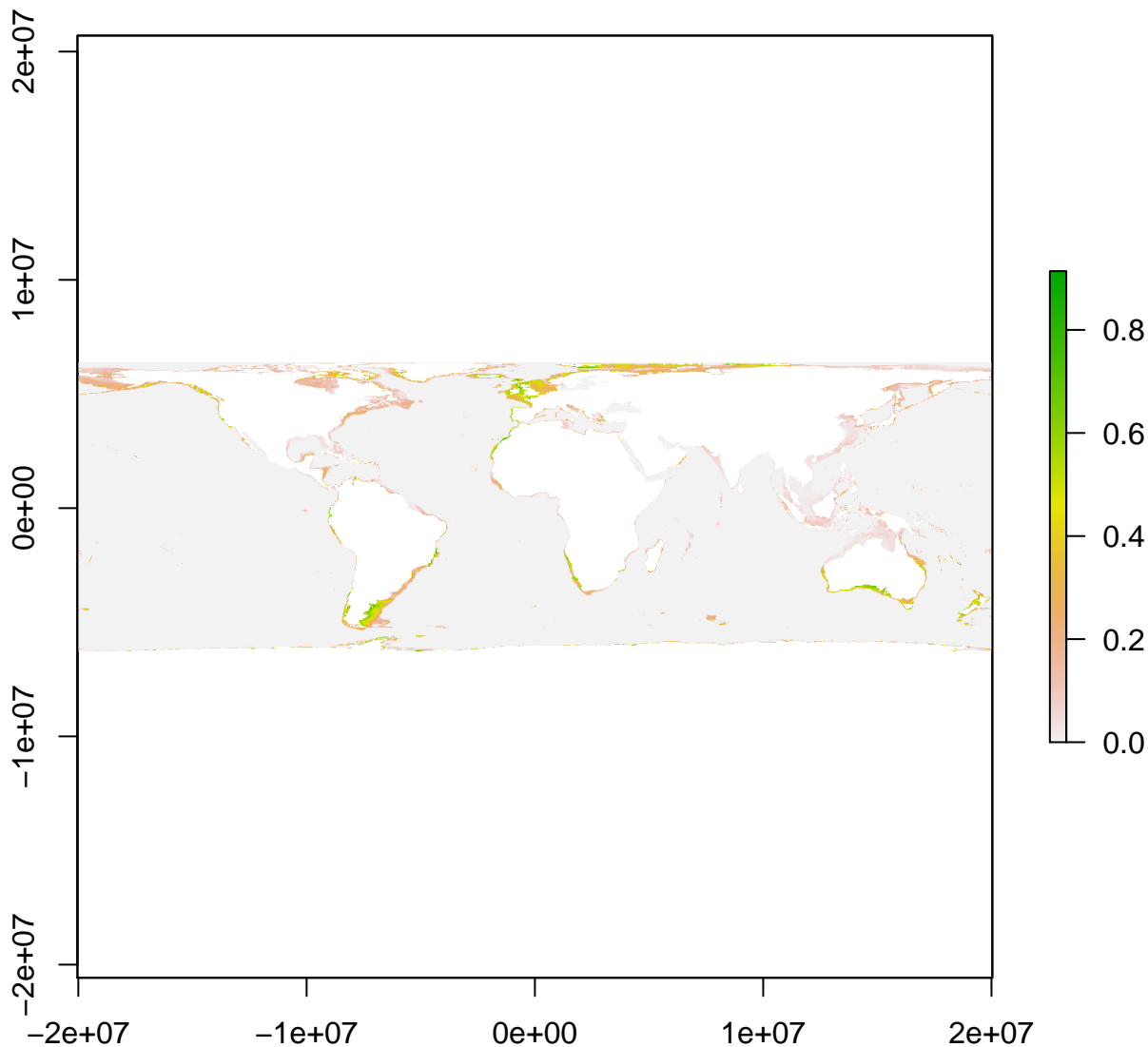

Supplement: Supplementary file 1 [file toxins-15-00009-s001.zip › toxins-2099991-supplementary/Supplementary Material/Projections/Ensemble/australis/2100_RCP4.5_ensembled.pdf]

# Pseudo-nitzschia\_australis\_2100\_RCP6.0

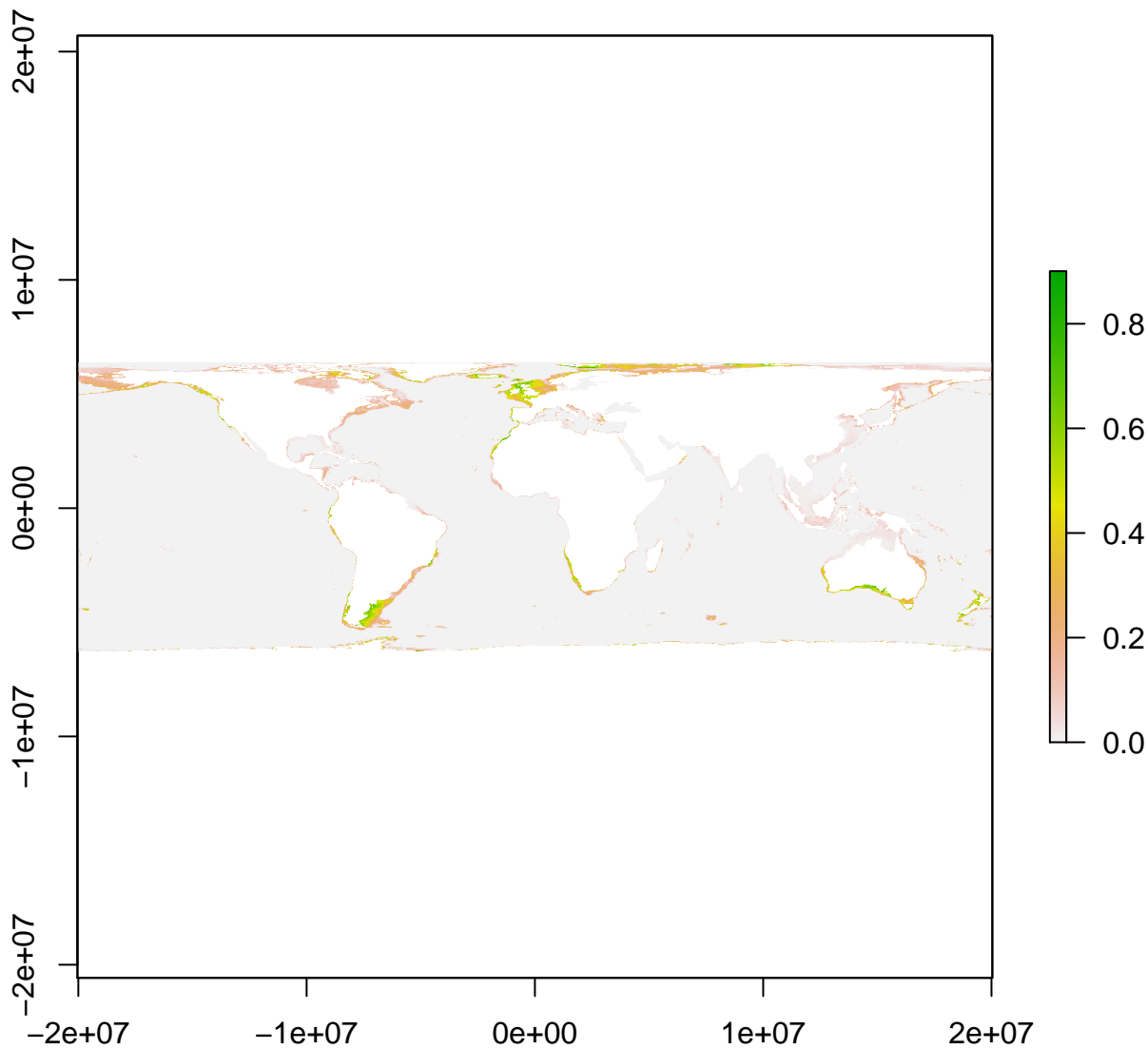

Supplement: Supplementary file 1 [file toxins-15-00009-s001.zip › toxins-2099991-supplementary/Supplementary Material/Projections/Ensemble/australis/2100_RCP6.0_ensembled.pdf]

# Pseudo-nitzschia\_australis\_2100\_RCP8.5

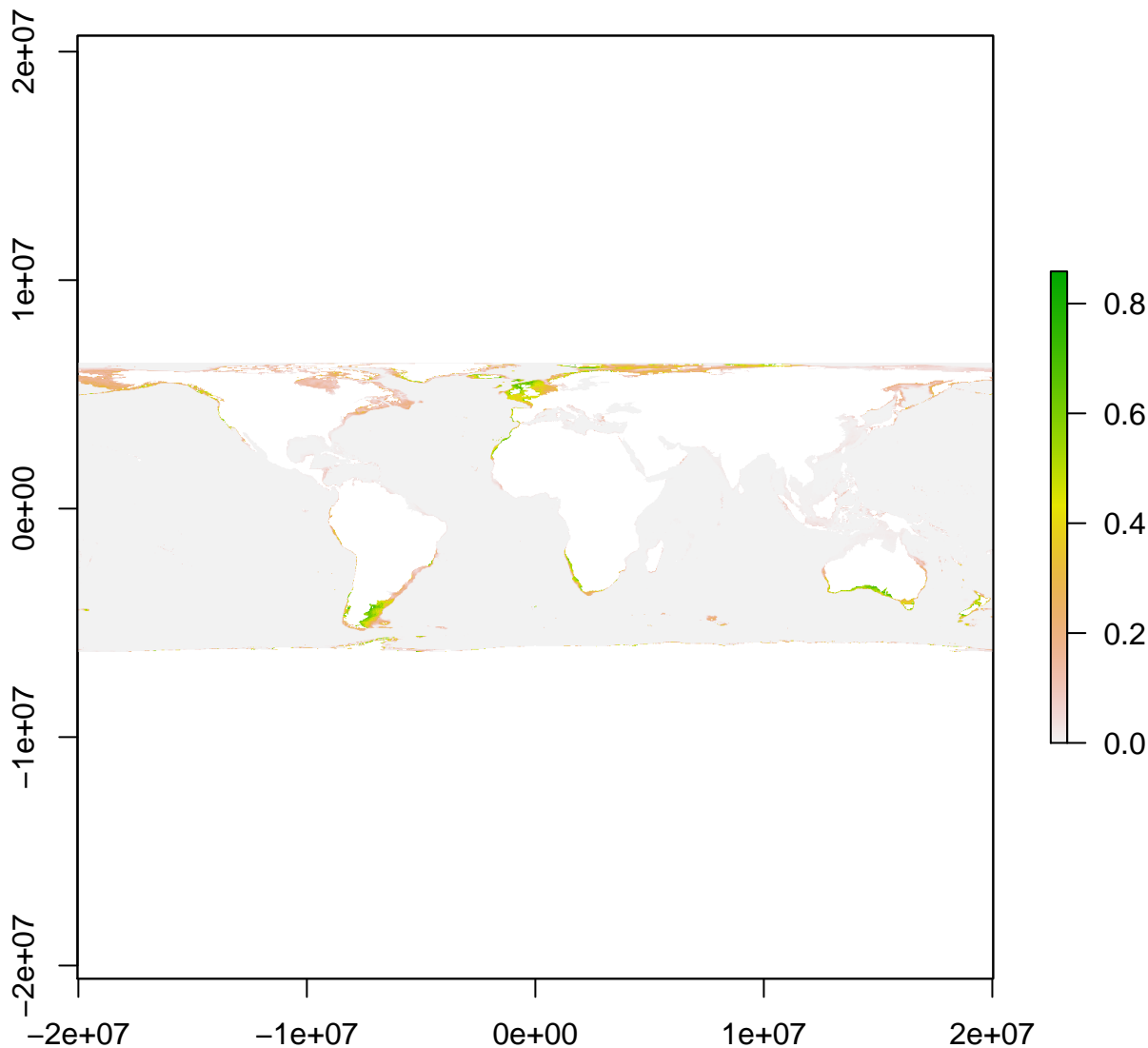

Supplement: Supplementary file 1 [file toxins-15-00009-s001.zip › toxins-2099991-supplementary/Supplementary Material/Projections/Ensemble/australis/2100_RCP8.5_ensembled.pdf]

# Pseudo-nitzschia\_australis\_present

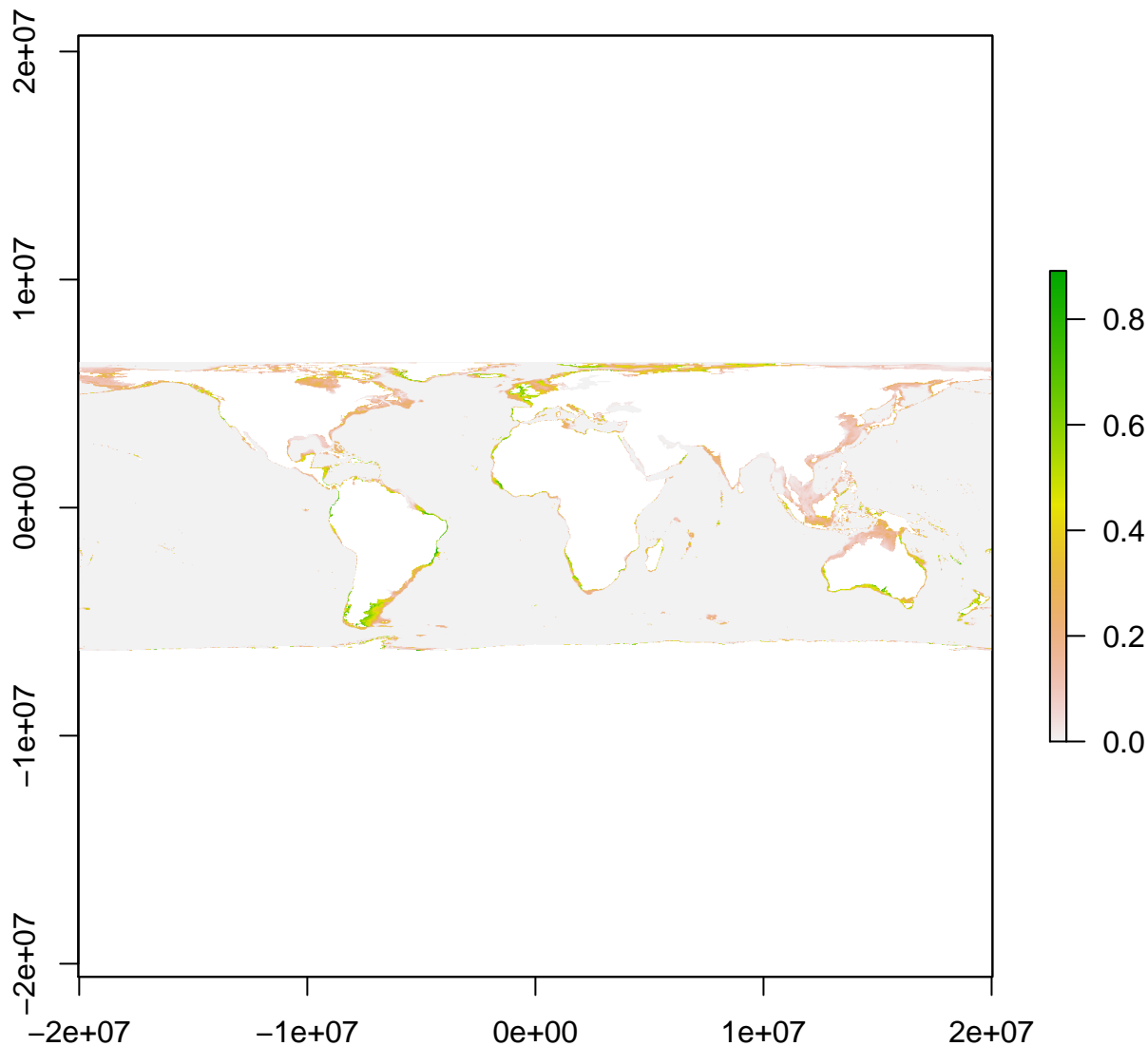

Supplement: Supplementary file 1 [file toxins-15-00009-s001.zip › toxins-2099991-supplementary/Supplementary Material/Projections/Ensemble/australis/present_ensembled.pdf]

# Pseudo\_nitzchia\_fraudulenta\_2050\_RCP2.6

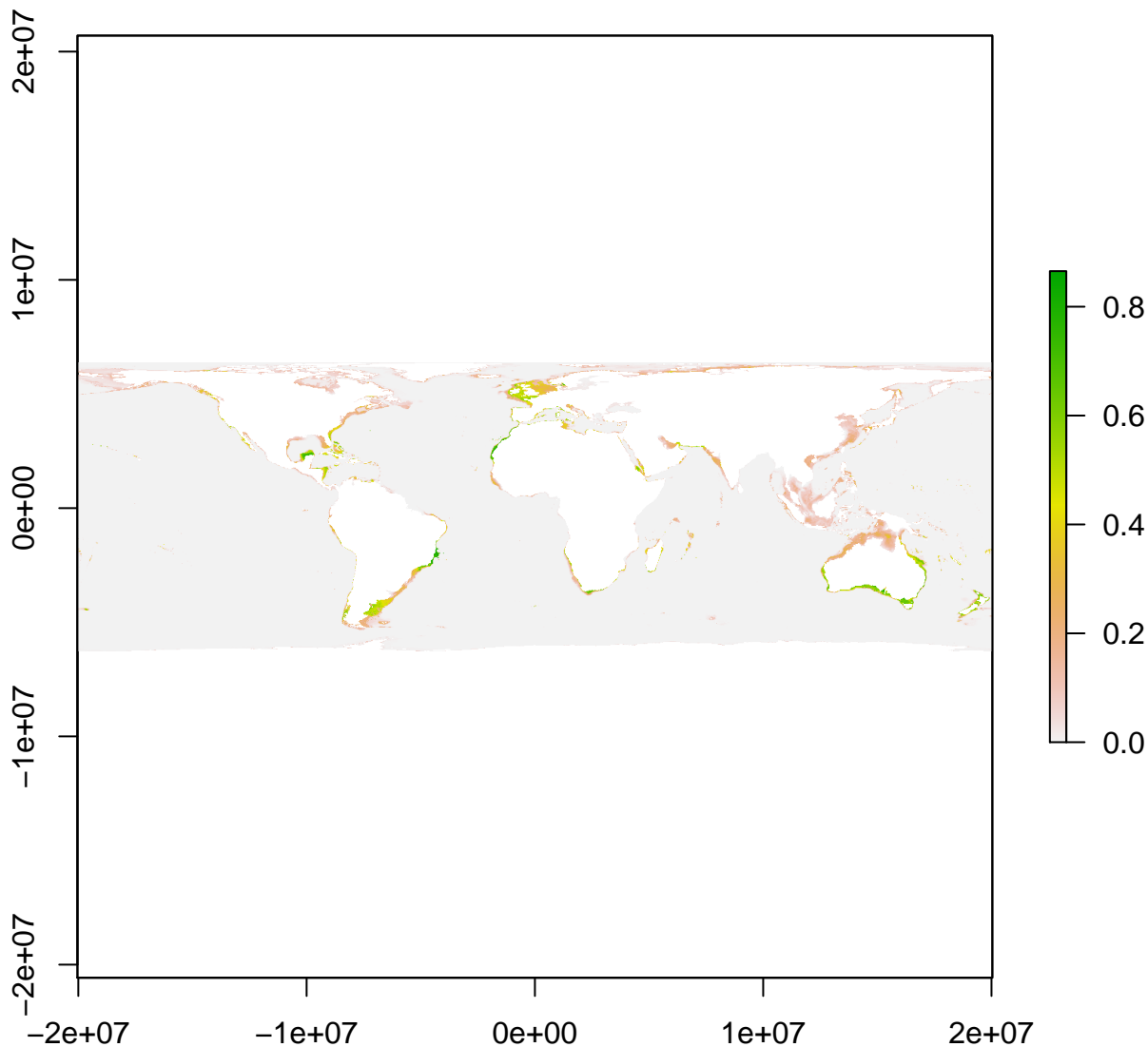

Supplement: Supplementary file 1 [file toxins-15-00009-s001.zip › toxins-2099991-supplementary/Supplementary Material/Projections/Ensemble/fraudulenta/2050_RCP2.6_ensembled.pdf]

# Pseudo\_nitzchia\_fraudulenta\_2050\_RCP4.5

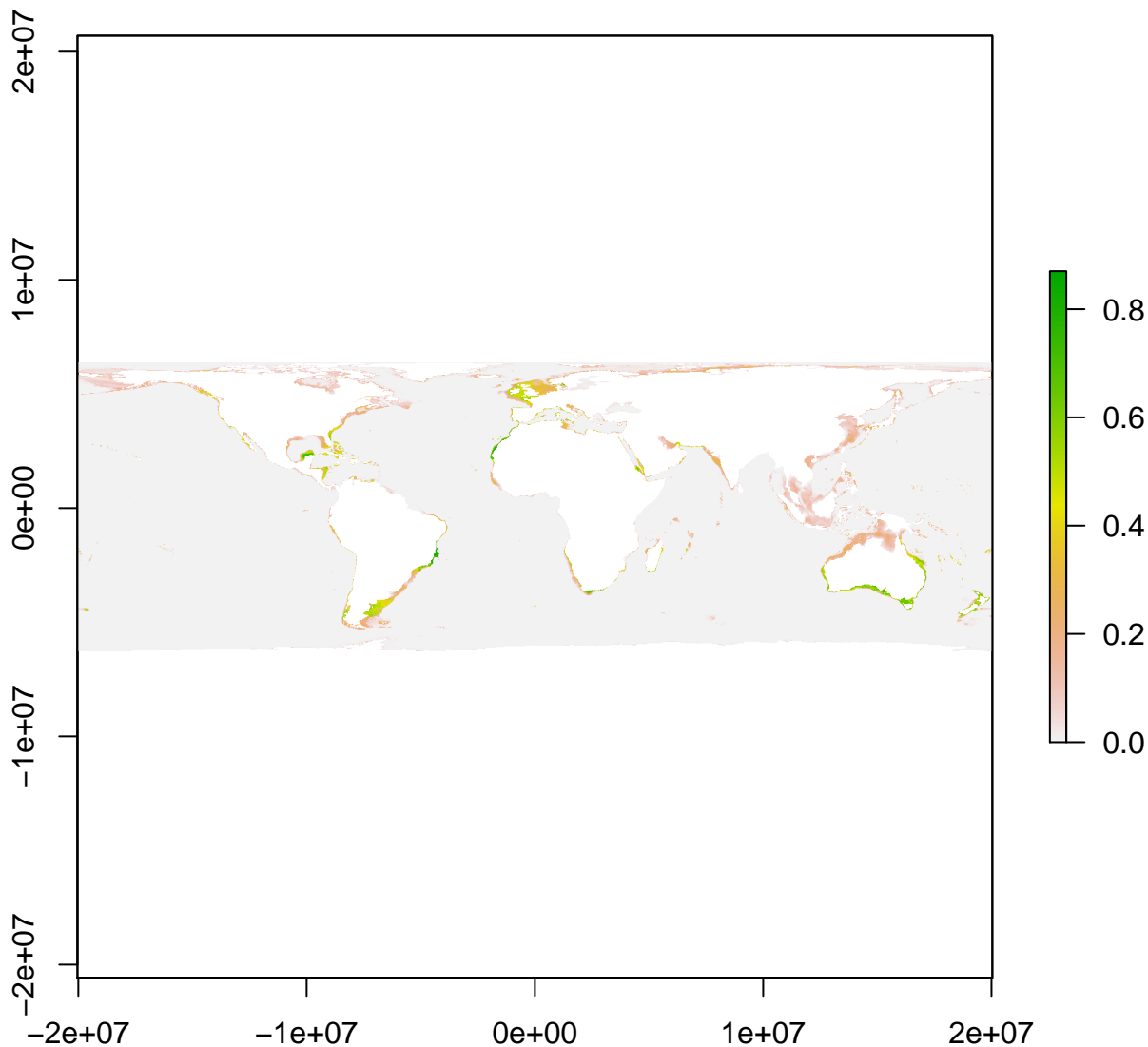

Supplement: Supplementary file 1 [file toxins-15-00009-s001.zip › toxins-2099991-supplementary/Supplementary Material/Projections/Ensemble/fraudulenta/2050_RCP4.5_ensembled.pdf]

# Pseudo\_nitzchia\_fraudulenta\_2050\_RCP6.0

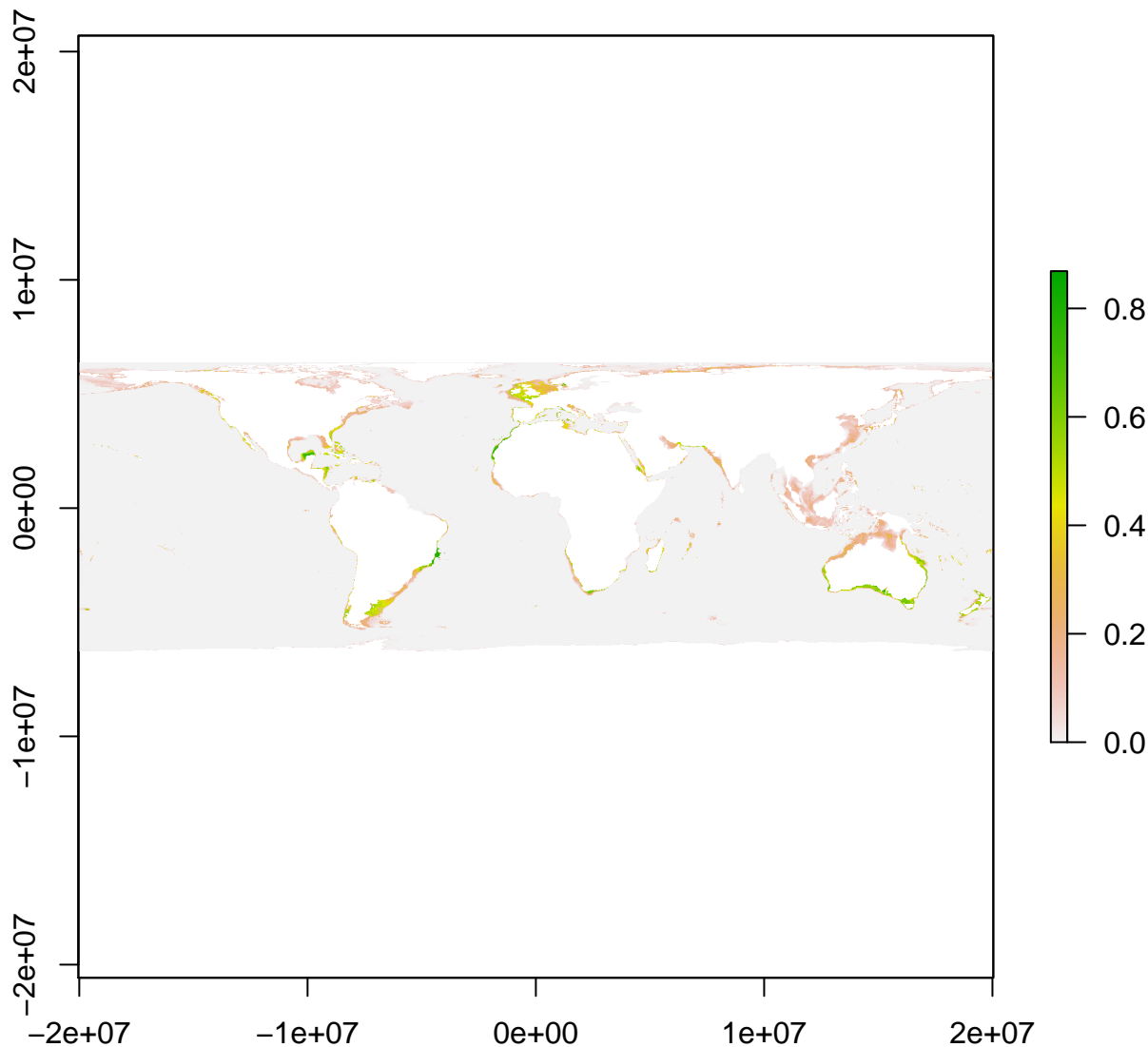

Supplement: Supplementary file 1 [file toxins-15-00009-s001.zip › toxins-2099991-supplementary/Supplementary Material/Projections/Ensemble/fraudulenta/2050_RCP6.0_ensembled.pdf]

# Pseudo\_nitzchia\_fraudulenta\_2050\_RCP8.5

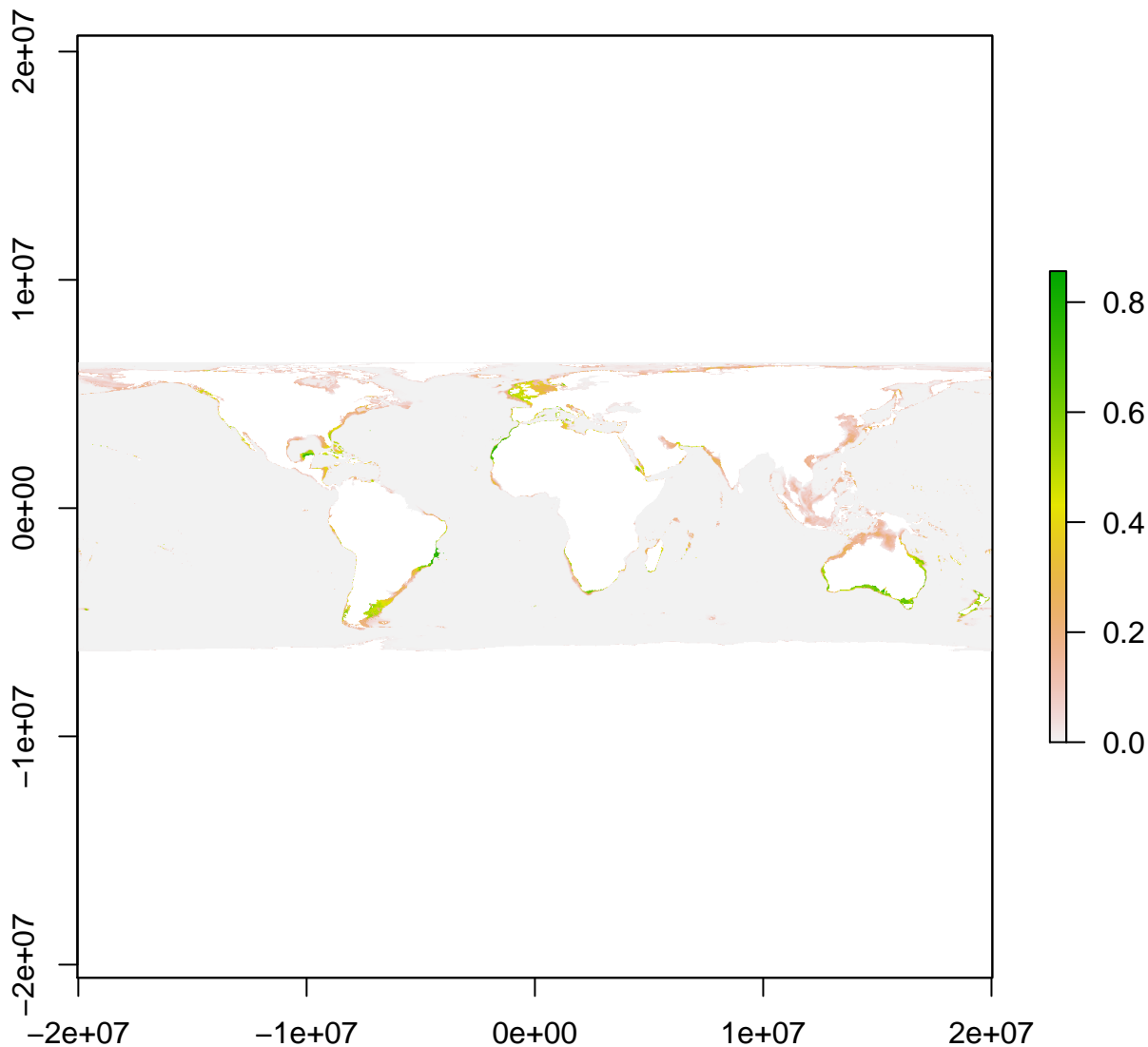

Supplement: Supplementary file 1 [file toxins-15-00009-s001.zip › toxins-2099991-supplementary/Supplementary Material/Projections/Ensemble/fraudulenta/2050_RCP8.5_ensembled.pdf]

# Pseudo\_nitzchia\_fraudulenta\_2100\_RCP2.6

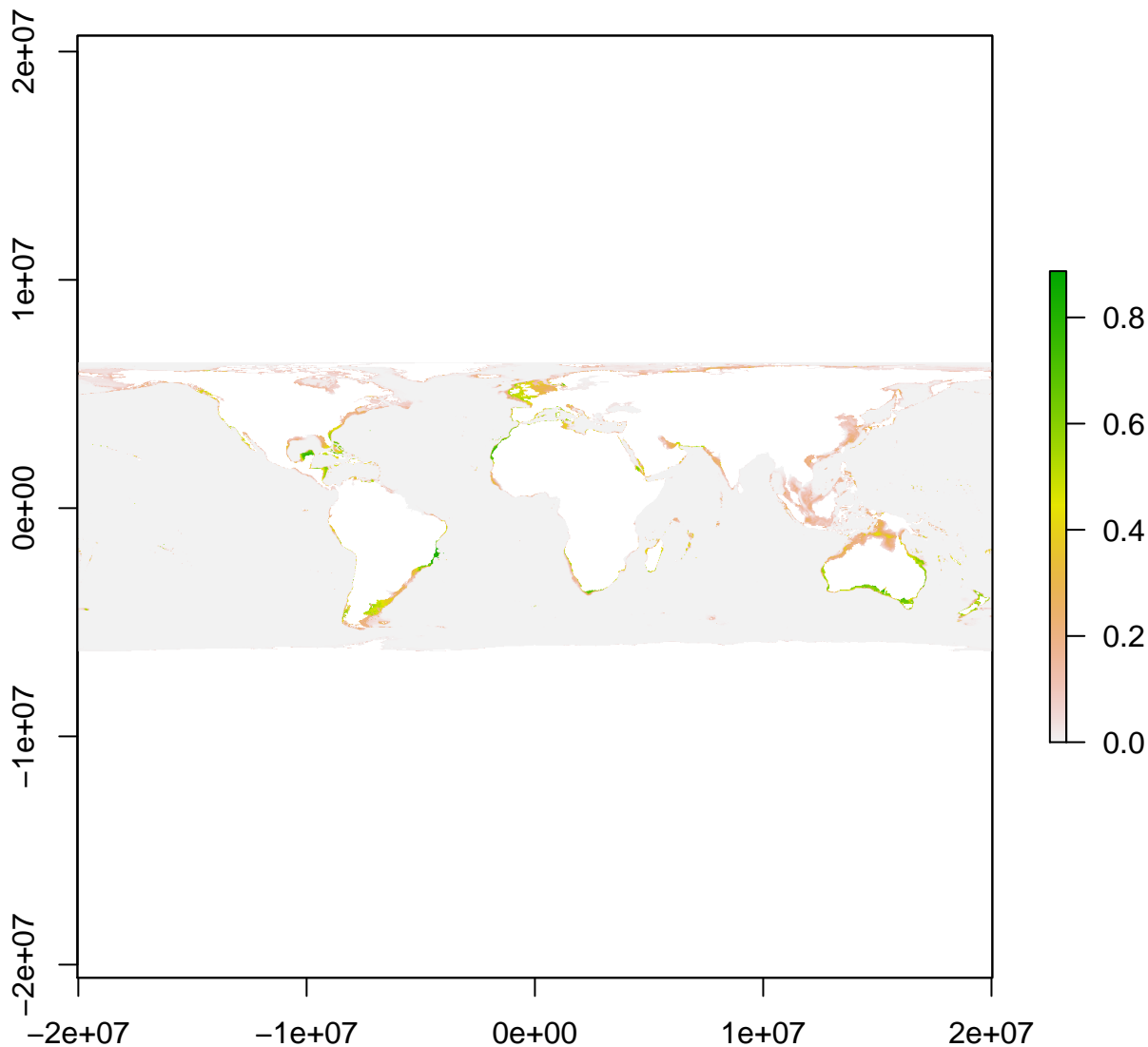

Supplement: Supplementary file 1 [file toxins-15-00009-s001.zip › toxins-2099991-supplementary/Supplementary Material/Projections/Ensemble/fraudulenta/2100_RCP2.6_ensembled.pdf]

# Pseudo\_nitzchia\_fraudulenta\_2100\_RCP4.5

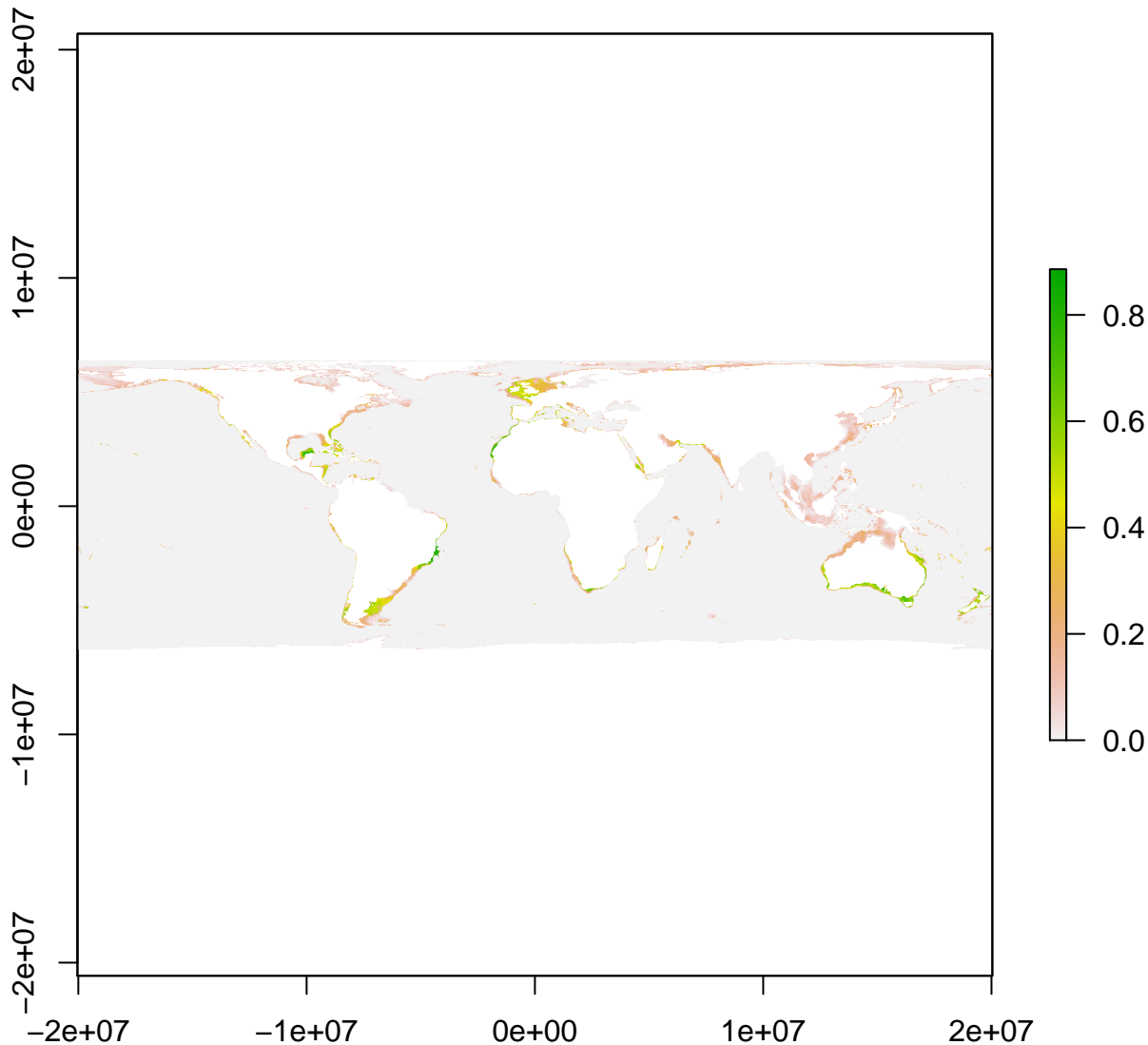

Supplement: Supplementary file 1 [file toxins-15-00009-s001.zip › toxins-2099991-supplementary/Supplementary Material/Projections/Ensemble/fraudulenta/2100_RCP4.5_ensembled.pdf]

# Pseudo\_nitzchia\_fraudulenta\_2100\_RCP6.0

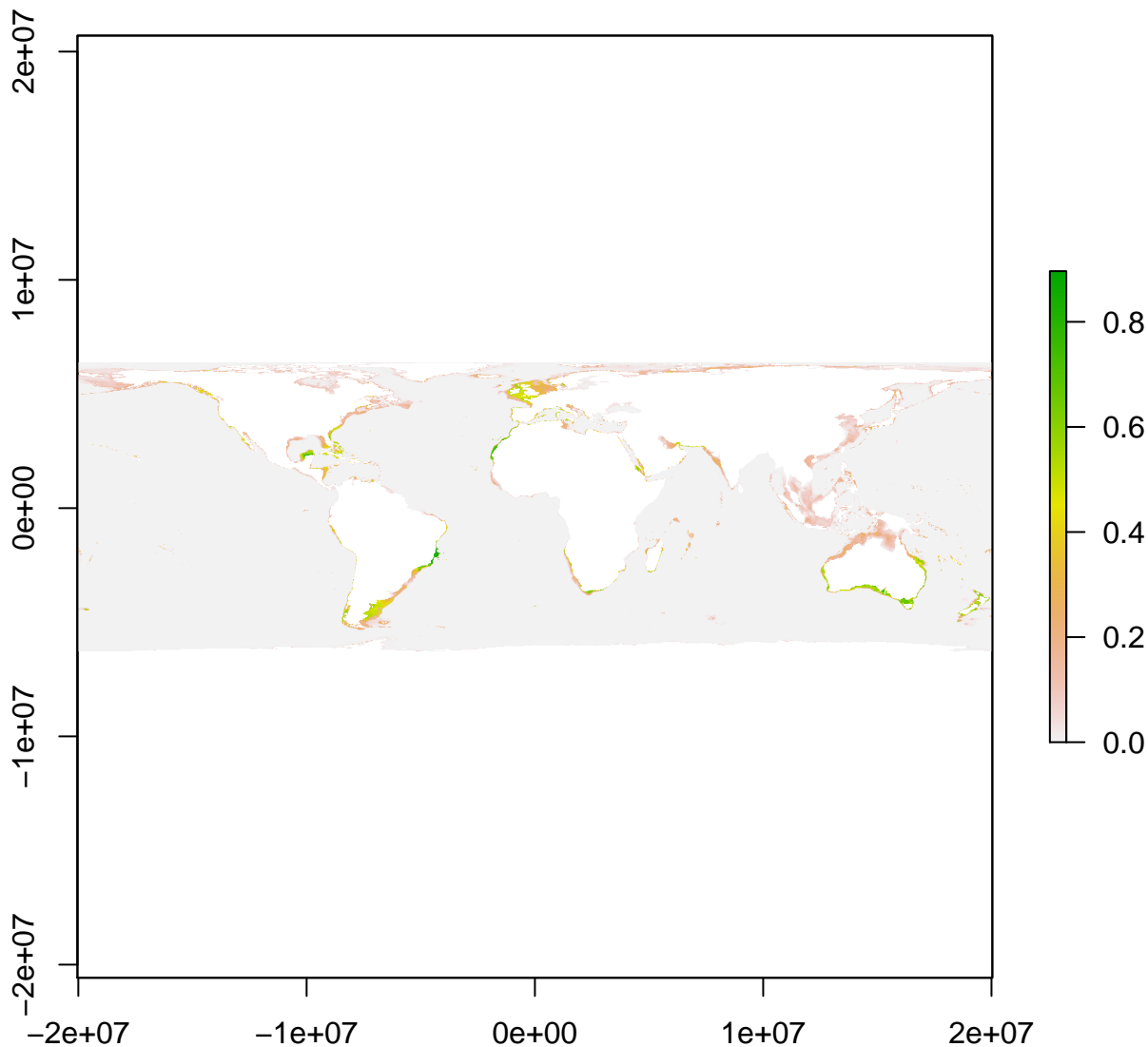

Supplement: Supplementary file 1 [file toxins-15-00009-s001.zip › toxins-2099991-supplementary/Supplementary Material/Projections/Ensemble/fraudulenta/2100_RCP6.0_ensembled.pdf]

# Pseudo\_nitzchia\_fraudulenta\_2100\_RCP8.5

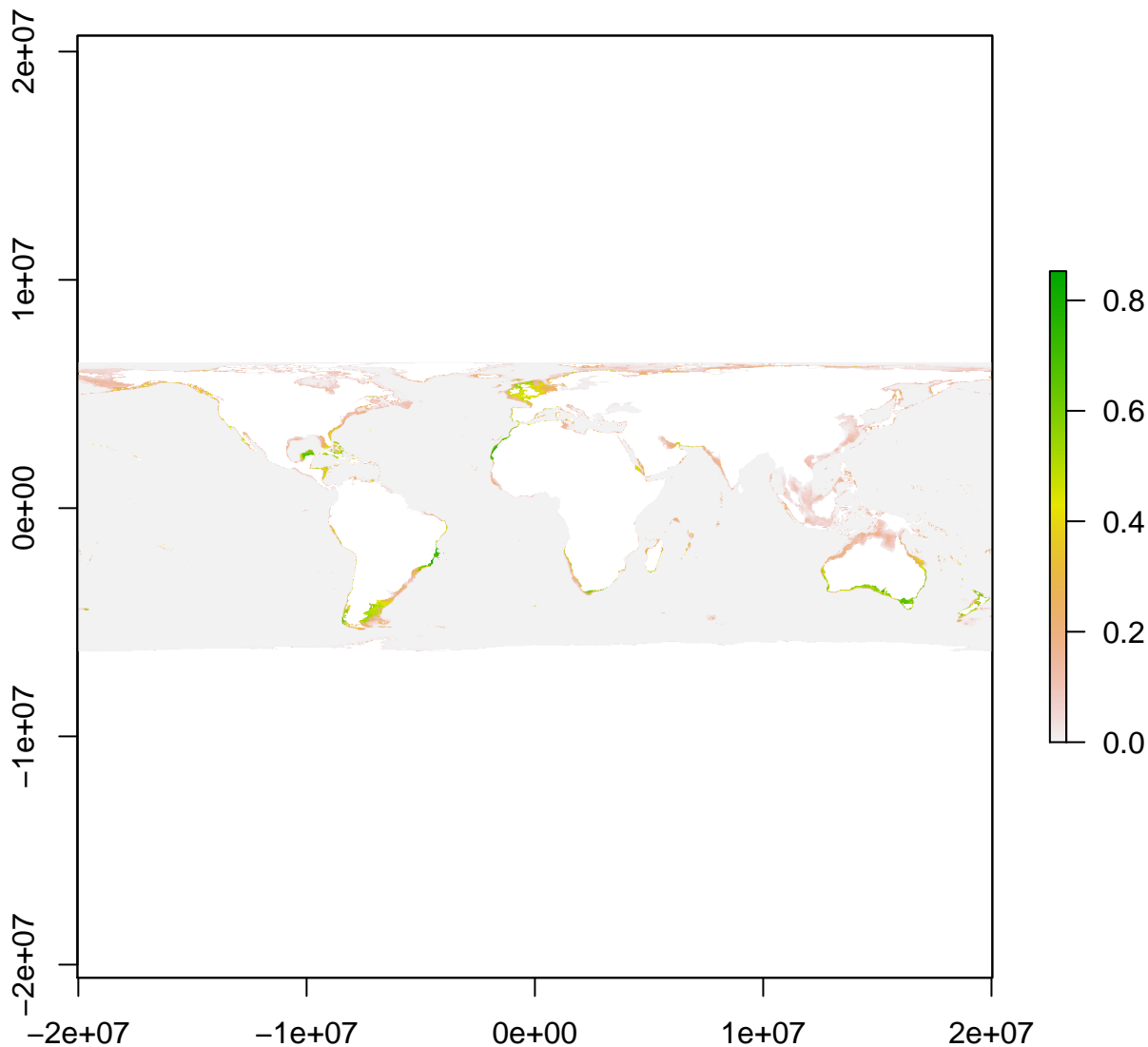

Supplement: Supplementary file 1 [file toxins-15-00009-s001.zip › toxins-2099991-supplementary/Supplementary Material/Projections/Ensemble/fraudulenta/2100_RCP8.5_ensembled.pdf]

# Pseudo\_nitzchia\_fraudulenta\_present

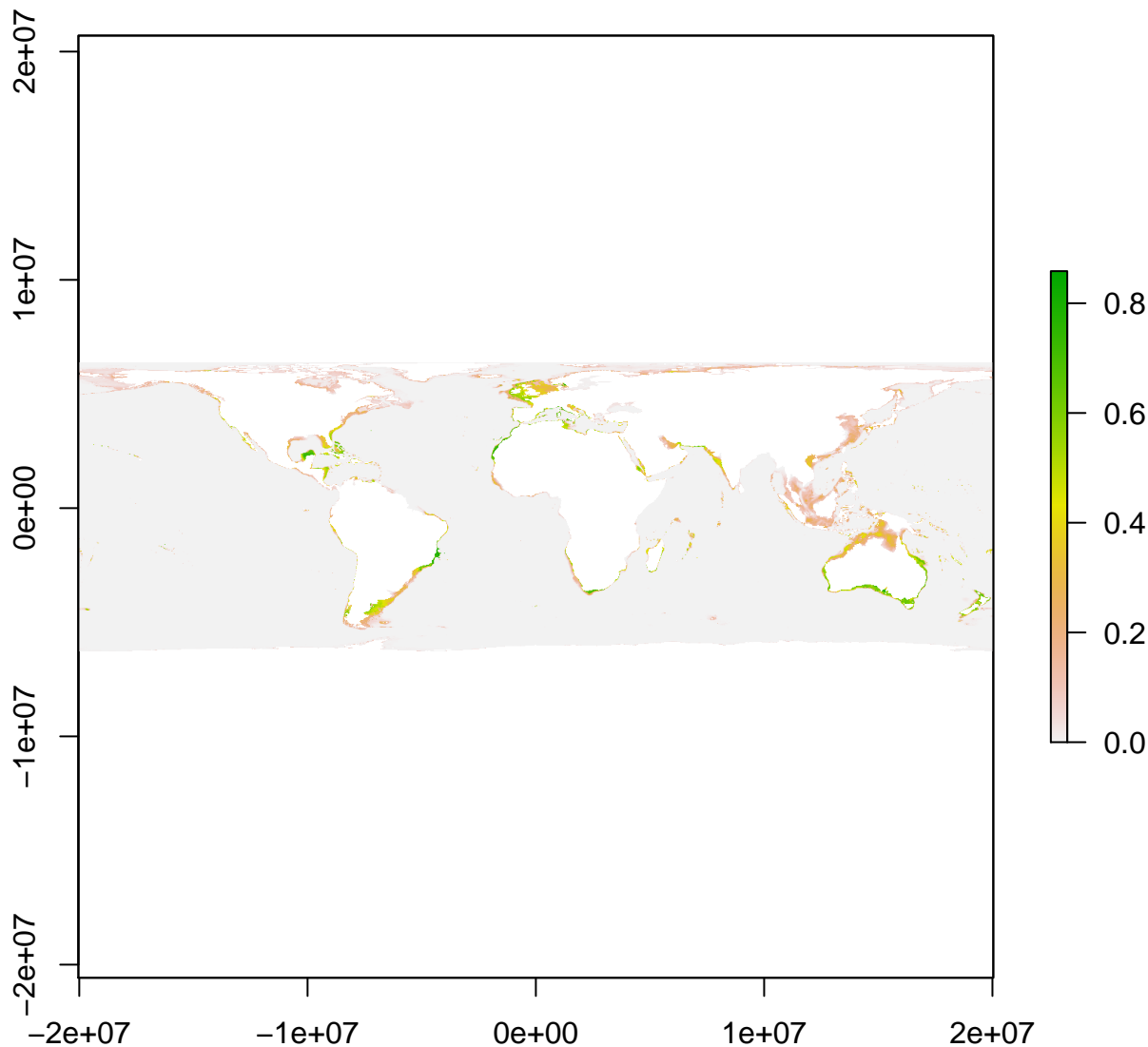

Supplement: Supplementary file 1 [file toxins-15-00009-s001.zip › toxins-2099991-supplementary/Supplementary Material/Projections/Ensemble/fraudulenta/present_ensembled.pdf]

# Pseudo\_nitzchia\_seriata\_2050\_RCP2.6

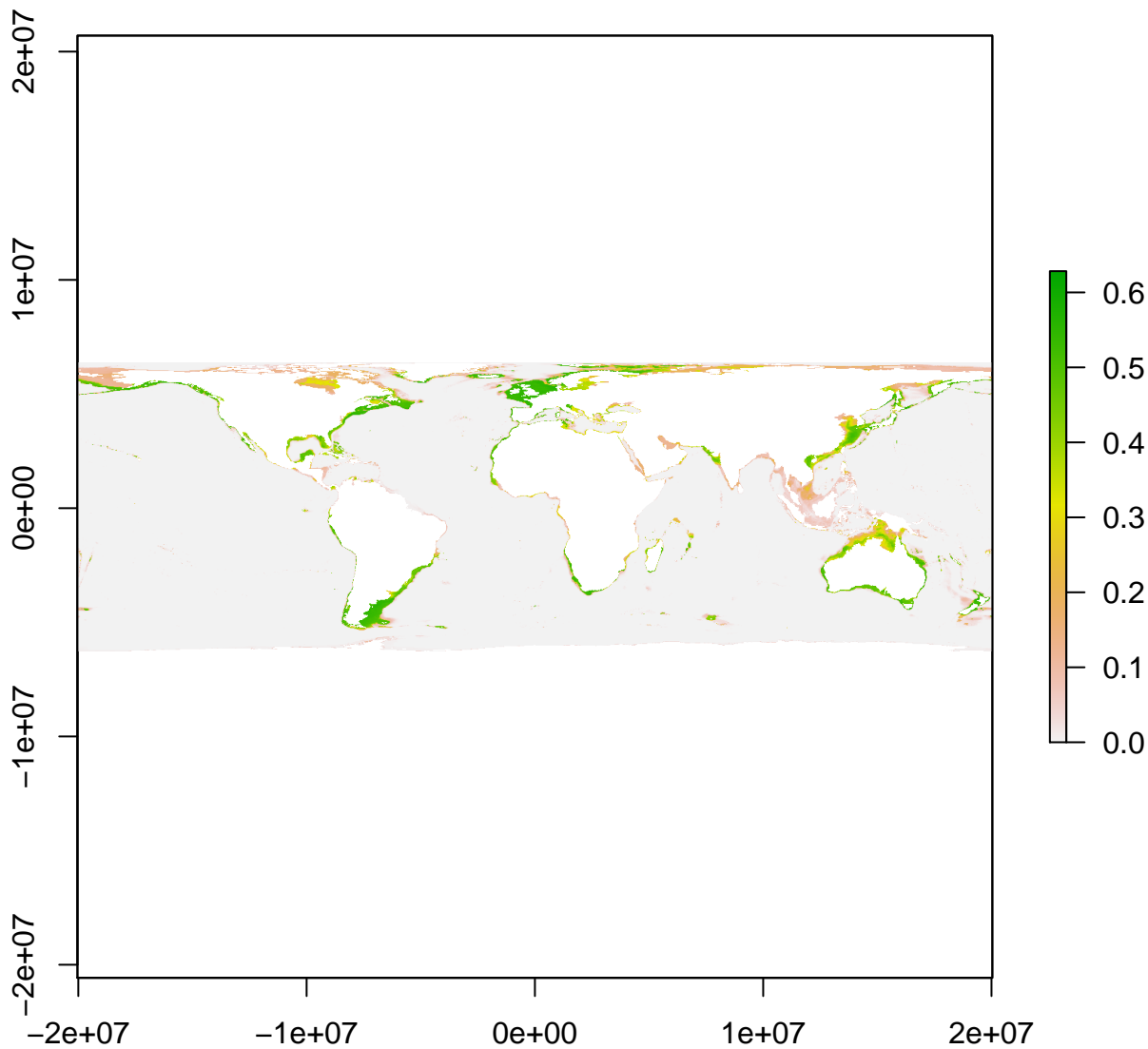

Supplement: Supplementary file 1 [file toxins-15-00009-s001.zip › toxins-2099991-supplementary/Supplementary Material/Projections/Ensemble/seriata/2050_RCP2.6_ensembled.pdf]

# Pseudo\_nitzchia\_serjata\_2050\_RCP4.5

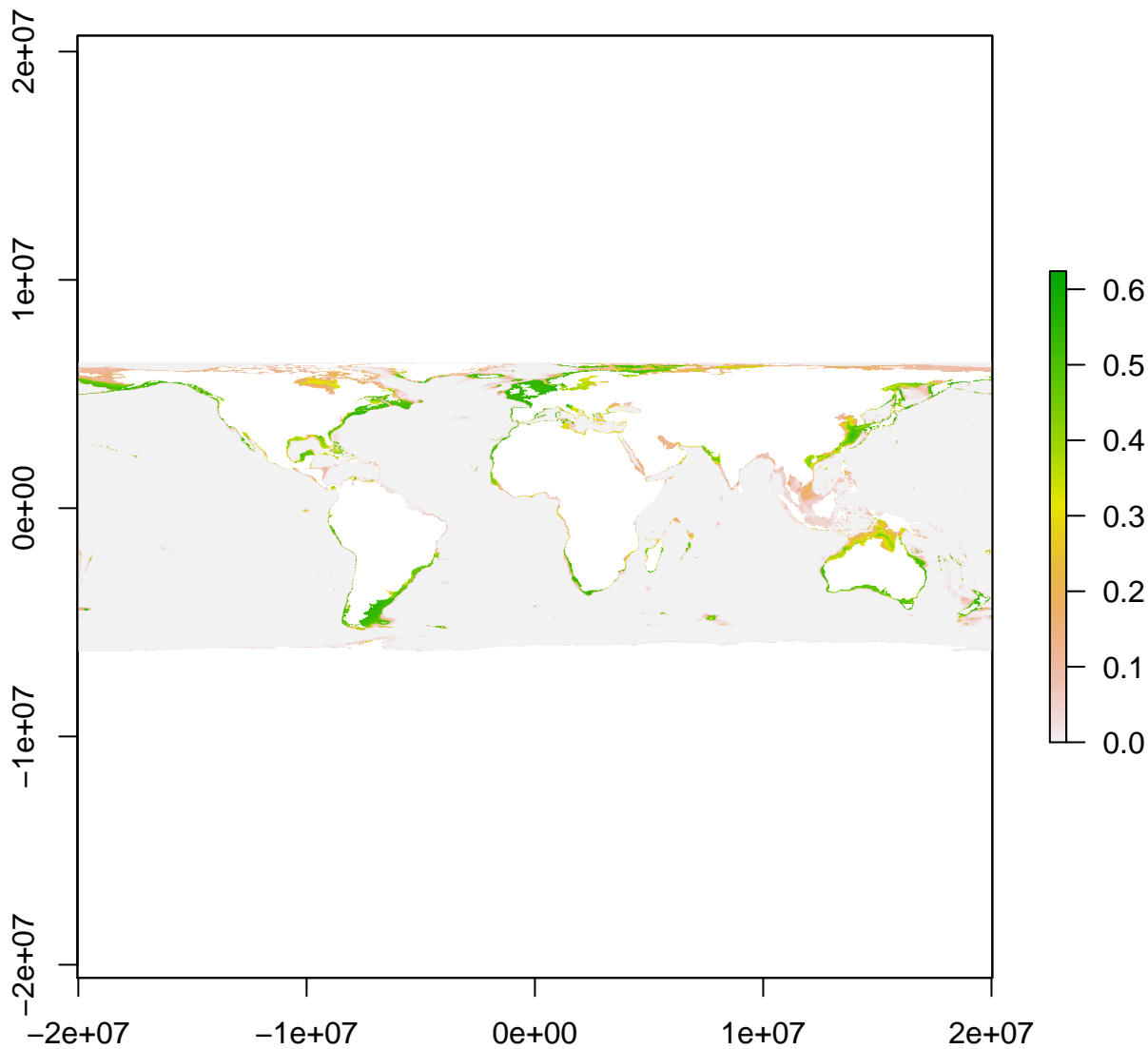

Supplement: Supplementary file 1 [file toxins-15-00009-s001.zip › toxins-2099991-supplementary/Supplementary Material/Projections/Ensemble/seriata/2050_RCP4.5_ensembled.pdf]

# Pseudo\_nitzchia\_seriata\_2050\_RCP6.0

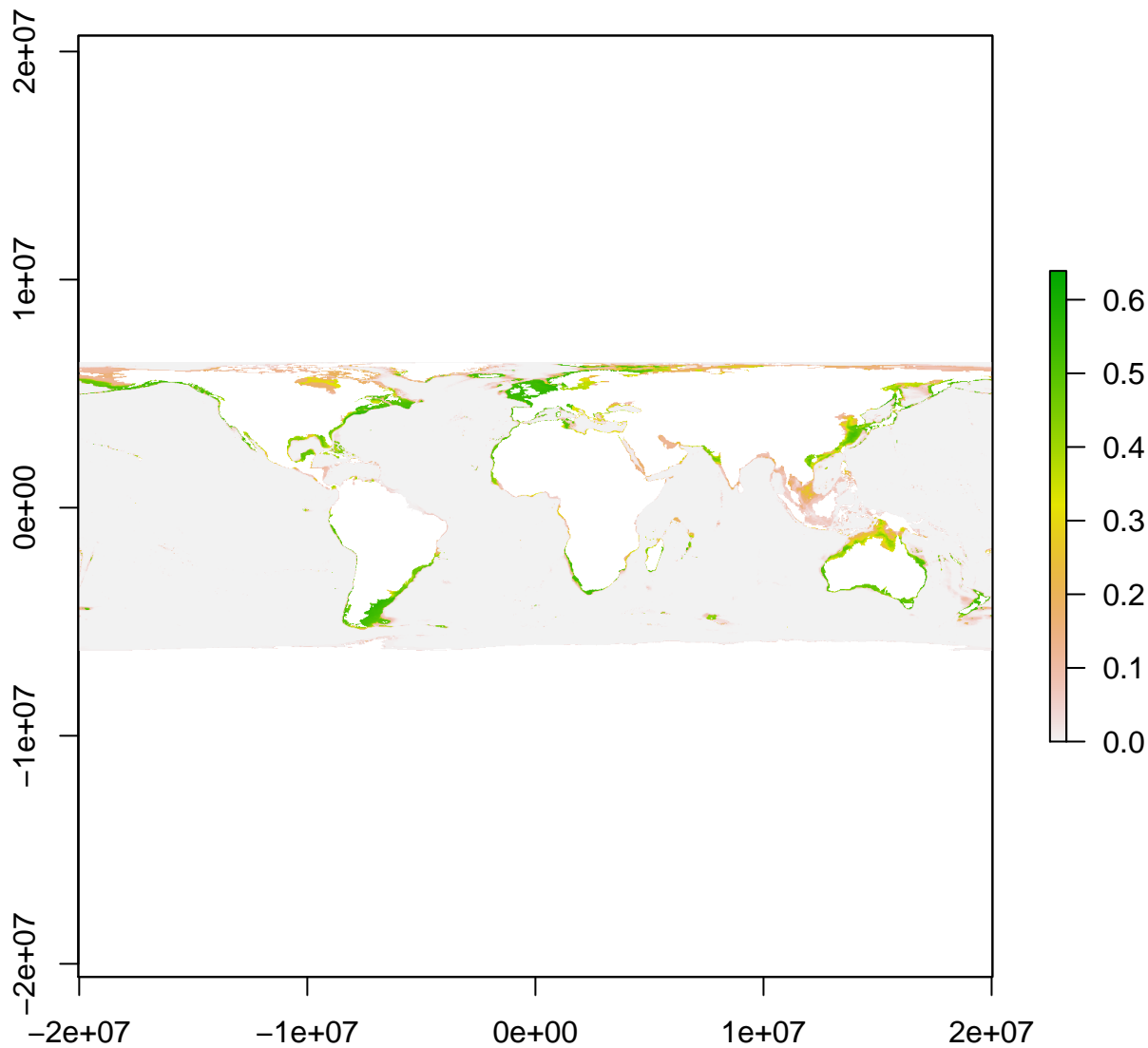

Supplement: Supplementary file 1 [file toxins-15-00009-s001.zip › toxins-2099991-supplementary/Supplementary Material/Projections/Ensemble/seriata/2050_RCP6.0_ensembled.pdf]

# Pseudo\_nitzchia\_serziata\_2050\_RCP8.5

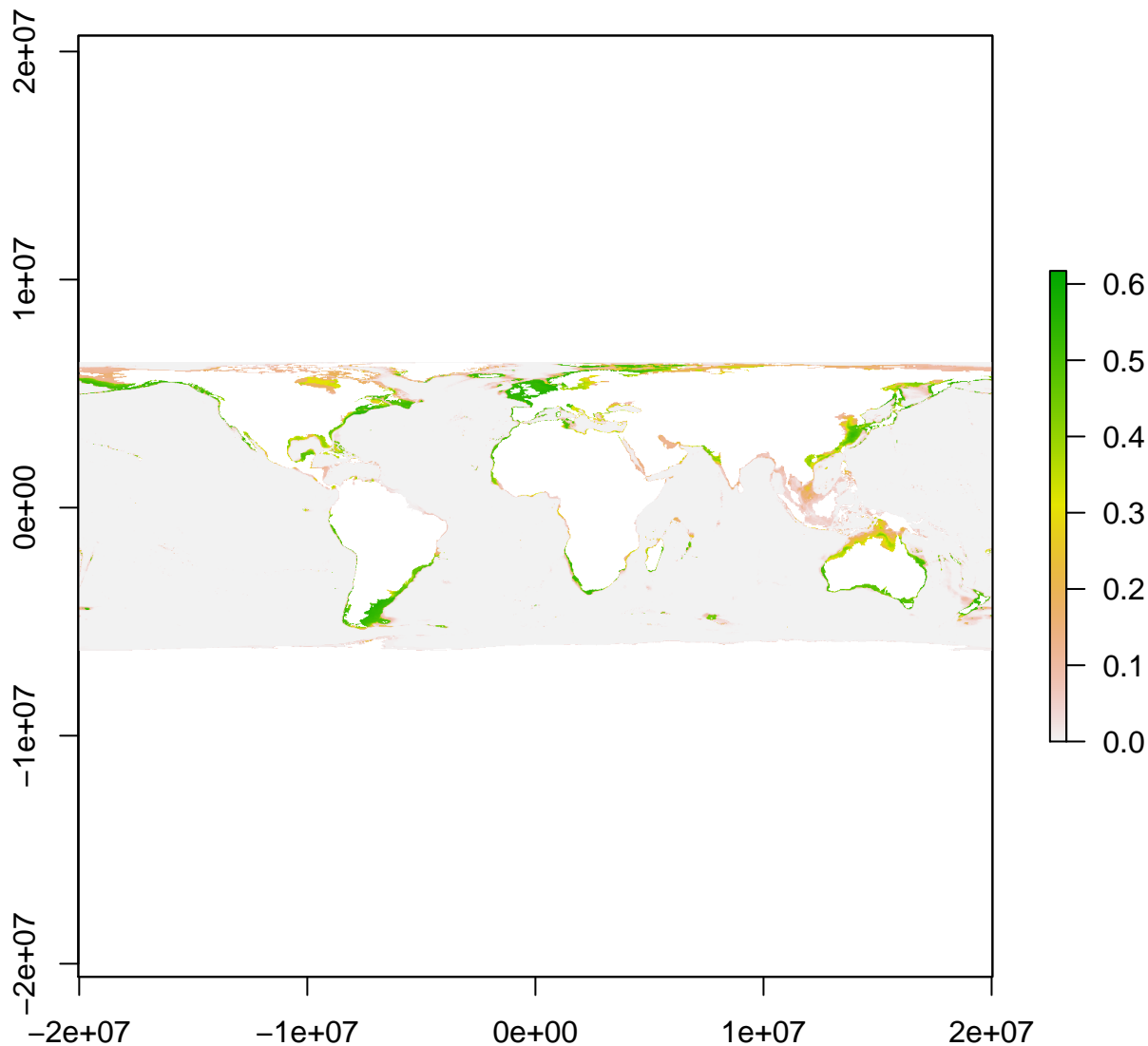

Supplement: Supplementary file 1 [file toxins-15-00009-s001.zip › toxins-2099991-supplementary/Supplementary Material/Projections/Ensemble/seriata/2050_RCP8.5_ensembled.pdf]

# Pseudo\_nitzchia\_serjata\_2100\_RCP2.6

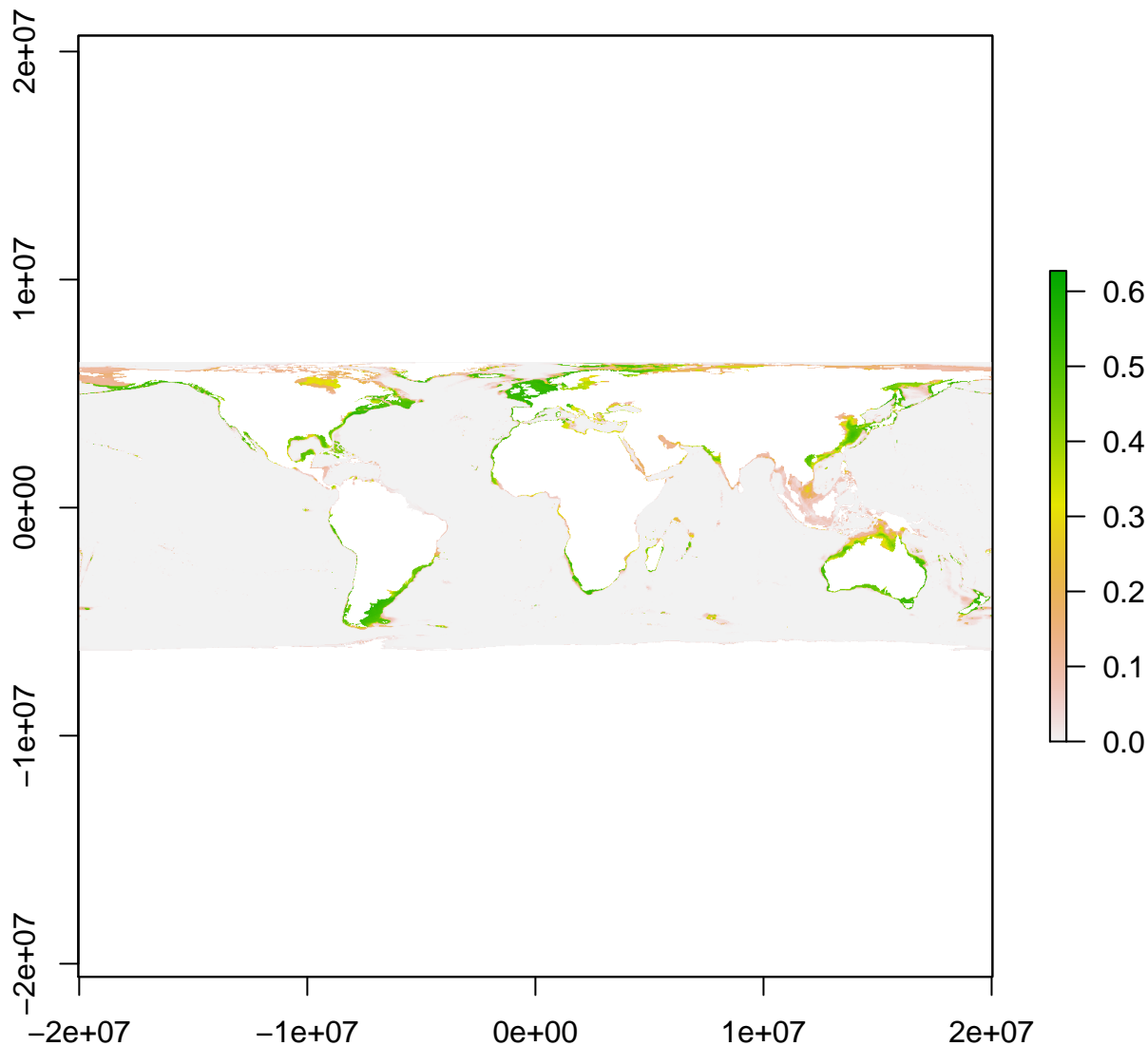

Supplement: Supplementary file 1 [file toxins-15-00009-s001.zip › toxins-2099991-supplementary/Supplementary Material/Projections/Ensemble/seriata/2100_RCP2.6_ensembled.pdf]

# Pseudo\_nitzchia\_seriata\_2100\_RCP4.5

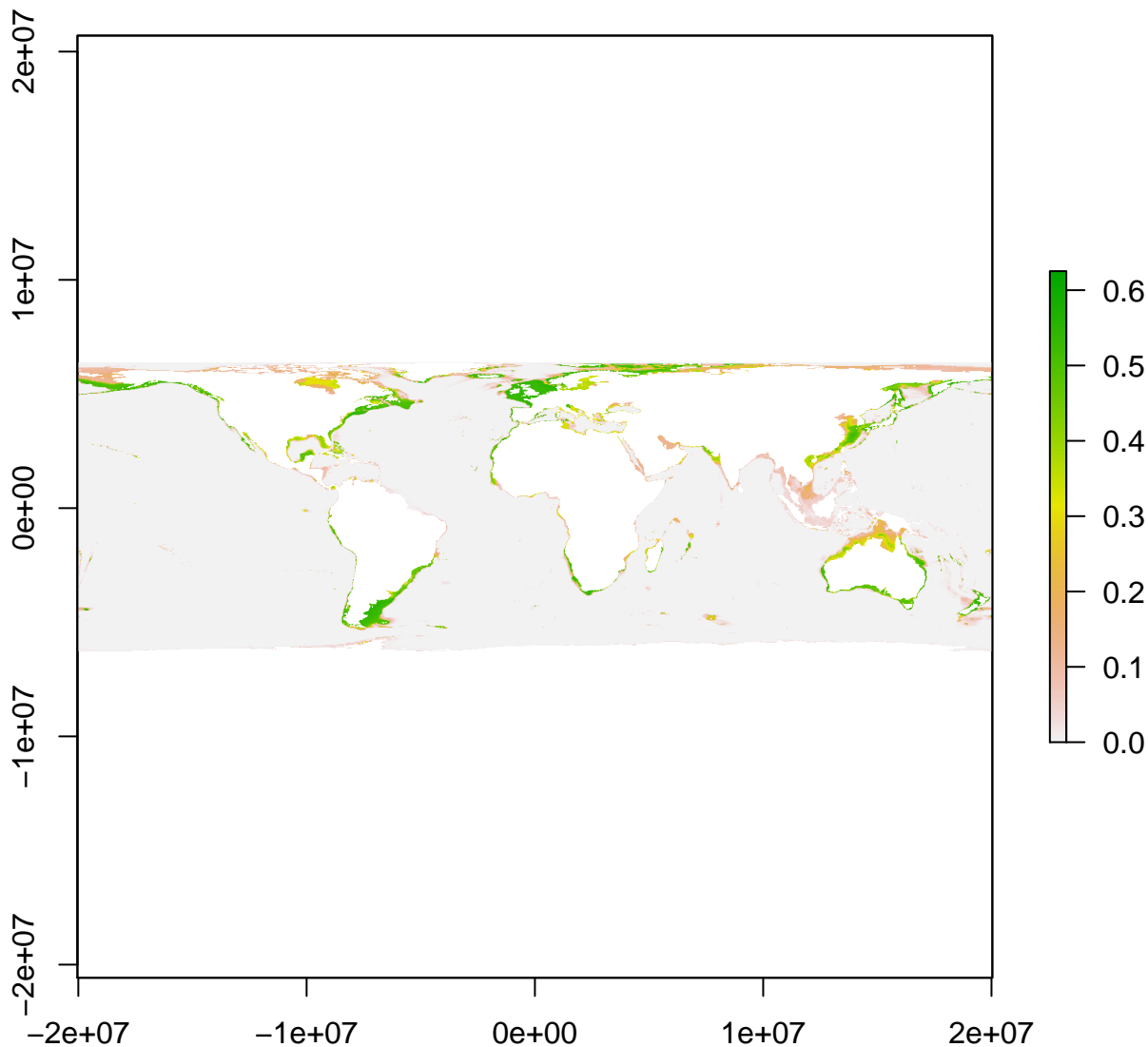

Supplement: Supplementary file 1 [file toxins-15-00009-s001.zip › toxins-2099991-supplementary/Supplementary Material/Projections/Ensemble/seriata/2100_RCP4.5_ensembled.pdf]

# Pseudo\_nitzchia\_seriata\_2100\_RCP6.0

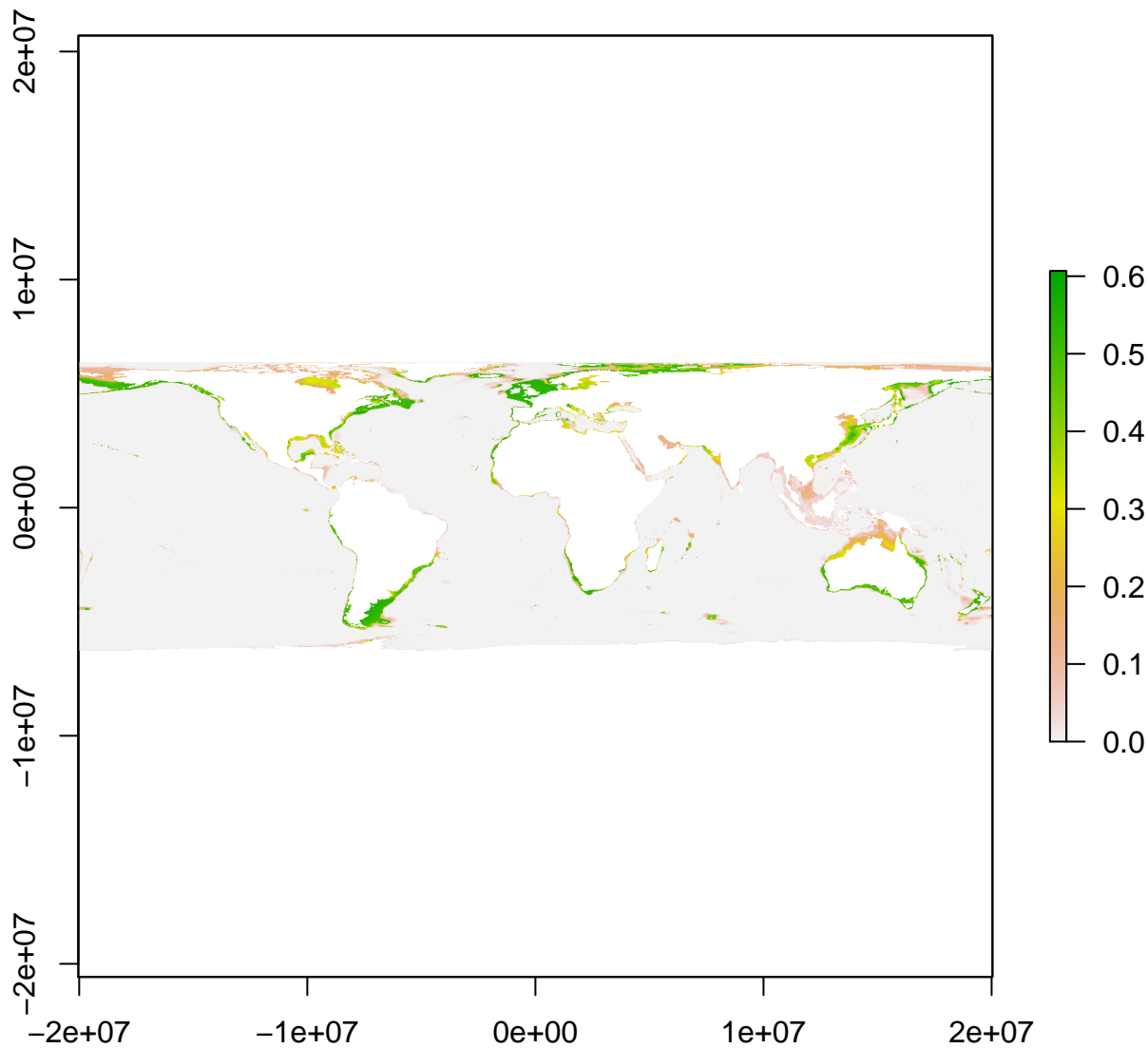

Supplement: Supplementary file 1 [file toxins-15-00009-s001.zip › toxins-2099991-supplementary/Supplementary Material/Projections/Ensemble/seriata/2100_RCP6.0_ensembled.pdf]

# Pseudo\_nitzchia\_serjata\_2100\_RCP8.5

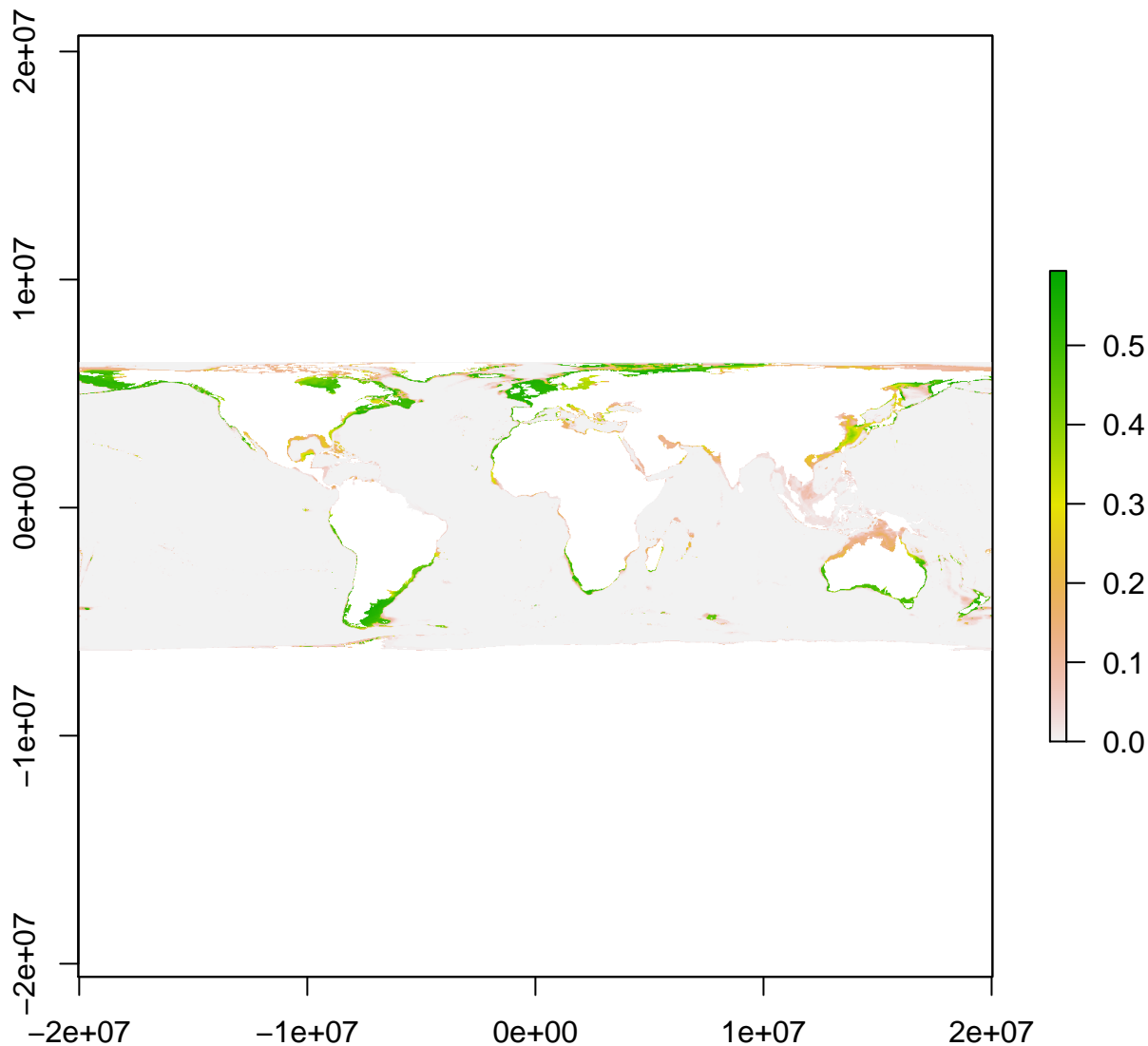

Supplement: Supplementary file 1 [file toxins-15-00009-s001.zip › toxins-2099991-supplementary/Supplementary Material/Projections/Ensemble/seriata/2100_RCP8.5_ensembled.pdf]

# Pseudo\_nitzchia\_seriata\_present

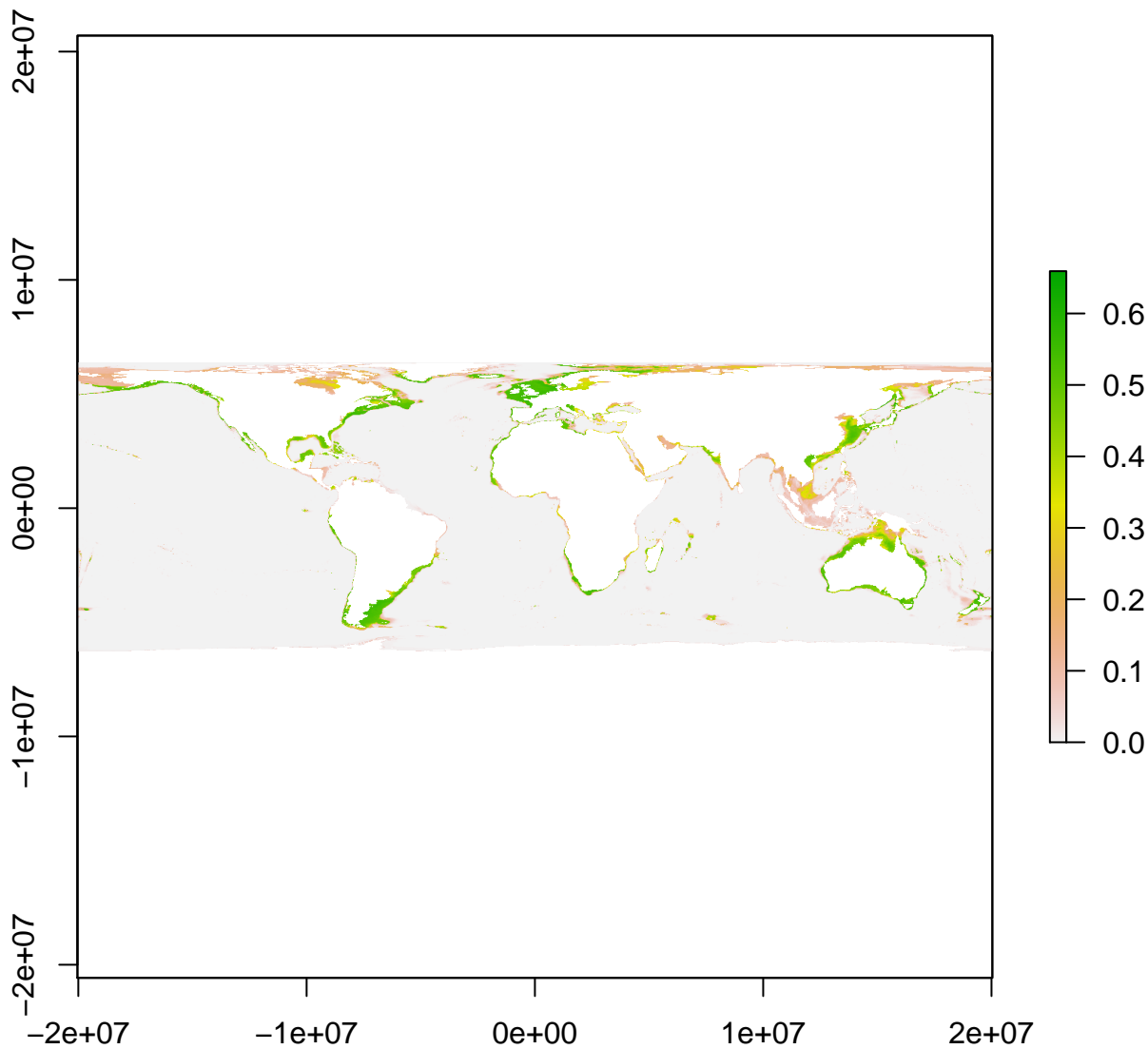

Supplement: Supplementary file 1 [file toxins-15-00009-s001.zip › toxins-2099991-supplementary/Supplementary Material/Projections/Ensemble/seriata/present_ensembled.pdf]

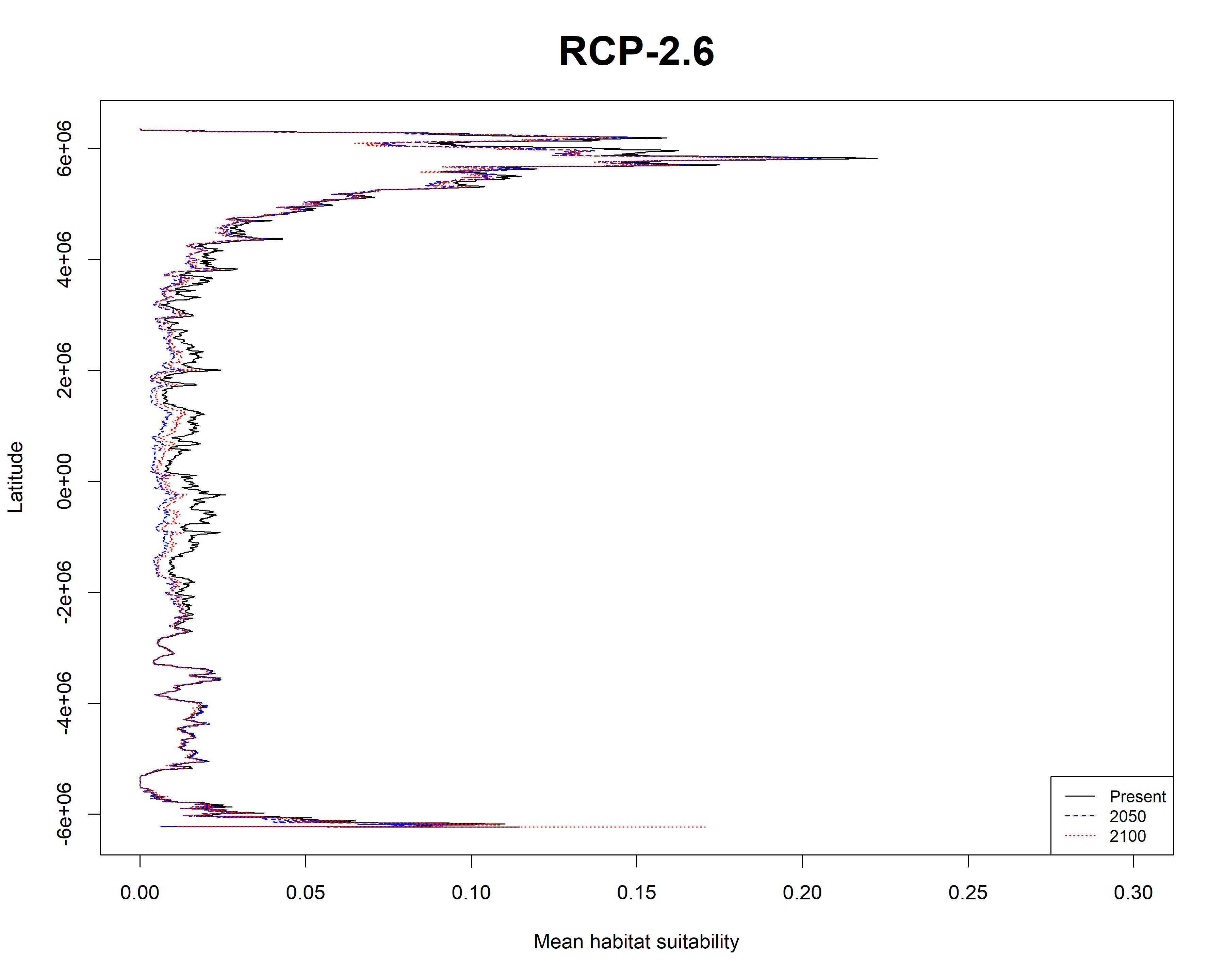

Supplement: Supplementary file 1 [file toxins-15-00009-s001.zip › toxins-2099991-supplementary/Supplementary Material/Projections/Latitudinal_habitat_suitability/lat_hab_australis_26.tiff]

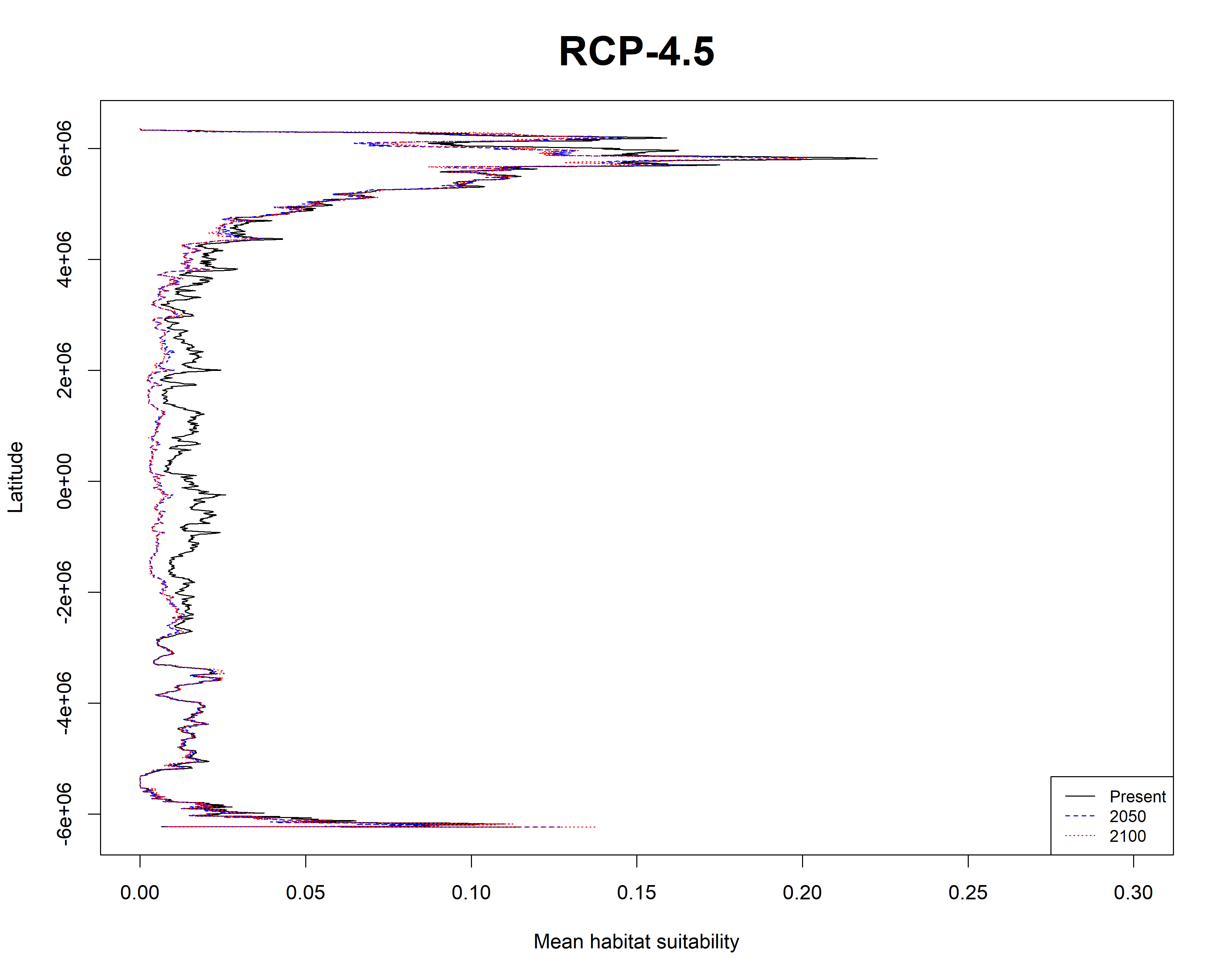

Supplement: Supplementary file 1 [file toxins-15-00009-s001.zip › toxins-2099991-supplementary/Supplementary Material/Projections/Latitudinal_habitat_suitability/lat_hab_australis_45.tiff]

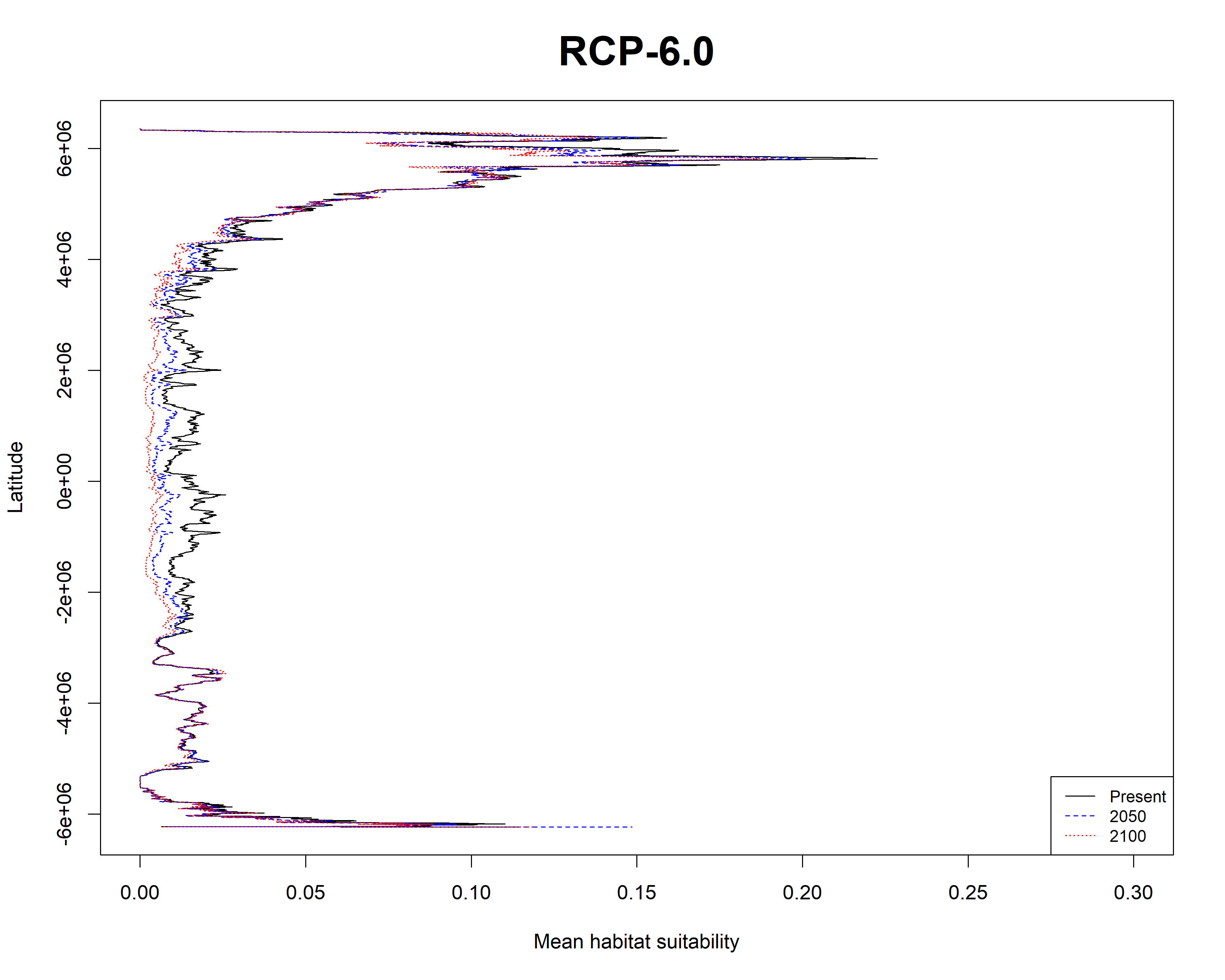

Supplement: Supplementary file 1 [file toxins-15-00009-s001.zip › toxins-2099991-supplementary/Supplementary Material/Projections/Latitudinal_habitat_suitability/lat_hab_australis_60.tiff]

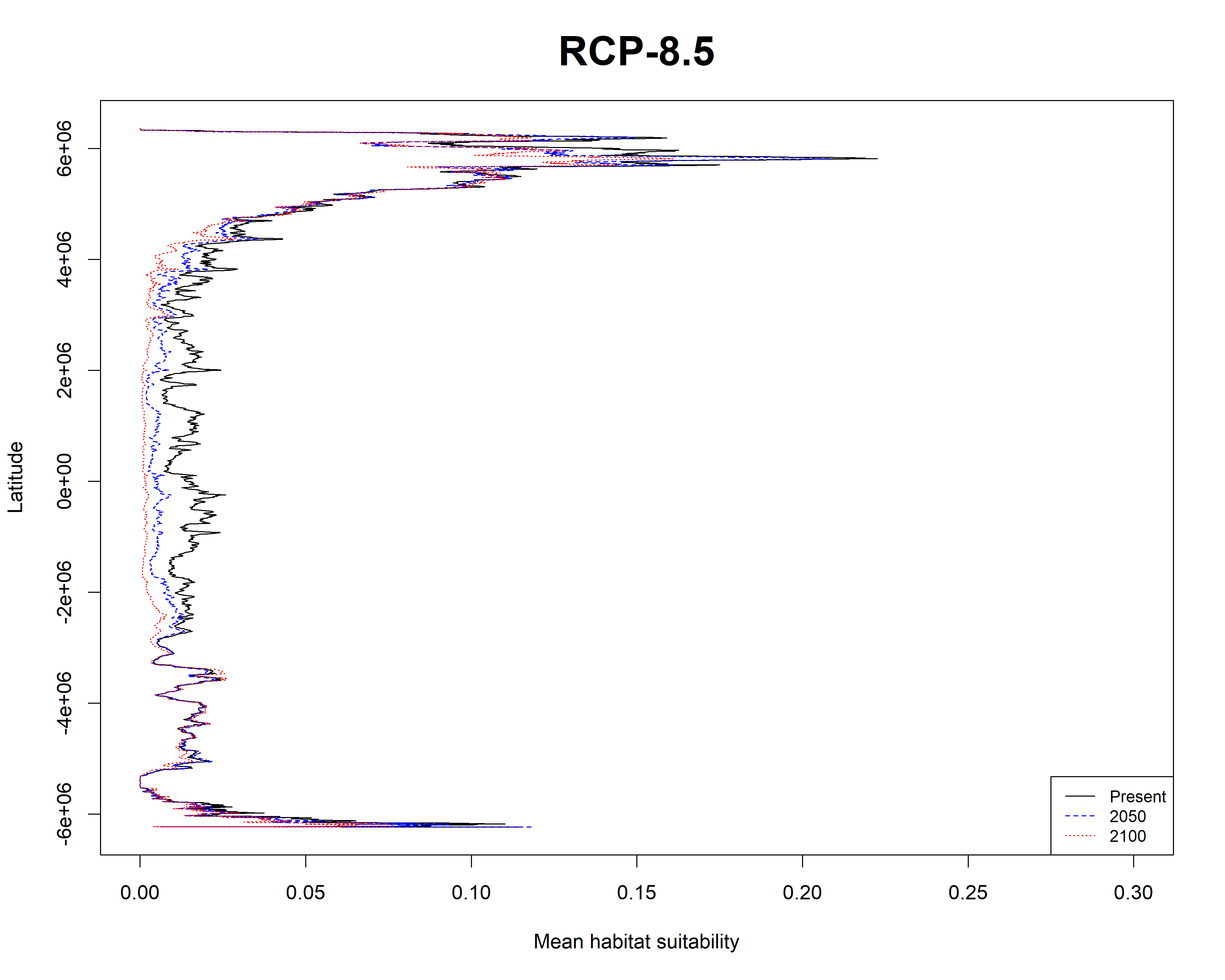

Supplement: Supplementary file 1 [file toxins-15-00009-s001.zip › toxins-2099991-supplementary/Supplementary Material/Projections/Latitudinal_habitat_suitability/lat_hab_australis_85.tiff]

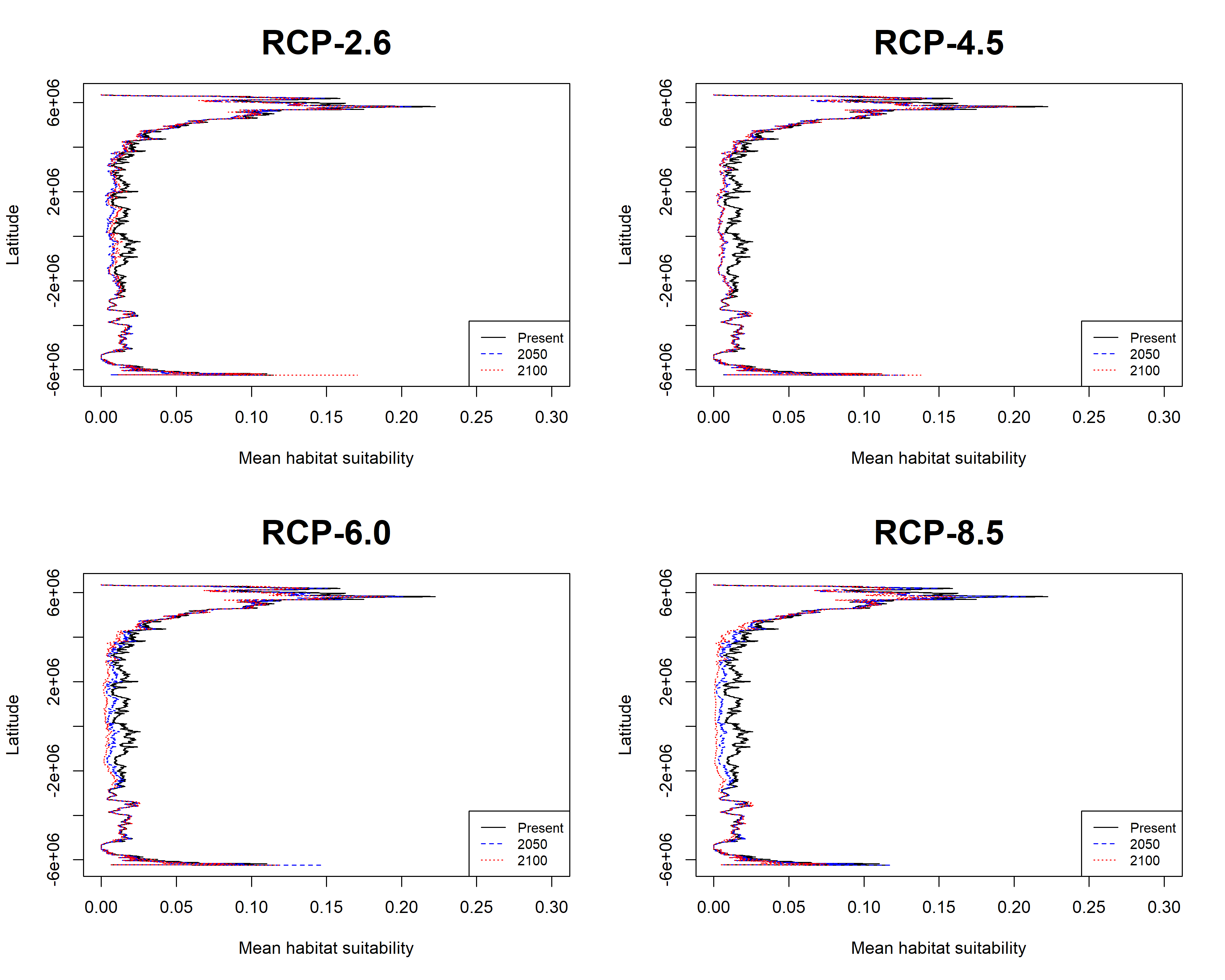

Supplement: Supplementary file 1 [file toxins-15-00009-s001.zip › toxins-2099991-supplementary/Supplementary Material/Projections/Latitudinal_habitat_suitability/lat_hab_australis_full.tiff]

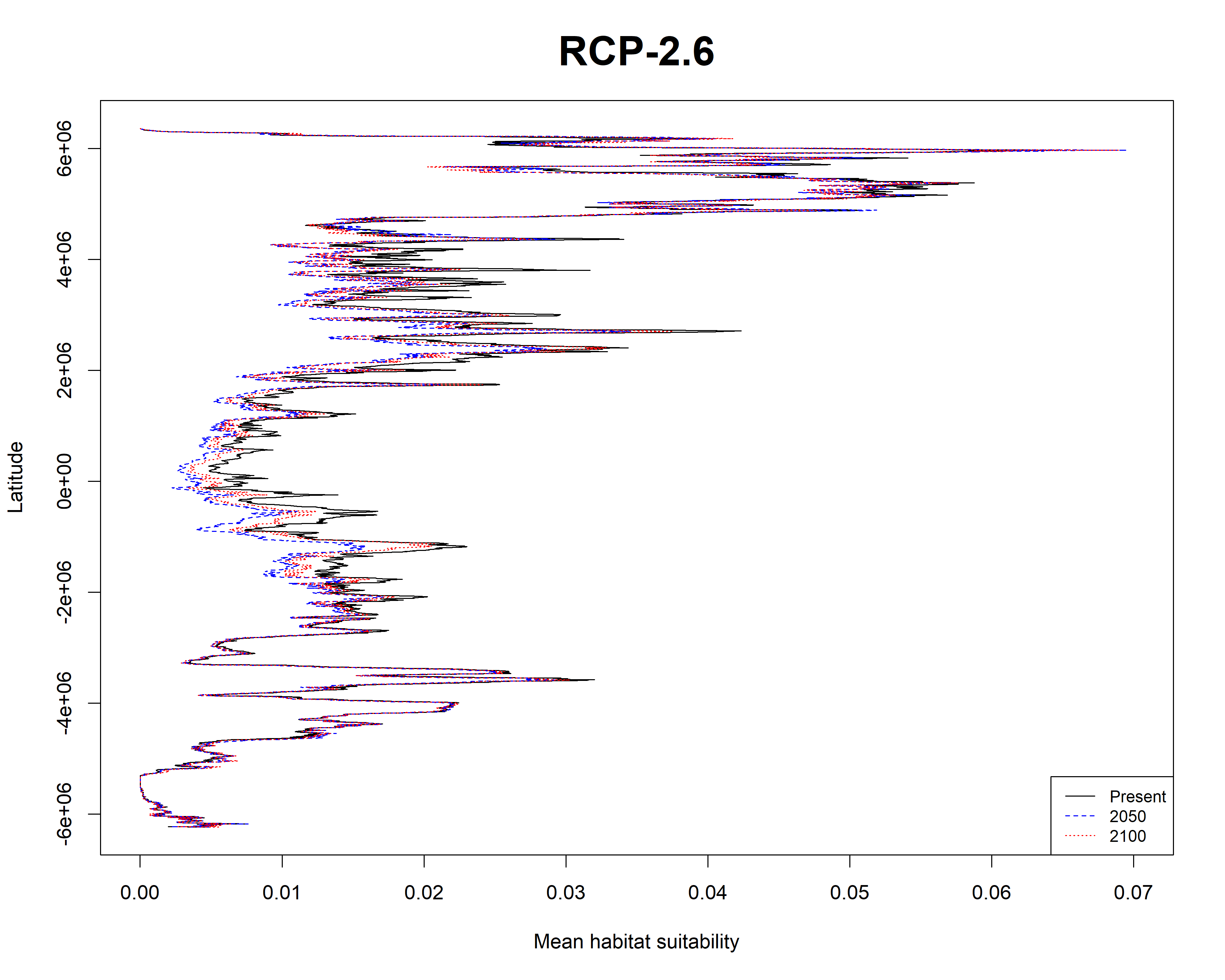

Supplement: Supplementary file 1 [file toxins-15-00009-s001.zip › toxins-2099991-supplementary/Supplementary Material/Projections/Latitudinal_habitat_suitability/lat_hab_fraudulenta26.tiff]

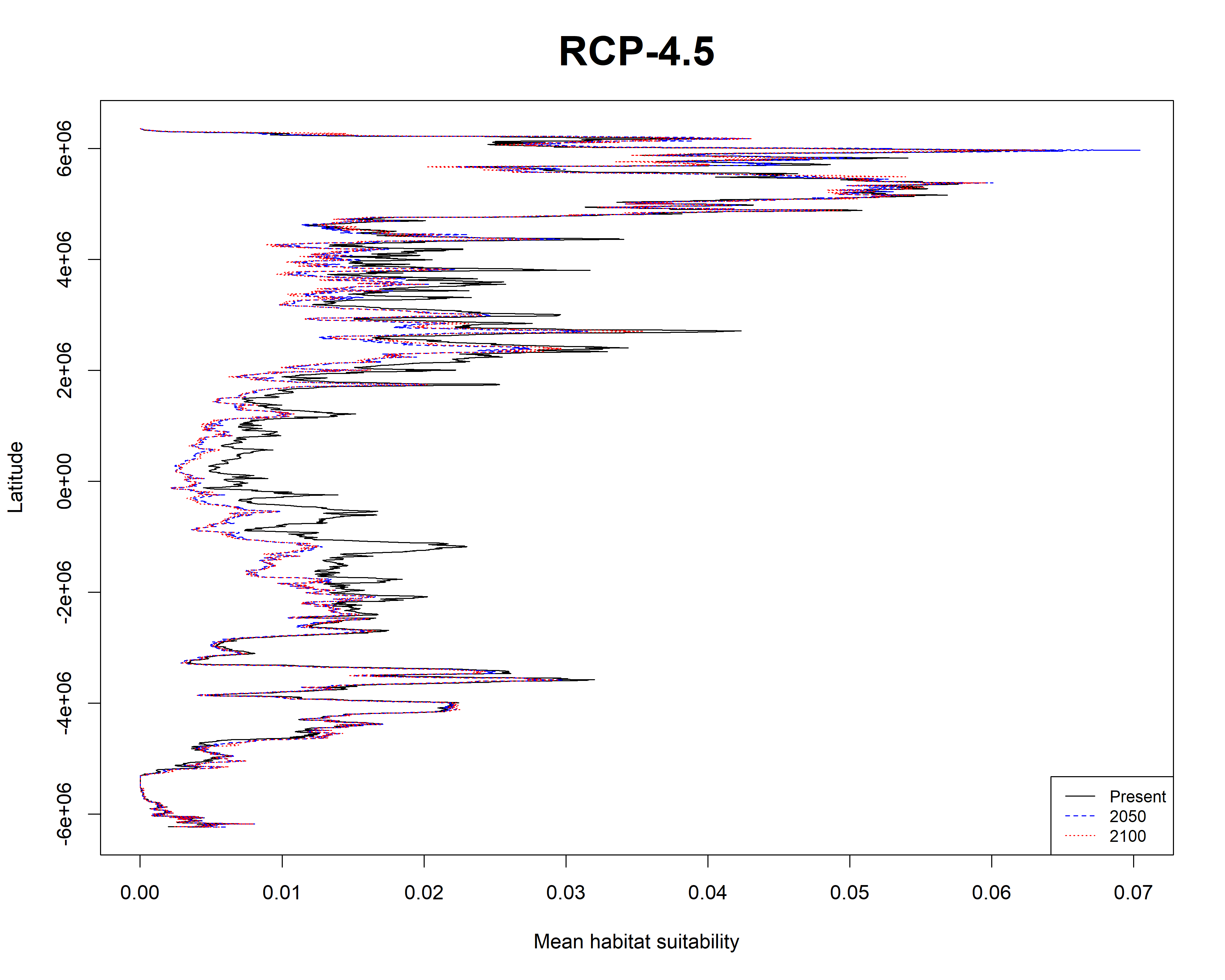

Supplement: Supplementary file 1 [file toxins-15-00009-s001.zip › toxins-2099991-supplementary/Supplementary Material/Projections/Latitudinal_habitat_suitability/lat_hab_fraudulenta45.tiff]

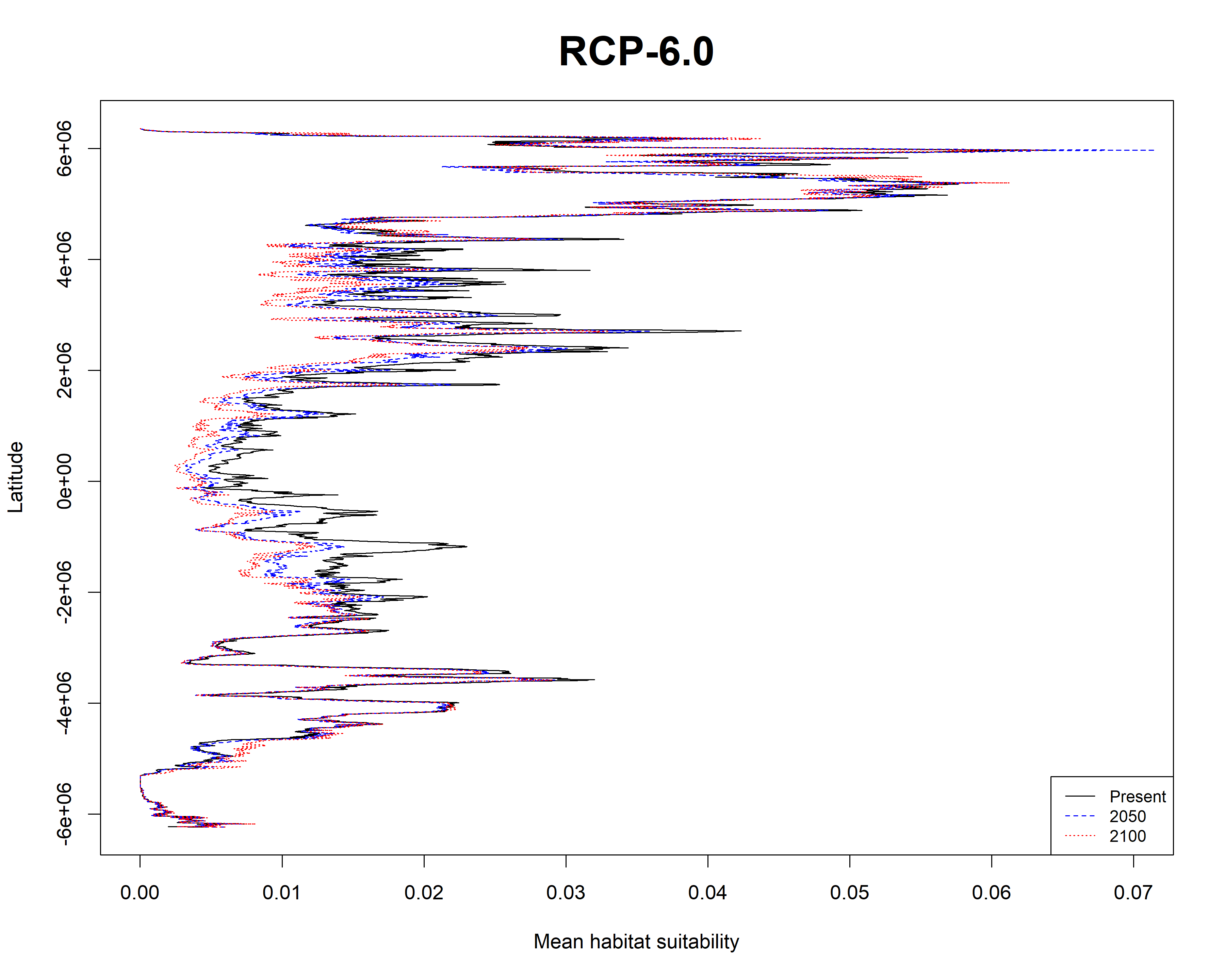

Supplement: Supplementary file 1 [file toxins-15-00009-s001.zip › toxins-2099991-supplementary/Supplementary Material/Projections/Latitudinal_habitat_suitability/lat_hab_fraudulenta60.tiff]

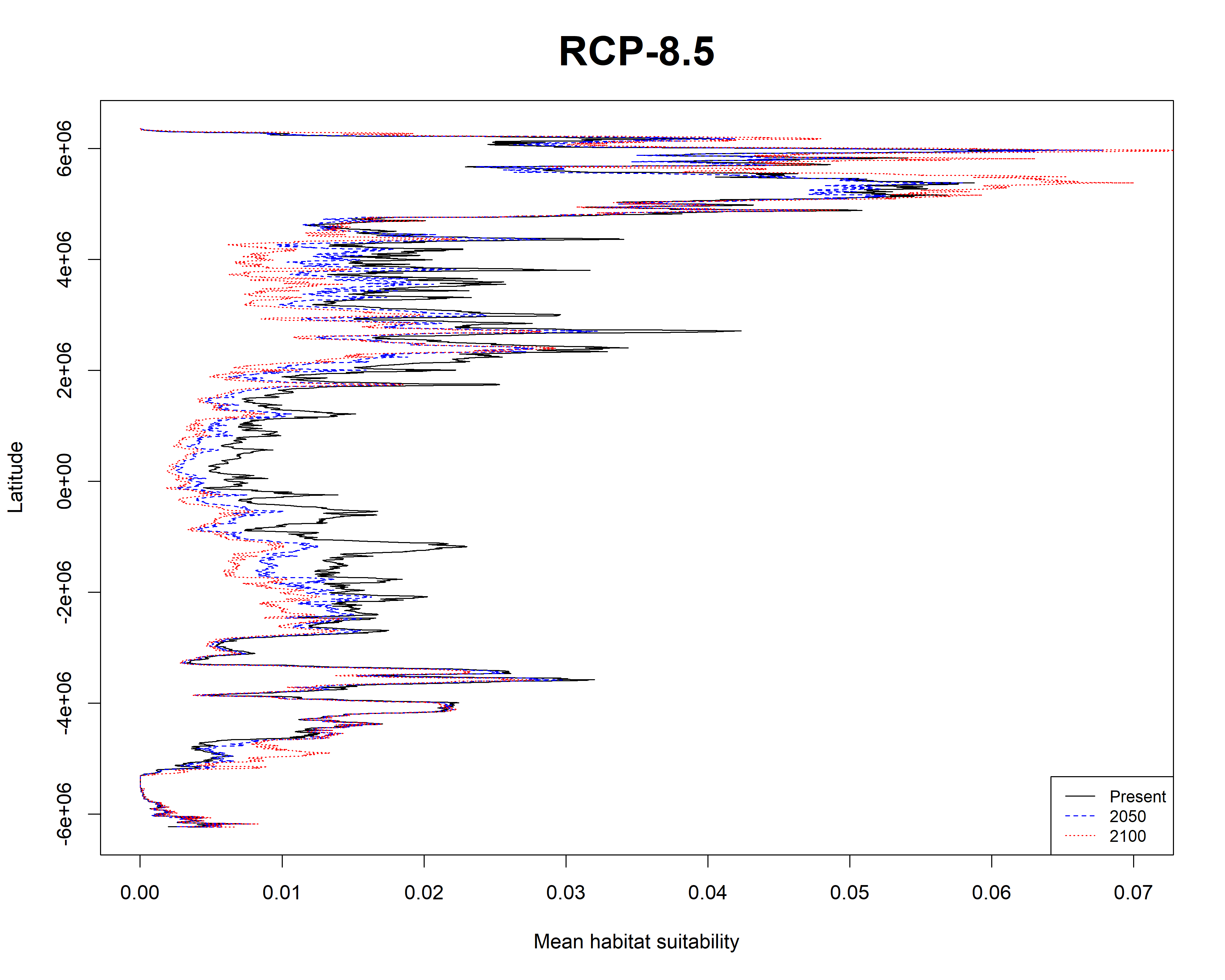

Supplement: Supplementary file 1 [file toxins-15-00009-s001.zip › toxins-2099991-supplementary/Supplementary Material/Projections/Latitudinal_habitat_suitability/lat_hab_fraudulenta85.tiff]

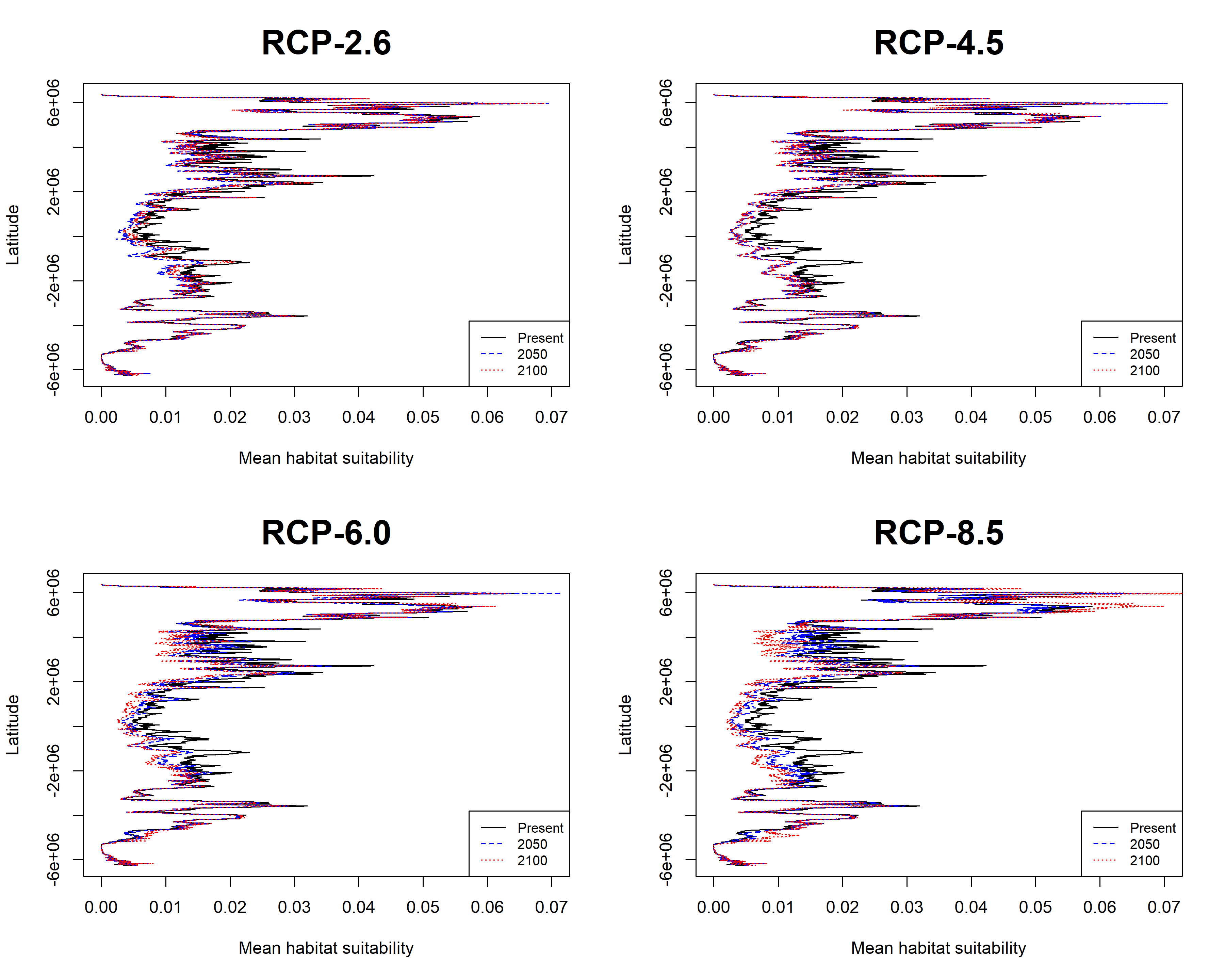

Supplement: Supplementary file 1 [file toxins-15-00009-s001.zip › toxins-2099991-supplementary/Supplementary Material/Projections/Latitudinal_habitat_suitability/lat_hab_fraudulenta_full.tiff]

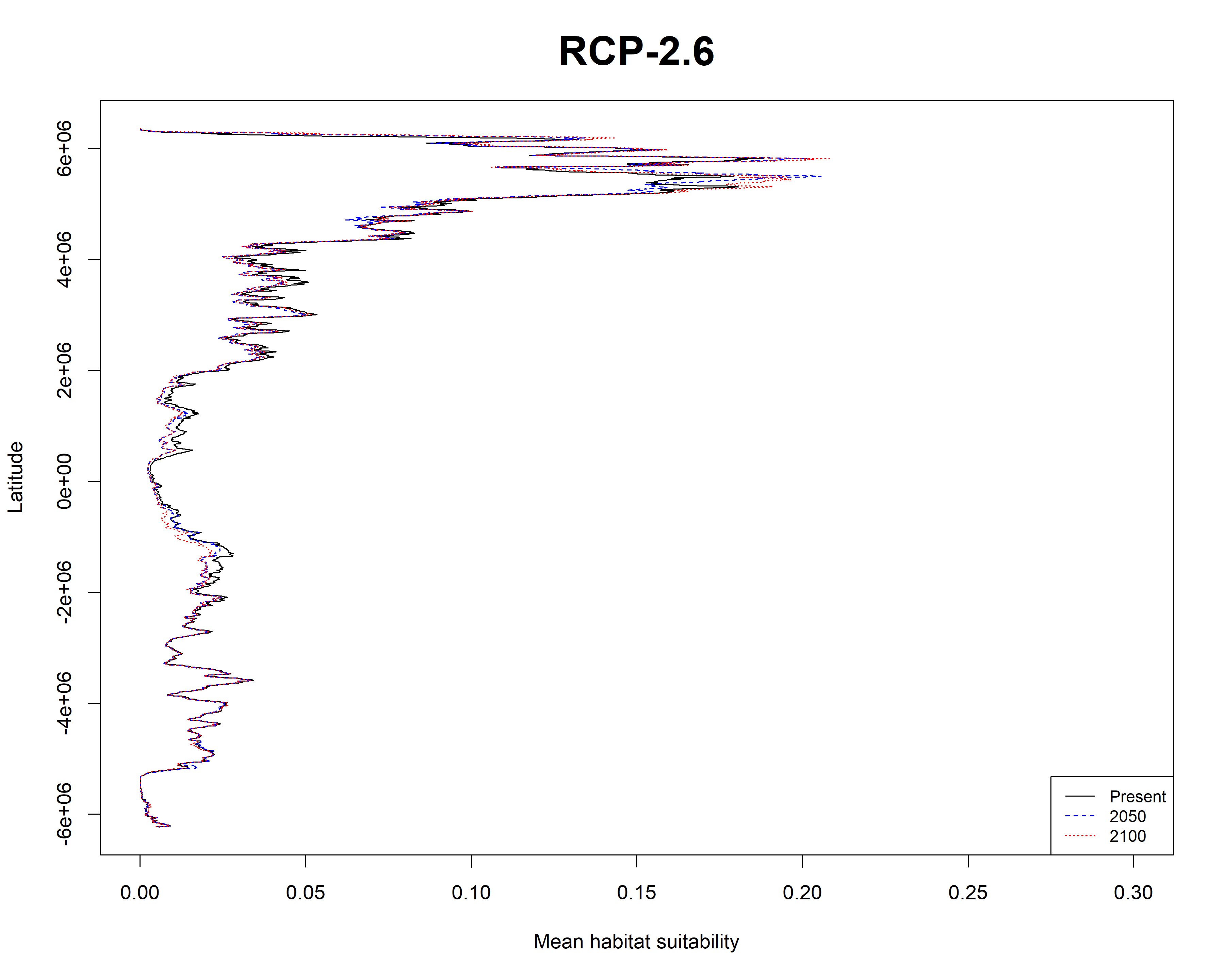

Supplement: Supplementary file 1 [file toxins-15-00009-s001.zip › toxins-2099991-supplementary/Supplementary Material/Projections/Latitudinal_habitat_suitability/lat_hab_seriata_26.tiff]

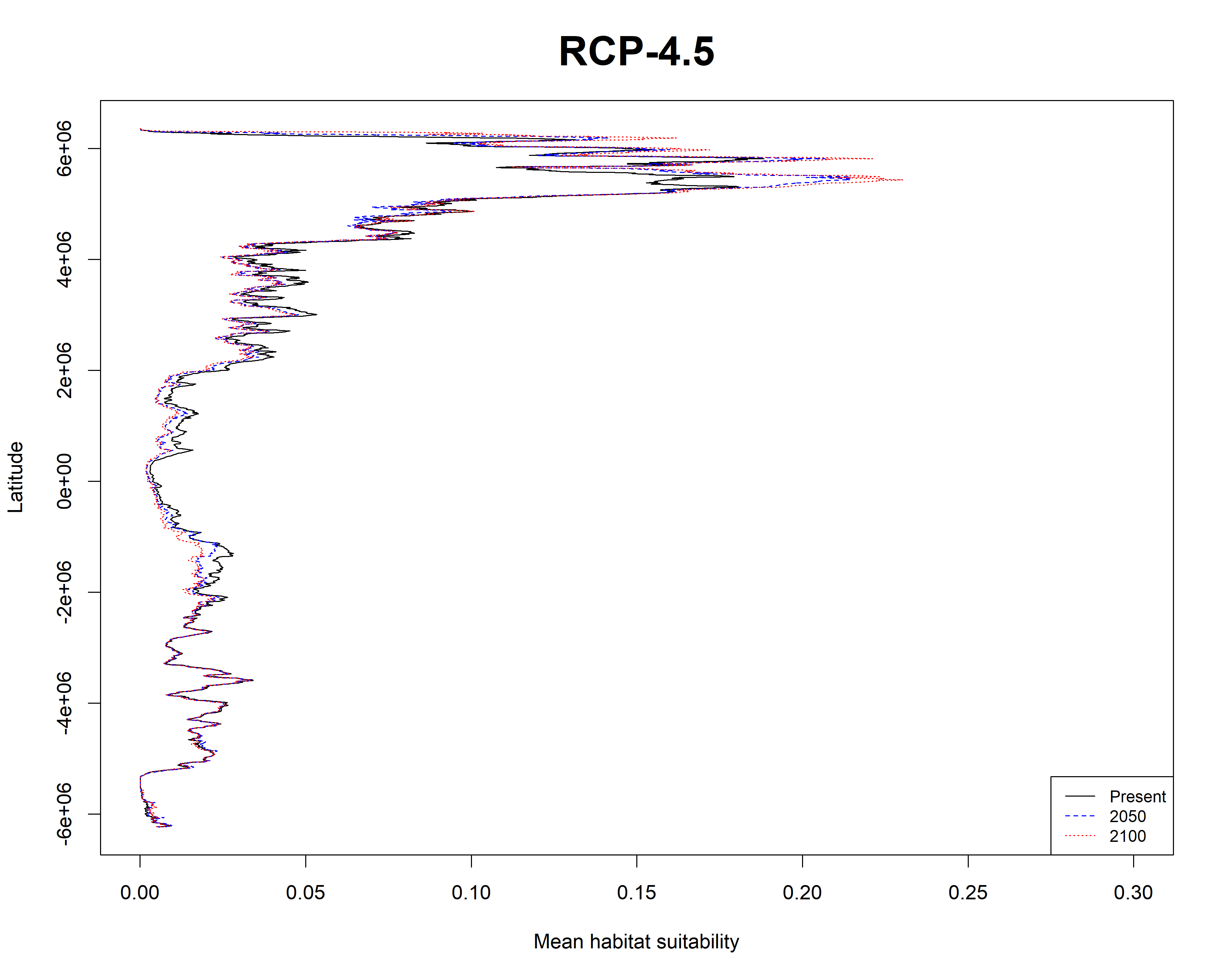

Supplement: Supplementary file 1 [file toxins-15-00009-s001.zip › toxins-2099991-supplementary/Supplementary Material/Projections/Latitudinal_habitat_suitability/lat_hab_seriata_45.tiff]

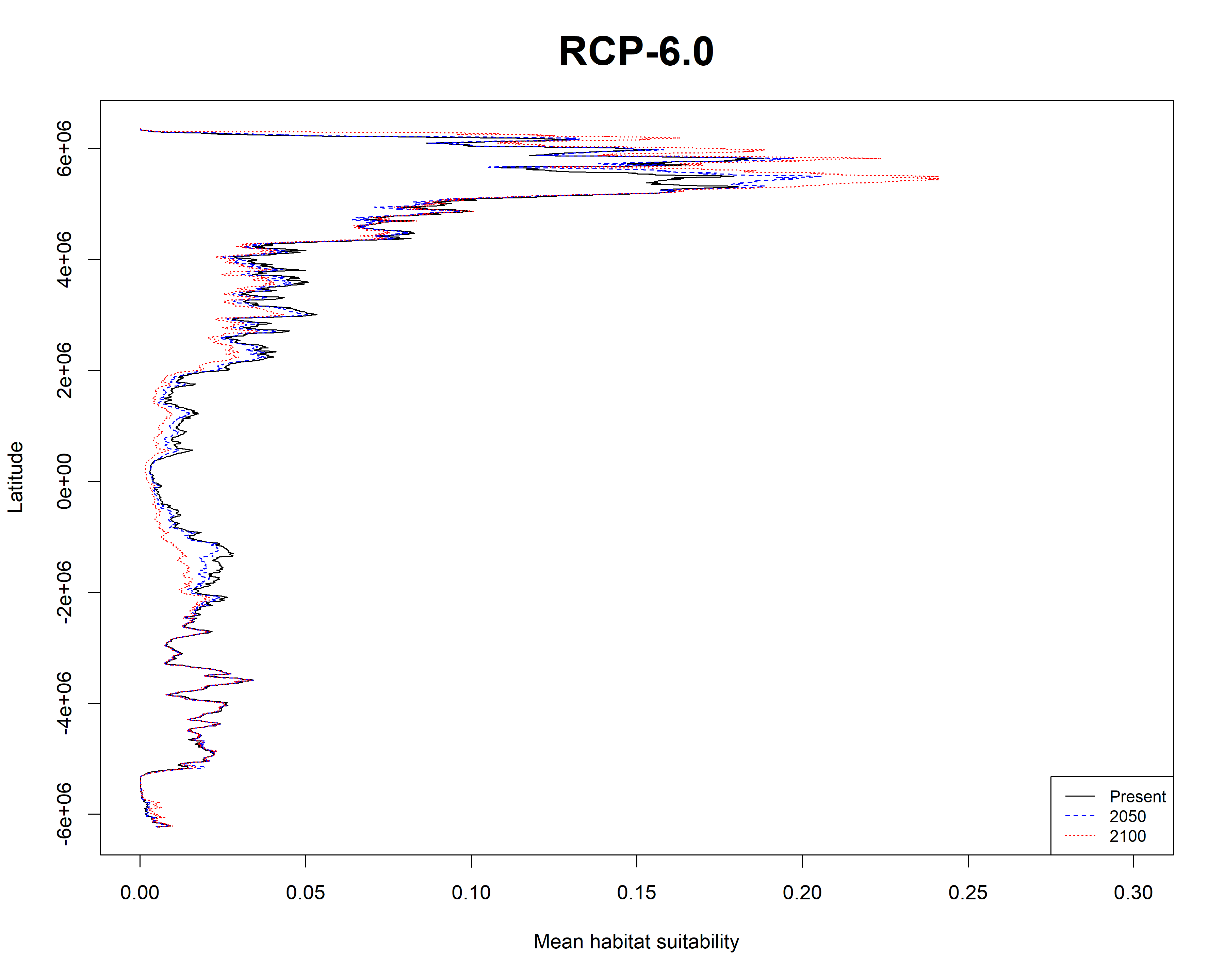

Supplement: Supplementary file 1 [file toxins-15-00009-s001.zip › toxins-2099991-supplementary/Supplementary Material/Projections/Latitudinal_habitat_suitability/lat_hab_seriata_60.tiff]

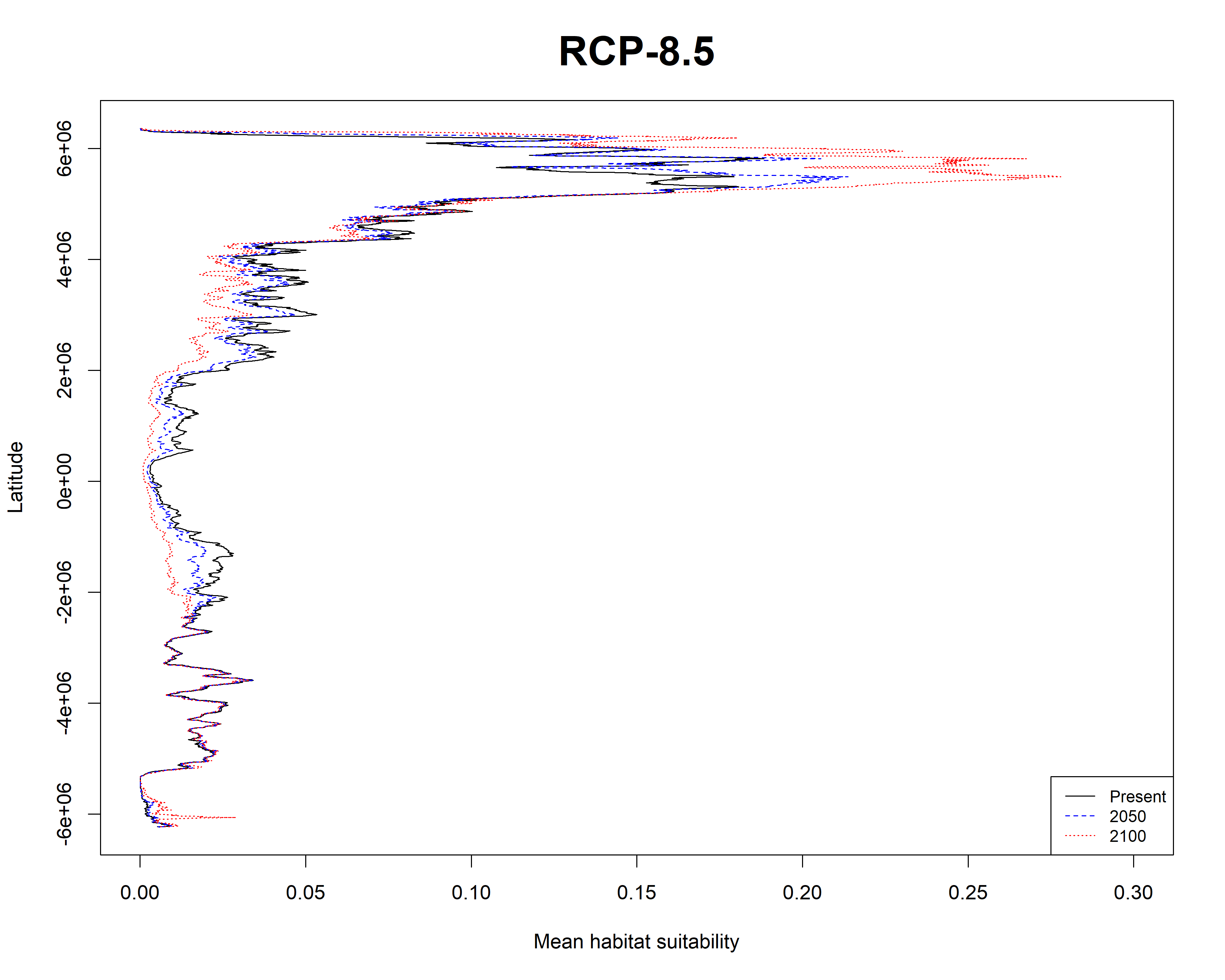

Supplement: Supplementary file 1 [file toxins-15-00009-s001.zip › toxins-2099991-supplementary/Supplementary Material/Projections/Latitudinal_habitat_suitability/lat_hab_seriata_85.tiff]

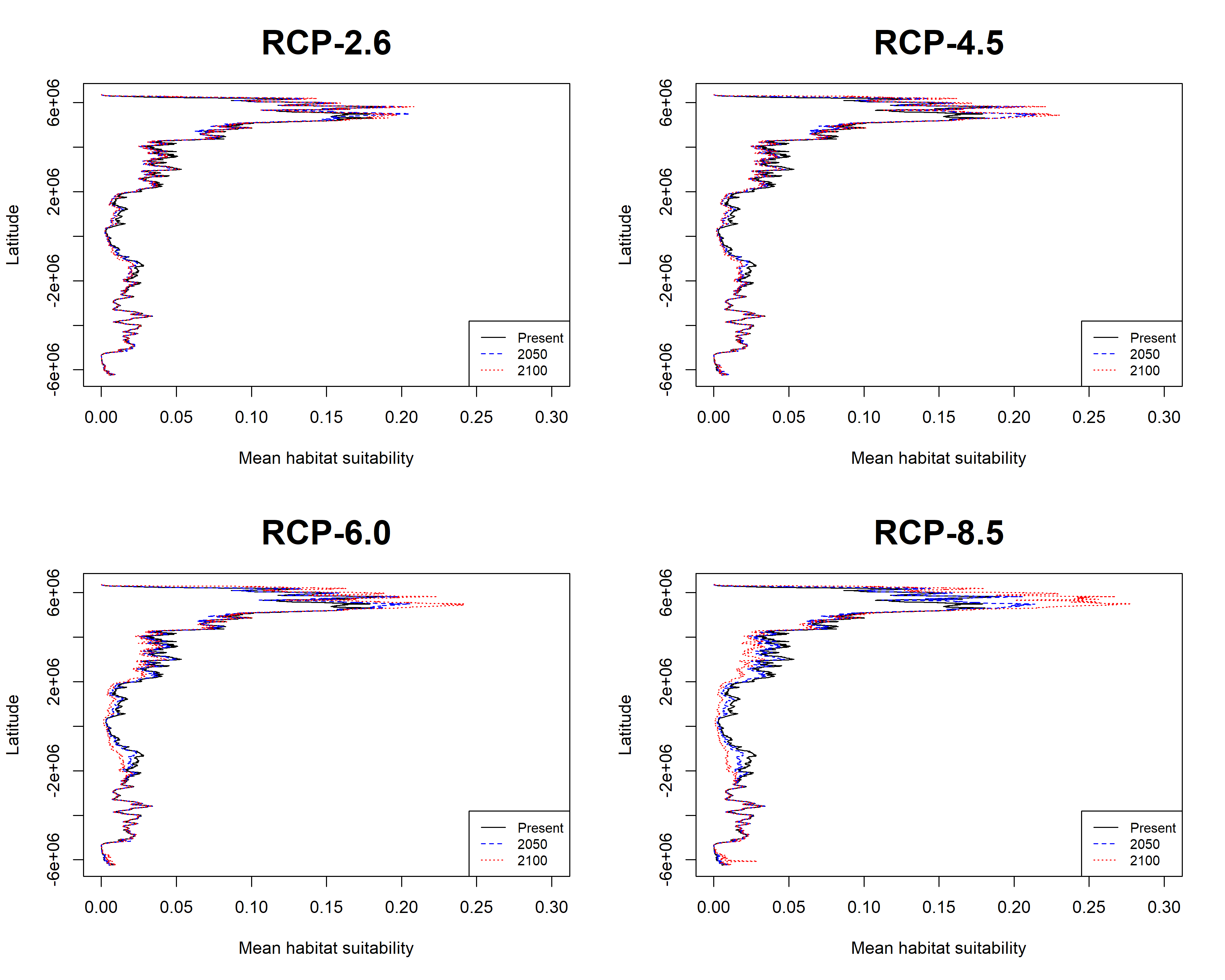

Supplement: Supplementary file 1 [file toxins-15-00009-s001.zip › toxins-2099991-supplementary/Supplementary Material/Projections/Latitudinal_habitat_suitability/lat_hab_seriata_full.tiff]
